# Supplementary material for: Visualizing maturation factor extraction from the nascent ribosome by the AAA-ATPase Drg1
Source: Nat Struct Mol Biol. 2022 Sep 12;29(9):942–53. doi: 10.1038/s41594-022-00832-5 (PMC9507969; doi:10.1038/s41594-022-00832-5)
Supplement: Supplementary file 1 — Supplementary Table 2 [file 41594_2022_832_MOESM1_ESM.pdf]

nature portfolio

https://doi.org/10.1038/s41594-022-00832-5

Supplementary information

Visualizing maturation factor extraction from the nascent ribosome by the AAA-ATPase Drg1

In the format provided by the authors and unedited

Title

Visualizing maturation factor extraction from the nascent ribosome by the AAA-ATPase Drg1

Authors

Michael Prattes[1,2]*, Irina Grishkovskaya[3]*, Victor-Valentin Hodirnau[4], Christina Hetzmannseder[1], Gertrude Zisser[1], Carolin Sailer[5], Vasileios Kargas[6,7,8], Mathias Loibl[1], Magdalena Gerhalter[1], Lisa Kofler[1], Alan J. Warren[6,7,8], Florian Stengel[5], David Haselbach[3,] and Helmut Bergler[1,2,9]

*These authors contributed equally.

Correspondence should be addressed to D.H. (david.haselbach@imp.ac.at) or H.B. (helmut.bergler@uni-graz.at).

Affiliations

[1]Institute of Molecular Biosciences, University of Graz, Austria

[2]BioTechMed-Graz, Graz, Austria

[3]Research Institute of Molecular Pathology (IMP), Vienna BioCenter, Vienna, Austria

[4]Institute of Science and Technology Austria, Klosterneuburg, Austria

[5]Department of Biology, University of Konstanz, Konstanz, Germany

[6]Cambridge Institute for Medical Research, Cambridge Biomedical Campus Keith Peters Building, Hills Rd, Cambridge CB2 0XY, United Kingdom.

[7]Wellcome Trust-Medical Research Council Stem Cell Institute, Jeffrey Cheah Biomedical Centre, Puddicombe Way, Cambridge Biomedical Campus, Cambridge, CB2 0AW, UK.

[8]Department of Haematology, University of Cambridge School of Clinical Medicine, Jeffrey Cheah Biomedical Centre, Puddicombe Way, Cambridge Biomedical Campus, Cambridge, CB2 0AW, UK.

[9]Field of Excellence BioHealth - University of Graz, Graz, Austria

Supplementary tables

Supplementary table 2: Strains, materials and reagents

	SOURCE	IDENTIFIER
**Antibodies**		
Rabbit α-Cbp	Sigma - Aldrich	Cat# SAB4500455
Rabbit α-Crm1	C. Yam	N/A
Rabbit α-Drg1	[1]	N/A
Rabbit α-Mex67	E. Hurt	N/A
Rabbit α-Mrt4	E. Hurt	N/A
Rabbit α-Nmd3	A. W. Johnson	N/A
Rabbit α-Nog1	M. Fromont-Racine	N/A
Rabbit α-Nog2	M. Fromont-Racine	N/A
Rabbit α-Ytm1 (α-Nop7)	J. d. l. Cruz	N/A
Rabbit α-Nsa2	M. Fromont-Racine	N/A
Rabbit α-Rlp24	M. Fromont-Racine	N/A
Rabbit α-Rpl10	B. L. Trumpower	N/A
Rabbit α-Rpl16	S. Rospert	N/A
Rabbit α-Rsa4	M. Remacha	N/A
Peroxidase-conjugated Goat α-Rabbit IgG Antibody (sec. AB)	Sigma - Aldrich	Cat# A0545
**Bacterial strains**		
E. coli BL21 Codon Plus (DE3)-RIPL	Agilent Technologies	Cat# 230280
E. coli Rosetta™(DE3)pLysS	Novagen/Sigma Aldrich	Cat# 70956
**Yeast strains**		
**Drg1 expression strain**: *S. cerevisiae* BY4743 Δ*drg1/DRG1 (MATa/MATα his3/his3 leu2/leu2 met15/MET15 LYS2/lys2 ura3/ura3 YLR397c::kanMX4/YLR397c)*	Euroscarf	Acc.-No.: Y25306
**LmB sensitive Bud20-TAP strain:** *S. cerevisiae* C303 Bud20-TAP LmB^{S} (*MATa, ADE2, leu2, his3, trp1, ura3, Bud20-TAP::HISMX, crm1*^{T539C}*::KanMX*)	This study	N/A
**LmB sensitive Δ*arx1*/Bud20-TAP strain:** *S. cerevisiae* C303 Bud20-TAP LmB^{S} *Δarx1* (*MATa, ADE2, leu2, his3, trp1, ura3, Bud20-TAP::HISMX, Δarx1::KanMX, crm1*^{T539C}*::KanMX*)	This study	N/A
**LmB sensitive Bud20-GFP strain:** *S. cerevisiae* (*Mata ura3; leu2; his3; trp1; crm1T539C::KanMX; BUD20-GFP::HISMX)*	This study	N/A

**Δ*drg1* shuffle strain:**<br>*S. cerevisiae MATa ura3 leu2 his3 lys2 trp1 drg1::kanMX4 [pRS316-DRG1]*	[2]	N/A
**Δ*rlp24* shuffle strain***:*<br>*S. cerevisiae Matα; ura3; leu2; his3; lys2; met15; rlp24::KanMX [pRS316-RLP24]*	This study	N/A
**Δ*drg1/Δarx1* double shuffle strain:**<br>*S. cerevisiae Mata; ura3; leu2; his3; trp1; lys2; Δarx1::KanMX, Δdrg1::KanMX [pRS316-DRG1]*	This study	N/A
ARX1-TAP/drg1-18 strain:<br>*S. cerevisiae* W303 *MATα ura3Δ0 his3Δ1 leu2Δ0 trp1 drg1-18 ARX1-TAP::TRP1MX6*	[3]	N/A
**Reagents and chemicals**		
5-Fluoroorotic acid (5-FOA)	Thermo Scientific	Cat.-Nr. R0812
Leptomycin B (LmB)	LC-Laboratories	CAS Number: [87081-35-4]
Protease-Inhibitor-Mix FY	Serva	Cat# 39104.03
Protease-Inhibitor-Mix HP	Serva	Cat# 39106.03
cOmplete™, EDTA-free Protease Inhibitor Cocktail	Roche	5056489001
TEV protease	Self-made	N/A
PreScission protease	GE Healthcare/Cytiva	Cat# 10196324
GSH-agarose beads	Sigma Aldrich	Cat#: G4510
Magnetic beads	self-made	N/A
Calmodulin Sepharose 4B resin	GE healthcare	GE17-0529-01, VWR Cat#: 95016-874
Nickel-NTA agarose resin	Qiagen	Cat# 30250
ATPγS	Jena Biosciences	Cat# NU-406
ATP	Sigma Aldrich	Cat# 10519979001
IPTG	Thermo Scientific™	Cat# R0392
Bio-Rad Protein Assay Dye Reagent Concentrate	Biorad	Cat#: 5000006
GSH (L-Glutathione reduced)	Sigma Aldrich	Cat#: G4251
NuPAGE™ 4-12% Bis-Tris gel	Invitrogen	Cat# 10338442
Zeba™ Spin Desalting Columns, 7K MWCO, 2 mL	Thermo Fisher Scientific	Cat#: 89890
Sensor chip CM5	Cytiva	BR100012
Sensor chip CM3	Cytiva	BR100541
Amine coupling kit	Cytiva	BR100050
10 mM acetate pH 5.0 coupling buffer	Cytiva	BR100351

Rabbit IgGs	Sigma Aldrich	I5006-10MG
R1.2/1.3 copper grids	Quantifoil	N/A
Crosslinker reagent A DSS-d_0/DSS-d_{12} (disuccinimidyl suberate)	Creative Molecules	Cat.-Nr.: 001S
Crosslinker reagent B BS3-d_0/BS3-d_{12} (bis(sulfosuccinimidyl)suberate)	Creative Molecules	Cat.-Nr.: 001SS
Malachite green phosphate assay	BioAssay Systems	Cat#: POMG-25H
**Deposited data**		
Drg1-pre60S complex	This study	PDB: 7Z34, EMD-14471
Substrate-engaged Drg1 hexamer	This study	PDB: 7Z11, EMD-14437
Raw cryo-EM micrographs	This study	EMPIAR-11053
Early cytoplasmic pre-60S particle coordinates	[4,5]	PDB ID: 6RZZ, 6N8K, 6K8K
L12 coordinates	[6]	PDB ID: 4V6I
Eukaryote-specific rRNA expansion segment ES27	[6]	PDB ID: 3IZD
Drg1-pre-60S (Arx1-TAP) crosslinking data	This study	ProteomeXchange Consortium via the PRIDE partner repository: PXD032098
**Oligonucleotides**		
Rlp24_fwd_BamHI: Fwd-primer for *RLP24:* TATATGGATCCATGAGAATTTATCAATGCCATTTTTG	[7]	N/A
Rlp24C_fwd_BamHI: Fwd-primer for the *RLP24C* domain: TATATGGATCCAAAGAACAAGAAAGAGCTGAATCAGT	This study	N/A
Rlp24ΔC-domain_rev: deletion of the *RLP24* C-domain TATACTCGAGTCATTTAGCCAACTTTCTGGCAATT	This study	N/A
Rlp24_Δtail_rev: Deletion of the C-terminal tail of *RLP24*: TATATGTCGACCTATTGCTTTTCCAATTGTTCTTCCTCT	This study	N/A
Rlp24_rev_SalI: Cloning of *RLP24* in pRS315 incl. promotor/terminator: TATATGTCGACTACCTTTGATAAGAATGTAAGAGGACCC	This study	N/A
Rlp24_fwd_BamHI: Cloning of *RLP24* in pRS315 incl. promotor/terminator: TATAGGATCCCGAGTGAGTCCTACGTTGATT	This study	N/A

Rlp24_RKK_E_fwd: Mutagenesis of the *RLP24* C-terminal tail (R191/K195/K196 to E): CTAAAGAACAGAGAAAGAAATACAGAGGAAATTGCTTTTTAG	This study	N/A
Rlp24_RKK_E_rev: Mutagenesis of the *RLP24* C-terminal tail (R191/K195/K196 to E): TATATCTCGAGCTAAAAAGCAATTTCCTCTGTATTTC TTTCTCTGTTCTTTAG	This study	N/A
Drg1ΔN20_fwd, N-terminal truncation of Drg1 TATAggatccGCTGATGCAAAAGCATCCAAA	This study	N/A
Drg1ΔN28_fwd, N-terminal truncation of Drg1 TATAggatccAAATTGCCTGCTGAATTTATT	This study	N/A
**Plasmids**		
Drg1 expression plasmid **pCUP1-*DRG1*** (pAZ7) (aa 1-780; N-terminal GST-fusion)	[1]	N/A
Drg1 expression plasmid **pCUP1-*drg1-EQ1*** (E346Q) (aa 1-780, N-terminal GST-fusion)	[7]	N/A
Drg1 expression plasmid **pCUP1-*drg1-ΔN20*** (aa 21-780, N-terminal GST-fusion)	This study	N/A
Drg1 expression plasmid **pCUP1-*drg1-ΔN28*** (aa 29-780, N-terminal GST-fusion)	This study	N/A
Arx1 expression plasmid **pGEX-*ARX1*** (aa 1-593)	This study	N/A
Rlp24 expression plasmid **pET32-*RLP24***; HIS_6-Rlp24 (aa 1-199)	[7]	N/A
Rlp24 expression plasmid **pET32-*rlp24C***; HIS_6-Rlp24C (aa 147-199)	[7]	N/A
Rlp24 expression plasmid **pET32-*rlp24CΔtail***; HIS_6-Rlp24CΔtail (aa 147-183)	This study	N/A
Rlp24 expression plasmid **pET32-*rlp24-RKK>E***; HIS_6-Rlp24C (aa 147-199); Exchange of R191, K195 and K196 for glutamate)	This study	N/A
Rlp24 expression plasmid **pGEX-*rlp24C***; GST-Rlp24 C-domain (aa 147-199)	[7]	N/A
Rlp24 expression plasmid **pGEX-*rlp24CΔtail***; GST-Rlp24CΔtail (aa 147-183)	This study	N/A
Rlp24 expression plasmid **pGEX-*rlp24-RKK>E***; GST-Rlp24C (aa 147-199; Exchange of R191, K195 and K196 for glutamate)	This study	N/A

**pRS315-*RLP24*** RLP24 CDS with endogenous promotor and terminator	This study	N/A
**pRS315-*rlp24ΔC*** CDS (aa 1-146) with endogenous promotor and terminator	This study	N/A
**pRS315-*rlp24Δtail*** CDS (aa 1-183) with endogenous promotor and terminator	This study	N/A
**pRS315-*rlp24-RKK>E*** CDS (aa 1-199) with endogenous promotor and terminator	This study	N/A
**pRS315** (empty vector)	[8]	N/A
**pRS315-*DRG1*** CDS (aa 1-780) with endogenous promotor and terminator	[9]	N/A
**pRS315-*drg1-K117E/K118E***, CDS (aa 1-780) with endogenous promotor and terminator	This study	N/A
**pRS315-*drg1-K218E/R219E***, CDS (aa 1-780) with endogenous promotor and terminator	This study	N/A
**pRS315-*drg1-K117E/K118E/K218E/R219E***, CDS (aa 1-780) with endogenous promotor and terminator	This study	N/A
**pRS315-*drg1⊿N28***, CDS (aa 29-780) with endogenous promotor and terminator	This study	N/A
**pRS315-*drg1⊿N20***, CDS (aa 21-780) with endogenous promotor and terminator	This study	N/A
**pRS313-*ARX1***, CDS (aa 1-593) with endogenous promotor and terminator	This study	N/A
**pRS313** (empty vector)	[8]	N/A
**Software and algorithms**		
Biacore X100 control software v2.0.2	Cytiva	N/A
Biacore X100 evaluation software v.2.0.2	Cytiva	N/A
Coot v0.9.2/v0.9.6	[10]	N/A
CryoDRGN v0.3.2	[11]	N/A
Cryosparc v3.0	[12–14]	N/A
DeepEMhancer	[15]	N/A
Graphpad prism V3.03	GraphPad	N/A
ImageLab v2.2.0.08	BioRad	N/A
SerialEM v3.8	Mastronade group, University of Colorado	N/A
ISOLDE v.1.2.2	[16]	N/A
Microsoft Excel 2019 (+XFluor4 v4.51 plugin)	Microsoft	N/A
PHENIX suite v1.18.2-3874	[17]	N/A

RELION v3.0	[18]	N/A
Rosetta v3.0	Rosetta Commons	N/A
UCSF Chimera v.1.15	[19,20]	N/A
UCSF ChimeraX v1.25	[21,22]	N/A
UCSF pyem v0.5	[23]	N/A
xiNet v1.1.13	[24]	N/A
xQuest/xProphet v2.1.5	[25]	N/A

Supplemental references

1. Zakalskiy, A. *et al.* Structural and enzymatic properties of the AAA protein Drg1p from Saccharomyces cerevisiae. Decoupling of intracellular function from ATPase activity and hexamerization. *J. Biol. Chem.* **277**, 26788–26795 (2002).
2. Loibl, M. *et al.* The drug diazaborine blocks ribosome biogenesis by inhibiting the AAA-ATPase Drg1. *J. Biol. Chem.* **289**, 3913–3922 (2014).
3. Pertschy, B. *et al.* Cytoplasmic recycling of 60S preribosomal factors depends on the AAA protein Drg1. *Mol. Cell. Biol.* **27**, 6581–6592 (2007).
4. Kargas, V. *et al.* Mechanism of completion of peptidyltransferase centre assembly in eukaryotes. *eLife* **8**, e44904 (2019).
5. Zhou, Y., Musalgaonkar, S., Johnson, A. W. & Taylor, D. W. Tightly-orchestrated rearrangements govern catalytic center assembly of the ribosome. *Nature Communications* **10**, 958 (2019).
6. Armache, J.-P. *et al.* Cryo-EM structure and rRNA model of a translating eukaryotic 80S ribosome at 5.5-Å resolution. *PNAS* **107**, 19748–19753 (2010).
7. Kappel, L. *et al.* Rlp24 activates the AAA-ATPase Drg1 to initiate cytoplasmic pre-60S maturation. *J. Cell Biol.* **199**, 771–782 (2012).
8. Sikorski, R. S. & Hieter, P. A system of shuttle vectors and yeast host strains designed for efficient manipulation of DNA in Saccharomyces cerevisiae. *Genetics* **122**, 19–27 (1989).
9. Prattes, M. *et al.* A conserved inter-domain communication mechanism regulates the ATPase activity of the AAA-protein Drg1. *Scientific Reports* **7**, srep44751 (2017).
10. Emsley, P. & Cowtan, K. Coot: model-building tools for molecular graphics. *Acta Crystallogr D Biol Crystallogr* **60**, 2126–2132 (2004).

11. Zhong, E. D., Bepler, T., Berger, B. & Davis, J. H. CryoDRGN: reconstruction of heterogeneous cryo-EM structures using neural networks. *Nat Methods* **18**, 176–185 (2021).
12. Punjani, A., Rubinstein, J. L., Fleet, D. J. & Brubaker, M. A. cryoSPARC: algorithms for rapid unsupervised cryo-EM structure determination. *Nature Methods* **14**, 290–296 (2017).
13. Punjani, A., Zhang, H. & Fleet, D. J. Non-uniform refinement: adaptive regularization improves single-particle cryo-EM reconstruction. *Nature Methods* **17**, 1214–1221 (2020).
14. Punjani, A. & Fleet, D. J. 3D Variability Analysis: Resolving continuous flexibility and discrete heterogeneity from single particle cryo-EM. *bioRxiv* 2020.04.08.032466 (2021) doi:10.1101/2020.04.08.032466.
15. Sanchez-Garcia, R. *et al.* DeepEMhancer: a deep learning solution for cryo-EM volume post-processing. *bioRxiv* 2020.06.12.148296 (2020) doi:10.1101/2020.06.12.148296.
16. Croll, T. I. ISOLDE: a physically realistic environment for model building into low-resolution electron-density maps. *Acta Cryst D* **74**, 519–530 (2018).
17. Adams, P. D. *et al.* PHENIX: a comprehensive Python-based system for macromolecular structure solution. *Acta Crystallogr D Biol Crystallogr* **66**, 213–221 (2010).
18. Scheres, S. H. W. RELION: implementation of a Bayesian approach to cryo-EM structure determination. *J Struct Biol* **180**, 519–530 (2012).
19. Goddard, T. D., Huang, C. C. & Ferrin, T. E. Visualizing density maps with UCSF Chimera. *Journal of Structural Biology* **157**, 281–287 (2007).
20. Pettersen, E. F. *et al.* UCSF Chimera--a visualization system for exploratory research and analysis. *J Comput Chem* **25**, 1605–1612 (2004).
21. Goddard, T. D. *et al.* UCSF ChimeraX: Meeting modern challenges in visualization and analysis. *Protein Sci* **27**, 14–25 (2018).
22. Pettersen, E. F. *et al.* UCSF ChimeraX: Structure visualization for researchers, educators, and developers. *Protein Sci* **30**, 70–82 (2021).
23. Daniel Asarnow, Eugene Palovcak & Yifan Cheng. *asarnow/pyem: UCSF pyem v0.5*. (Zenodo, 2019). doi:10.5281/zenodo.3576630.

24. Combe, C. W., Fischer, L. & Rappsilber, J. xiNET: Cross-link Network Maps With Residue Resolution. *Mol Cell Proteomics* **14**, 1137–1147 (2015).
25. Leitner, A., Walzthoeni, T. & Aebersold, R. Lysine-specific chemical cross-linking of protein complexes and identification of cross-linking sites using LC-MS/MS and the xQuest/xProphet software pipeline. *Nat. Protocols* **9**, 120–137 (2014).
