# Supplementary material for: Visualizing maturation factor extraction from the nascent ribosome by the AAA-ATPase Drg1
Source: Nat Struct Mol Biol. 2022 Sep 12;29(9):942–53. doi: 10.1038/s41594-022-00832-5 (PMC9507969; doi:10.1038/s41594-022-00832-5)
Supplement: Supplementary file 2 — Reporting Summary [file 41594_2022_832_MOESM2_ESM.pdf]

nature research

Corresponding author(s): Helmut Bergler and David Haselbach

Last updated by author(s): May 30, 2022

Reporting Summary

Nature Research wishes to improve the reproducibility of the work that we publish. This form provides structure for consistency and transparency in reporting. For further information on Nature Research policies, see our Editorial Policies and the Editorial Policy Checklist.

Statistics

For all statistical analyses, confirm that the following items are present in the figure legend, table legend, main text, or Methods section.

n/a	Confirmed	
☐	☒	The exact sample size (*n*) for each experimental group/condition, given as a discrete number and unit of measurement
☐	☒	A statement on whether measurements were taken from distinct samples or whether the same sample was measured repeatedly
☒	☐	The statistical test(s) used AND whether they are one- or two-sided<br>*Only common tests should be described solely by name; describe more complex techniques in the Methods section.*
☒	☐	A description of all covariates tested
☒	☐	A description of any assumptions or corrections, such as tests of normality and adjustment for multiple comparisons
☐	☒	A full description of the statistical parameters including central tendency (e.g. means) or other basic estimates (e.g. regression coefficient) AND variation (e.g. standard deviation) or associated estimates of uncertainty (e.g. confidence intervals)
☒	☐	For null hypothesis testing, the test statistic (e.g. *F*, *t*, *r*) with confidence intervals, effect sizes, degrees of freedom and *P* value noted<br>*Give P values as exact values whenever suitable.*
☒	☐	For Bayesian analysis, information on the choice of priors and Markov chain Monte Carlo settings
☒	☐	For hierarchical and complex designs, identification of the appropriate level for tests and full reporting of outcomes
☒	☐	Estimates of effect sizes (e.g. Cohen's *d*, Pearson's *r*), indicating how they were calculated

Our web collection on statistics for biologists contains articles on many of the points above.

Software and code

Policy information about availability of computer code

Data collection	Raw data (OD600) of the ATPase activity measurements were collected on a Tecan plate reader using an associated Microsoft excel macro plugin (XFluor4 v4.51). Cryo-EM data were recorded with SerialEM v3.8 (https://bio3d.colorado.edu/SerialEM/). SPR data were collected using the Bicaore X100 control software v2.0.2 (Cytiva). Western blots and SDS gels were imaged on a ChemiDoc Touch imaging system (Biorad).
Data analysis	Analysis of biochemical/biophysical data (ATPase activity and SPR measurements): Microsoft Excel 2019 and the Graphpad Prism software v 3.03, Biacore X100 evaluation software (SPR). Western blot analysis: ImageLab software v.2.2.0.08 (BioRad). Crosslinking MS: xiNET v1.1.13 (Rappsilber Laboratory) and xQUest/xProphet v2.1.5 (https://bioinformaticshome.com).<br>Cryo-EM structure analysis: Coot v0.9.2/v0.9.6, CryoDRGN v0.3.2 (https://github.com/zhonge/cryodrgn), Cryosparc v3.0, DeepEMhancer (https://github.com/rsanchezgarc/deepEMhancer), Isolde v.1.2.2 (https://github.com/tristanic/isolde), PHENIX suite v1.18.2-3874, RELION v3.0, Rosetta v3.0, UCSF Chimera v.1.15, UCSF ChimeraX v1.1.1, UCSF pyem v0.5.

For manuscripts utilizing custom algorithms or software that are central to the research but not yet described in published literature, software must be made available to editors and reviewers. We strongly encourage code deposition in a community repository (e.g. GitHub). See the Nature Research guidelines for submitting code & software for further information.

Data

Policy information about availability of data

All manuscripts must include a data availability statement. This statement should provide the following information, where applicable:

- Accession codes, unique identifiers, or web links for publicly available datasets
- A list of figures that have associated raw data
- A description of any restrictions on data availability

Cryo-EM maps and coordinate models generated in this study were deposited in the PDB as well as EMDB databases: Substrate-bound Drg1 hexamer (PDB: 7Z11,

April 2020

EMDB: EMD-14437) and Drg1-bound to the Bud20-TAP pre-ribosomal particle (PDB: 7Z34, EMDB: EMD-14471). Cryo-EM raw data (unprocessed micrographs) are deposited in the EMPIAR database (accession code EMPIAR-11053). Additional published datasets used in this study are available from the PDB: For the Bud20-TAP particle, published components of early cytoplasmic pre-60S particles (PDB 6RZZ, 28; (Arx1), PDB 6N8K, 27; (25S rRNA, Mrt4, Nog1, Bud20, Rlp24 and YBl028C) and PDB 6K8K (Nmd3) were used as initial models. An initial model for L12 was taken from the mature 80S ribosome (PDB 4V6I). As initial model for the ES27 rRNA segment PDB 3IZD was used. The MS raw files, the crosslink database and original xQuest result files have been deposited to the ProteomeXchange Consortium via the PRIDE partner repository with the dataset identifier PXD032098. Source data for the graphs and calculated parameters in figures 1a, 1b, 3c-e and 3g-i, 6a-d and extended data figures 3a-d are provided with this paper as Source data files.

Field-specific reporting

Please select the one below that is the best fit for your research. If you are not sure, read the appropriate sections before making your selection.

☒ Life sciences ☐ Behavioural & social sciences ☐ Ecological, evolutionary & environmental sciences

For a reference copy of the document with all sections, see nature.com/documents/nr-reporting-summary-flat.pdf

Life sciences study design

All studies must disclose on these points even when the disclosure is negative.

Sample size	Sample sizes (n) are supplied in the figure legends as well as the methods details. No mathematical sample size calculation was performed. All biochemical and yeast growth experiments were performed with multiple biological and technical replicates to allow estimation of the distribution of the data. Sample sizes are based on preliminary and published studies (Kappel et al., 2012, Loibl et al., 2014, Prattes et al., 2017, 2021) and were determined by number of replicates necessary to ensure reproducibility. Detailed information for the individual experiments including sample size and replicates are stated in the figure legends, the methods section as well as the source data file provided along with this paper.
Data exclusions	Individual cryo-EM micrographs were discarded due to strong drift, devitrification or ice contamination after manual inspection. For the biochemical measurements and yeast growth experiments no datasets were excluded.
Replication	For all biochemical measurements, 2-4 biological replicates were tested, each measured with at least two technical replications. All attempts at replication were successfull. Detailed information for the individual experiments, including exact sample number (n) are stated in the figure legends, the methods section as well as the source data file provided along with this paper.
Randomization	For calculation of the Fourier Shell Correlation using Cryosparc, the cryo-EM particles were automatically split into two random halves by the software. For biochemical/biophysical analyses, samples from each biological replicate were randomly assigned to the tested conditions. For growth comparison experiments of different yeast strains, randomization was not applicable.
Blinding	For biophysical/biochemical and yeast growth experiments, the same investigator performed data collection and/or analysis. Data acquisition and analyses were performed using constant parameters across all conditions being tested. The quantitative readout of the biochemical/ biophysical assays in this study did not require subjective interpretation of the results and thus blinding was not applied.

Reporting for specific materials, systems and methods

We require information from authors about some types of materials, experimental systems and methods used in many studies. Here, indicate whether each material, system or method listed is relevant to your study. If you are not sure if a list item applies to your research, read the appropriate section before selecting a response.

Materials & experimental systems

n/a	Involved in the study	
☐	☒	Antibodies
☒	☐	Eukaryotic cell lines
☒	☐	Palaeontology and archaeology
☒	☐	Animals and other organisms
☒	☐	Human research participants
☒	☐	Clinical data
☒	☐	Dual use research of concern

Methods

n/a	Involved in the study	
☒	☐	ChIP-seq
☒	☐	Flow cytometry
☒	☐	MRI-based neuroimaging

Antibodies

Antibodies used	Rabbit α-Cbp: Sigma - Aldrich Cat# SAB4500455; Rabbit α-Crm1 (1:10,000): C. Yan (Yan et al., 1998); Rabbit α-Drg1 (1:5,000): Zakalskiy et al., 2002; Rabbit α-Mex67 (1:10,000): E. Hurt/Segref et al., 1997; Rabbit α-Mrt4 (1:1,000): Jesus de la Cruz (Rodriguez-Mateos et al., 2009); Rabbit α-Nmd3 (1:4,000): A. W. Johnson (Kallstrom et al., 2003); Rabbit α-Nog1 (1:5,000): M. Fromont-Racine (Saveanu et al., 2003); Rabbit α-Nog2(1:5,000): M. Fromont-Racine (Saveanu et al., 2001); Rabbit combined α-Ytm1 and α-Nop7(1:5,000): J. d. l. Cruz (Wegrecki et al., 2015); Rabbit α-Nsa2: (1:5,000) M. Fromont-Racine (Lebreton et al., 2006); Rabbit α-Rlp24 (1.5,000): M. Fromont-Racine (Saveanu et al., 2001); Rabbit α-Rpl10 (1:10,000): B. L. Trumpower (Eisinger et al., 1997); Rabbit

α-Rpl16 (1:40,000): S. Rospert (Peisker et al., 2008); Rabbit α-Rsa4 (1:10,000): M. Remacha (de la Cruz et al., 2005); Peroxidase conjugated Goat α-Rabbit IgG Antibody (sec. AB; 1:10,000): Sigma - Aldrich Cat# A0545

Validation	Commercially available antibodies were validated by the manufacturer. All antibodies are from published studies and were used previously (Pertschy et al., 2007, Kappel et al., 2012, Bassler et al., 2012, Zisser et al., 2018).
