# Supplementary material for: Visualizing maturation factor extraction from the nascent ribosome by the AAA-ATPase Drg1
Source: Nat Struct Mol Biol. 2022 Sep 12;29(9):942–53. doi: 10.1038/s41594-022-00832-5 (PMC9507969; doi:10.1038/s41594-022-00832-5)
Supplement: Supplementary file 6 — Crosslinking MS data [file 41594_2022_832_MOESM6_ESM.pdf]

Table S1 Drg1-preribosome crosslinks

Arx1 pre-ribosomal particle with additional Drg1

	Fraction	Raw file	Intensity	MS/MS Scans	features with mass shift 12.075321	Annot. with FDR
**Experiment 1**	1	F1905_sailerc_149	1,00E+10	39627	14147	19417
**BS3**	1	F1905_sailerc_150	2,50E+10	39982	14603	
	2	F1905_sailerc_151	2,00E+10	45212	18666	
	2	F1905_sailerc_152	2,00E+10	44820	18375	
	3	F1905_sailerc_153	3,00E+10	33550	9689	
	3	F1905_sailerc_154	2,00E+10	33100	9479	
	4	F1905_sailerc_155	2,00E+10	19776	3678	
	4	F1905_sailerc_156	2,00E+10	19479	3622	

	Fraction	Raw file	Intensity	MS/MS Scans	features with mass shift 12.075321	Annot. with FDR
**Experiment 1**	1	F1905_sailerc_139	1,00E+10	37115	13281	19000
**DSS**	1	F1905_sailerc_140	1,00E+10	36728	13001	
	2	F1905_sailerc_141	3,00E+10	43603	17911	
	2	F1905_sailerc_142	3,00E+10	43026	17769	
	3	F1905_sailerc_143	3,00E+10	34020	9981	
	3	F1905_sailerc_144	3,00E+10	33672	9898	
	4	F1905_sailerc_145	1,90E+10	21012	4389	
	4	F1905_sailerc_146	2,00E+10	20764	7374	

Experiment 1: Crosslinking with BS3

ID > 32

inter-protein crosslinks

Crosslinked pept	Protein1	Protein2	Type	XLType	Position 1	Position 2	deltaAA	Annotation	Mr	Mz	z	Error_rel[ppn	nseen	mions	deltaS	Id-Score	FDR	uxID
GVVWTESHQEAD	sp|Q03862|AR	sp|P47019|ALB1_YEAST	xlink	inter-protein xl	285	137	148	F1905_sailerc	4010.094	803.027	5.000	2.4	3	5	0.50	46.18	0.000	sp|P47019|ALB1_YEAST:137:x:sp|Q03862|ARX1_YEAST:285
LALVIKSGK-DALLI	sp|P14120|RL3	sp|P0CX82|RL19A_YEAST	xlink	inter-protein xl	19	186	167	F1905_sailerc	1939.128	647.384	3.000	2.2	5	9	0.67	43.40	0.000	sp|P0CX82|RL19A_YEAST:186:x:sp|P14120|RL30_YEAST:19
IFEFPKTTVAALR-I	sp|P0CX49|RL1	sp|P02406|RL28_YEAST	xlink	inter-protein xl	98	92	6	F1905_sailerc	2668.452	668.121	4.000	1.5	5	8	0.80	42.16	0.000	sp|P02406|RL28_YEAST:92:x:sp|P0CX49|RL18A_YEAST:98
IVKAGGECITLDQI	sp|P0CX49|RL1	sp|P02406|RL28_YEAST	xlink	inter-protein xl	116	92	24	F1905_sailerc	3018.611	1007.211	3.000	1.7	5	5	0.40	41.69	0.000	sp|P02406|RL28_YEAST:92:x:sp|P0CX49|RL18A_YEAST:116
VHSLNKK-LMKQS	sp|P47019|ALE	sp|P38202|YBC8_YEAST	xlink	inter-protein xl	25	95	70	F1905_sailerc	1695.971	425.001	4.000	0.3	4	10	0.78	40.84	0.000	sp|P38202|YBC8_YEAST:95:x:sp|P47019|ALB1_YEAST:25
NLKYATQR-AKAL	sp|P47019|ALE	sp|P38061|RL32_YEAST	xlink	inter-protein xl	102	113	11	F1905_sailerc	1830.076	458.527	4.000	1.8	2	9	0.69	39.97	0.167	sp|P38061|RL32_YEAST:113:x:sp|P47019|ALB1_YEAST:102
KLEIQQHAR-AKYI	sp|P0CX25|RL4	sp|P39715|ECM1_YEAST	xlink	inter-protein xl	28	135	107	F1905_sailerc	2252.242	564.068	4.000	1.3	4	10	0.83	39.81	0.167	sp|P0CX25|RL43A_YEAST:28:x:sp|P39715|ECM1_YEAST:135
SSVNSKSGEIK-IA	sp|P47019|ALE	sp|P05740|RL17A_YEAST,s	xlink	inter-protein xl	46	180,180	134	F1905_sailerc	1958.082	490.528	4.000	1.2	1	6	0.69	39.42	0.167	sp|P05740|RL17A_YEAST:180:|:sp|P46990|RL17B_YEAST:180:x:sp|P47019|ALB1_YEAST:46
ALKQEGAANK-AL	sp|P0CX49|RL1	sp|P05747|RL29_YEAST	xlink	inter-protein xl	72	58	14	F1905_sailerc	1838.063	613.696	3.000	0.4	4	7	0.89	39.06	0.167	sp|P05747|RL29_YEAST:58:x:sp|P0CX49|RL18A_YEAST:72
LVKALNFTNR-LPS	sp|P39715|ECM	sp|P0CX45|RL2A_YEAST	xlink	inter-protein xl	115	155	40	F1905_sailerc	2012.180	671.735	3.000	0.9	4	6	0.57	38.93	0.167	sp|P0CX45|RL2A_YEAST:155:x:sp|P39715|ECM1_YEAST:115
KVSSASAAASESD	sp|P26785|RL1	sp|P36105|RL14A_YEAST,s	xlink	inter-protein xl	176	137,137	39	F1905_sailerc	2245.218	749.414	3.000	0.9	2	3	0.90	38.73	0.167	sp|P26785|RL16B_YEAST:176:x:sp|P36105|RL14A_YEAST:137:|:sp|P38754|RL14B_YEAST:137
IGLVKYMSEK-KGI	sp|P38344|REI	sp|P04449|RL24A_YEAST,s	xlink	inter-protein xl	205	61,61	144	F1905_sailerc	2278.252	570.571	4.000	1.3	1	11	0.74	38.71	0.167	sp|P04449|RL24A_YEAST:61:|:sp|P24000|RL24B_YEAST:61:x:sp|P38344|REI1_YEAST:205
APKGQNTLILR-SK	sp|P0CX49|RL1	sp|P05737|RL7A_YEAST	xlink	inter-protein xl	133	17	116	F1905_sailerc	2036.174	679.733	3.000	-0.0	4	7	0.93	38.40	0.167	sp|P05737|RL7A_YEAST:17:x:sp|P0CX49|RL18A_YEAST:133
GAVSGQKAGLIA	sp|P0CX45|RL2	sp|P0CX41|RL23A_YEAST	xlink	inter-protein xl	234	66	168	F1905_sailerc	2390.412	598.611	4.000	0.1	2	5	0.93	37.62	0.263	sp|P0CX41|RL23A_YEAST:66:x:sp|P0CX45|RL2A_YEAST:234
KLLVDASAK-AKAI	sp|P47019|ALE	sp|P38061|RL32_YEAST	xlink	inter-protein xl	108	113	5	F1905_sailerc	1781.109	446.285	4.000	3.3	2	8	0.91	37.20	0.263	sp|P38061|RL32_YEAST:113:x:sp|P47019|ALB1_YEAST:108
NEEEDAKNPLFSY	sp|P0C2H8|RL3	sp|P05740|RL17A_YEAST,s	xlink	inter-protein xl	86,86	89,89	3	F1905_sailerc	4644.384	1162.104	4.000	-2.6	1	14	0.56	37.19	0.263	sp|P05740|RL17A_YEAST:89:|:sp|P46990|RL17B_YEAST:89:x:sp|P0C2H8|RL31A_YEAST:86:|:sp|P0C2H9|RL31B_YEAST:86
ICANKYMTTVSGF	sp|P41805|RL1	sp|P33201|MRT4_YEAST	xlink	inter-protein xl	74	8	66	F1905_sailerc	2840.481	711.128	4.000	1.3	2	9	0.71	37.13	0.263	sp|P33201|MRT4_YEAST:8:x:sp|P41805|RL10_YEAST:74
TLTAKALATESGIN	sp|P32794|AF(	sp|Q08287|NOP8_YEAST	xlink	inter-protein xl	568	217	351	F1905_sailerc	3446.960	690.400	5.000	-4.4	2	4	0.79	36.95	0.263	sp|P32794|AFG2_YEAST:568:x:sp|Q08287|NOP8_YEAST:217
LEDEMDIDLDGGI	sp|P47019|ALE	sp|P38112|MAK5_YEAST	xlink	inter-protein xl	129	706	577	F1905_sailerc	2819.397	564.887	5.000	1.4	1	7	0.90	36.70	0.263	sp|P38112|MAK5_YEAST:706:x:sp|P47019|ALB1_YEAST:129
ASKVLEQLSGQTP	sp|P0C0W9|RL	sp|P0CX43|RL1A_YEAST	xlink	inter-protein xl	35,35	130	95	F1905_sailerc	3047.730	762.940	4.000	2.3	2	8	0.85	36.44	0.300	sp|P0C0W9|RL11A_YEAST:35:|:sp|Q3E757|RL11B_YEAST:35:x:sp|P0CX43|RL1A_YEAST:130
KMFFDKK-LVKKN	sp|P40007|NO	sp|Q08746|RRS1_YEAST	xlink	inter-protein xl	198	189	9	F1905_sailerc	1938.101	485.533	4.000	-0.3	1	7	0.94	36.25	0.318	sp|P40007|NOP16_YEAST:198:x:sp|Q08746|RRS1_YEAST:189
TLTAKALATESGIN	sp|P32794|AF(	sp|P25582|SPB1_YEAST	xlink	inter-protein xl	568	6	562	F1905_sailerc	2904.634	969.219	3.000	-0.5	5	4	0.42	36.22	0.318	sp|P25582|SPB1_YEAST:6:x:sp|P32794|AFG2_YEAST:568
DALLKEDA-AAKN	sp|P0CX82|RL1	sp|P36049|EBP2_YEAST	xlink	inter-protein xl	186	379	193	F1905_sailerc	1612.869	538.631	3.000	1.2	2	3	0.66	35.69	0.327	sp|P0CX82|RL19A_YEAST:186:x:sp|P36049|EBP2_YEAST:379
DALLKEDA-KGIKP	sp|P0CX82|RL1	sp|Q08746|RRS1_YEAST	xlink	inter-protein xl	186	124	62	F1905_sailerc	1966.111	656.378	3.000	0.4	1	5	0.88	35.41	0.327	sp|P0CX82|RL19A_YEAST:186:x:sp|Q08746|RRS1_YEAST:124
YVYAHFPINVNIV	sp|P05738|RL9	sp|P38202|YBC8_YEAST	xlink	inter-protein xl	106,106	24	82	F1905_sailerc	3403.797	681.767	5.000	1.1	4	8	0.76	35.07	0.327	sp|P05738|RL9A_YEAST:106:|:sp|P51401|RL9B_YEAST:106:x:sp|P38202|YBC8_YEAST:24
KLLVDASAK-KAA	sp|P47019|ALE	sp|P40024|ARB1_YEAST	xlink	inter-protein xl	108	22	86	F1905_sailerc	1625.973	407.501	4.000	0.9	2	4	0.70	35.03	0.327	sp|P40024|ARB1_YEAST:22:x:sp|P47019|ALB1_YEAST:108
KEANLFPEQQNK-	sp|Q02326|RL6	sp|P47019|ALB1_YEAST	xlink	inter-protein xl	117	37	80	F1905_sailerc	2407.327	602.840	4.000	2.1	2	6	0.79	34.35	0.327	sp|P47019|ALB1_YEAST:37:x:sp|Q02326|RL6A_YEAST:117
SELEYYAMLSKTK-	sp|P14120|RL3	sp|P0C2H6|RL27A_YEAST,s	xlink	inter-protein xl	64	6,6	58	F1905_sailerc	2362.253	788.426	3.000	1.7	1	4	0.67	34.32	0.327	sp|P0C2H6|RL27A_YEAST:6:|:sp|P0C2H7|RL27B_YEAST:6:x:sp|P14120|RL30_YEAST:64
TLTAKALATESGIN	sp|P32794|AF(	sp|P32892|DRS1_YEAST	xlink	inter-protein xl	568	296	272	F1905_sailerc	2916.703	730.184	4.000	3.0	2	4	0.58	34.07	0.327	sp|P32794|AFG2_YEAST:568:x:sp|P32892|DRS1_YEAST:296
LGVSAIKLK-KGVC	sp|Q03862|AR	sp|P38344|REI1_YEAST	xlink	inter-protein xl	484	354	130	F1905_sailerc	2009.197	503.307	4.000	-1.2	2	7	0.74	34.00	0.327	sp|P38344|REI1_YEAST:354:x:sp|Q03862|ARX1_YEAST:484
VTNPKGR-VTKKP	sp|P38061|RL3	sp|P25582|SPB1_YEAST	xlink	inter-protein xl	123	785	662	F1905_sailerc	1607.973	536.999	3.000	0.7	3	7	0.94	33.94	0.327	sp|P25582|SPB1_YEAST:785:x:sp|P38061|RL32_YEAST:123
VTNPKGR-VTKKP	sp|P38061|RL3	sp|P25582|SPB1_YEAST	xlink	inter-protein xl	123	786	663	F1905_sailerc	1607.974	536.999	3.000	1.3	1	7	0.90	33.91	0.327	sp|P25582|SPB1_YEAST:786:x:sp|P38061|RL32_YEAST:123
KLEIQQHAR-GSQ	sp|P0CX25|RL4	sp|P0CX45|RL2A_YEAST	xlink	inter-protein xl	28	251	223	F1905_sailerc	2022.051	675.025	3.000	1.0	5	8	0.80	33.78	0.327	sp|P0CX25|RL43A_YEAST:28:x:sp|P0CX45|RL2A_YEAST:251
GVVWTESHQEAD	sp|Q03862|AR	sp|P26785|RL16B_YEAST	xlink	inter-protein xl	285	81	204	F1905_sailerc	4011.096	1003.782	4.000	0.2	1	3	0.52	33.61	0.327	sp|P26785|RL16B_YEAST:81:x:sp|Q03862|ARX1_YEAST:285
AGKITIDSPYLVCT	sp|P33201|MR	sp|P0CX53|RL12A_YEAST	xlink	inter-protein xl	179	40	139	F1905_sailerc	3126.691	782.681	4.000	0.9	5	7	0.57	33.52	0.327	sp|P0CX53|RL12A_YEAST:40:x:sp|P33201|MRT4_YEAST:179
VSAAAASTSKSAE	sp|P38344|REI	sp|P04449|RL24A_YEAST,s	xlink	inter-protein xl	63	27,27	36	F1905_sailerc	2266.184	567.554	4.000	1.7	1	6	0.76	33.50	0.327	sp|P04449|RL24A_YEAST:27:|:sp|P24000|RL24B_YEAST:27:x:sp|P38344|REI1_YEAST:63
AFLIEEQKIVK-YP	sp|P87262|RL3	sp|P05747|RL29_YEAST	xlink	inter-protein xl	99,99	33	66	F1905_sailerc	2557.448	640.370	4.000	2.6	1	5	0.88	33.42	0.327	sp|P05747|RL29_YEAST:33:x:sp|P40525|RL34B_YEAST:99:|:sp|P87262|RL34A_YEAST:99
KSSASSNSADAK-I	sp|P32794|AF(	sp|Q04660|ERB1_YEAST	xlink	inter-protein xl	13	7	6	F1905_sailerc	2194.065	732.363	3.000	-3.0	4	5	0.78	33.32	0.327	sp|P32794|AFG2_YEAST:13:x:sp|Q04660|ERB1_YEAST:7
ASKVLEQLSGQTP	sp|P0C0W9|RL	sp|P0CX43|RL1A_YEAST	xlink	inter-protein xl	35,35	91	56	F1905_sailerc	3460.748	866.195	4.000	1.5	3	11	0.69	33.13	0.327	sp|P0C0W9|RL11A_YEAST:35:|:sp|Q3E757|RL11B_YEAST:35:x:sp|P0CX43|RL1A_YEAST:91
EEPKKEEPEQLTE	sp|P38344|REI	sp|Q07953|SDO1_YEAST	xlink	inter-protein xl	133	25	108	F1905_sailerc	3909.837	978.467	4.000	-0.8	5	3	0.51	33.11	0.327	sp|P38344|REI1_YEAST:133:x:sp|Q07953|SDO1_YEAST:25
KFNTEESGVDLHE	sp|P32794|AF(	sp|P53145|LSG1_YEAST	xlink	inter-protein xl	699	34	665	F1905_sailerc	3385.751	678.158	5.000	-3.5	3	9	0.72	33.09	0.327	sp|P32794|AFG2_YEAST:699:x:sp|P53145|LSG1_YEAST:34
ILTPESQLKK-GNG	sp|P05737|RL7	sp|Q06218|DBP9_YEAST	xlink	inter-protein xl	14,14	559	545	F1905_sailerc	1996.161	666.395	3.000	2.2	2	6	0.90	33.02	0.327	sp|P05737|RL7A_YEAST:14:|:sp|Q12213|RL7B_YEAST:14:x:sp|Q06218|DBP9_YEAST:559
NKACEKLLASR-LL	sp|Q02892|NC	sp|Q07844|RIX7_YEAST	xlink	inter-protein xl	338	30	308	F1905_sailerc	2542.384	636.604	4.000	2.1	1	7	0.88	32.99	0.327	sp|Q02892|NOG1_YEAST:338:x:sp|Q07844|RIX7_YEAST:30
AKYIQNTR-GSQK	sp|P39715|ECM	sp|P0CX45|RL2A_YEAST	xlink	inter-protein xl	135	251	116	F1905_sailerc	1892.962	631.995	3.000	1.3	2	4	0.80	32.99	0.327	sp|P0CX45|RL2A_YEAST:251:x:sp|P39715|ECM1_YEAST:135
KGLVTLETVDSYT	sp|Q03862|AR	sp|P05743|RL26A_YEAST,s	xlink	inter-protein xl	332	84,84	248	F1905_sailerc	2881.564	721.399	4.000	-1.8	1	7	0.67	32.97	0.327	sp|P05743|RL26A_YEAST:84:|:sp|P53221|RL26B_YEAST:84:x:sp|Q03862|ARX1_YEAST:332
VVIVKPHDEGSK-I	sp|P0C2H6|RL2	sp|P0CX82|RL19A_YEAST	xlink	inter-protein xl	27,27	186	159	F1905_sailerc	2318.237	580.567	4.000	0.0	1	6	0.77	32.96	0.327	sp|P0C2H6|RL27A_YEAST:27:|:sp|P0C2H7|RL27B_YEAST:27:x:sp|P0CX82|RL19A_YEAST:186
QINNAKK-KGGKL	sp|P38779|CIC	sp|P05743|RL26A_YEAST,s	xlink	inter-protein xl	354	125,125	229	F1905_sailerc	1582.908	528.644	3.000	2.4	2	8	0.93	32.95	0.327	sp|P05743|RL26A_YEAST:125:|:sp|P53221|RL26B_YEAST:125:x:sp|P38779|CIC1_YEAST:354
LDLAKVAKSYGFP	sp|Q03532|HA	sp|P40991|NOP2_YEAST	xlink	inter-protein xl	475	604	129	F1905_sailerc	2695.556	674.897	4.000	-4.5	1	7	0.91	32.94	0.327	sp|P40991|NOP2_YEAST:604:x:sp|Q03532|HAS1_YEAST:475
MFPAMMKLASTI	sp|Q99257|ME	sp|Q08287|NOP8_YEAST	xlink	inter-protein xl	154	471	317	F1905_sailerc	2619.346	655.844	4.000	1.9	1	11	0.93	32.90	0.327	sp|Q08287|NOP8_YEAST:471:x:sp|Q99257|MEX67_YEAST:154
SELEYYAMLSKTK-	sp|P14120|RL3	sp|P0C2H6|RL27A_YEAST,s	xlink	inter-protein xl	64	9,9	55	F1905_sailerc	2597.410	866.811	3.000	-1.6	4	8	0.59	32.89	0.327	sp|P0C2H6|RL27A_YEAST:9:|:sp|P0C2H7|RL27B_YEAST:9:x:sp|P14120|RL30_YEAST:64
KSSASSNSADAK-I	sp|P32794|AF(	sp|Q07915|RLP24_YEAST	xlink	inter-protein xl	13	184	171	F1905_sailerc	2031.125	678.049	3.000	1.9	3	4	0.57	32.77	0.327	sp|P32794|AFG2_YEAST:13:x:sp|Q07915|RLP24_YEAST:184
KSSASSNSADAK-I	sp|P32794|AF(	sp|Q02892|NOG1_YEAST	xlink	inter-protein xl	13	607	594	F1905_sailerc	2053.980	514.503	4.000	1.5	3	6	0.69	32.69	0.327	sp|P32794|AFG2_YEAST:13:x:sp|Q02892|NOG1_YEAST:607

intra-protein crosslinks

Crosslinked pept	Protein1	Protein2	Type	XLType	Position 1	Position 2	deltaAA	Annotation	Mr	Mz	z	Error_rel[ppn	nseen	mions	deltaS	Id-Score	FDR	uxID
VLIDGPKAGVPR-	sp|P36105|RL1	sp|P36105|RL14A_YEAST,s	xlink	intra/inter xl	50,50	83,83	33	F1905_sailerc	2092.227	524.064	4.000	0.4	7	9	0.77	44.26	0.000	sp|P36105|RL14A_YEAST:50:|:sp|P38754|RL14B_YEAST:50:x:sp|P36105|RL14A_YEAST:83:|:sp|P38754|RL14B_YEAST:83
TGIAIGLNKGK-KV	sp|P05745|RL3	sp|O14455|RL36B_YEAST	xlink	intra/inter xl	13,13	16	3	F1905_sailerc	2308.320	578.088	4.000	0.7	3	11	0.80	43.54	0.000	sp|O14455|RL36B_YEAST:13:|:sp|P05745|RL36A_YEAST:13:x:sp|O14455|RL36B_YEAST:16
AEAKAQGVAVQL	sp|Q02753|RL2	sp|Q02753|RL21A_YEAST,s	xlink	intra/inter xl	120,120	115,115	5	F1905_sailerc	2079.183	520.804	4.000	1.6	3	7	0.62	43.20	0.000	sp|Q02753|RL21A_YEAST:115:|:sp|Q12672|RL21B_YEAST:115:x:sp|Q02753|RL21A_YEAST:120:|:sp|Q12672|RL21B_YEAST:120
GMVSHKTAR-GK	sp|P26784|RL1	sp|P26784|RL16A_YEAST,s	xlink	intra/inter xl	91,90	96,95	5	F1905_sailerc	1867.009	467.760	4.000	-0.6	1	9	0.82	42.12	0.000	sp|P26784|RL16A_YEAST:91:|:sp|P26785|RL16B_YEAST:90:x:sp|P26784|RL16A_YEAST:96:|:sp|P26785|RL16B_YEAST:95
SAGVQGSKVSK-C	sp|P24000|RL2	sp|P04449|RL24A_YEAST,s	xlink	intra/inter xl	137	144,144	7	F1905_sailerc	2189.184	548.304	4.000	1.5	2	7	0.85	39.66	0.022	sp|P04449|RL24A_YEAST:144:|:sp|P24000|RL24B_YEAST:144:x:sp|P24000|RL24B_YEAST:137
GAFQKVAATSR-F	sp|P04449|RL2	sp|P04449|RL24A_YEAST	xlink	intra/inter xl	149,149	140	9	F1905_sailerc	2108.141	703.722	3.000	1.6	2	5	0.94	38.38	0.022	sp|P04449|RL24A_YEAST:140:x:sp|P04449|RL24A_YEAST:149:|:sp|P24000|RL24B_YEAST:149
QQAKGAFQK-KAI	sp|P04449|RL2	sp|P04449|RL24A_YEAST,s	xlink	intra/inter xl	144,144	127,127	17	F1905_sailerc	1816.022	455.013	4.000	0.8	1	7	0.91	38.12	0.022	sp|P04449|RL24A_YEAST:127:|:sp|P24000|RL24B_YEAST:127:x:sp|P04449|RL24A_YEAST:144:|:sp|P24000|RL24B_YEAST:144
GAFQKVAATSR-A	sp|P04449|RL2	sp|P04449|RL24A_YEAST,s	xlink	intra/inter xl	149,149	120,120	29	F1905_sailerc	1917.044	480.269	4.000	0.5	2	4	0.88	37.65	0.022	sp|P04449|RL24A_YEAST:120:|:sp|P24000|RL24B_YEAST:120:x:sp|P04449|RL24A_YEAST:149:|:sp|P24000|RL24B_YEAST:149
GAFQKVAATSR-A	sp|P04449|RL2	sp|P24000|RL24B_YEAST	xlink	intra/inter xl	149,149	129	20	F1905_sailerc	2204.197	735.740	3.000	2.8	3	7	0.90	36.95	0.022	sp|P04449|RL24A_YEAST:149:|:sp|P24000|RL24B_YEAST:149:x:sp|P24000|RL24B_YEAST:129
AKNPLTHSTPK-S	sp|P29453|RL8	sp|P17076|RL8A_YEAST,s	xlink	intra/inter xl	23	18,18	5	F1905_sailerc	1994.080	399.824	5.000	0.2	5	6	0.87	36.78	0.022	sp|P17076|RL8A_YEAST:18:|:sp|P29453|RL8B_YEAST:18:x:sp|P29453|RL8B_YEAST:23
VFAAEKLGSK-NK	sp|P10664|RL4	sp|P10664|RL4A_YEAST,s	xlink	intra/inter xl	338,338	321,321	17	F1905_sailerc	2056.206	515.059	4.000	0.9	4	9	0.82	35.98	0.022	sp|P10664|RL4A_YEAST:321:|:sp|P49626|RL4B_YEAST:321:x:sp|P10664|RL4A_YEAST:338:|:sp|P49626|RL4B_YEAST:338
GAFQKVAATSR-A	sp|P04449|RL2	sp|P04449|RL24A_YEAST	xlink	intra/inter xl	149,149	129	20	F1905_sailerc	2236.184	560.054	4.000	1.4	3	5	0.85	35.59	0.022	sp|P04449|RL24A_YEAST:129:x:sp|P04449|RL24A_YEAST:149:|:sp|P24000|RL24B_YEAST:149
AQRPITGASLDLI	sp|P04449|RL2	sp|P04449|RL24A_YEAST,s	xlink	intra/inter xl	91,91	97,97	6	F1905_sailerc	2732.559	547.520	5.000	1.7	2	9	0.72	34.93	0.022	sp|P04449|RL24A_YEAST:91:|:sp|P24000|RL24B_YEAST:91:x:sp|P04449|RL24A_YEAST:97:|:sp|P24000|RL24B_YEAST:97
KALIQR-KGGKLE	sp|P05743|RL2	sp|P05743|RL26A_YEAST,s	xlink	intra/inter xl	116,116	125,125	9	F1905_sailerc	1495.910	499.645	3.000	1.0	2	7	0.89	34.81	0.022	sp|P05743|RL26A_YEAST:116:|:sp|P53221|RL26B_YEAST:116:x:sp|P05743|RL26A_YEAST:125:|:sp|P53221|RL26B_YEAST:125
VLLSAPLSKELR-K	sp|P05743|RL2	sp|P05743|RL26A_YEAST,s	xlink	intra/inter xl	37,37	125,125	88	F1905_sailerc	2093.247	524.320	4.000	0.7	1	3	0.77	34.62	0.022	sp|P05743|RL26A_YEAST:125:|:sp|P53221|RL26B_YEAST:125:x:sp|P05743|RL26A_YEAST:37:|:sp|P53221|RL26B_YEAST:37
AQQKTAEQIAAE	sp|Q12213|RL	sp|P05737|RL7A_YEAST,s	xlink	intra/inter xl	21	14,14	7	F1905_sailerc	2736.502	685.133	4.000	0.1	3	7	0.92	32.24	0.028	sp|P05737|RL7A_YEAST:14:|:sp|Q12213|RL7B_YEAST:14:x:sp|Q12213|RL7B_YEAST:21
HVLDSEAIKYIASK	sp|P32794|AF-		intralink	intralink	443	448	5	F1905_sailerc	3225.659	807.423	4.000	3.1	10	32	0.51	48.79	0.000	sp|P32794|AFG2_YEAST:443:x:sp|P32794|AFG2_YEAST:448
FLKAGKVAVVVR-	sp|P0C2H6|RL-		intralink	intralink	6,6	9,9	3	F1905_sailerc	1423.893	475.639	3.000	1.2	4	17	0.43	47.48	0.000	sp|P0C2H6|RL27A_YEAST:6:|:sp|P0C2H7|RL27B_YEAST:6:x:sp|P0C2H6|RL27A_YEAST:9:|:sp|P0C2H7|RL27B_YEAST:9
IKAGKITIDSPYLV	sp|P33201|MR-		intralink	intralink	176	179	3	F1905_sailerc	2359.238	787.421	3.000	1.7	4	24	0.37	46.81	0.000	sp|P33201|MRT4_YEAST:176:x:sp|P33201|MRT4_YEAST:179
ETQPMKQKSVET	sp|Q03862|AR-		intralink	intralink	576	578	2	F1905_sailerc	2575.219	859.414	3.000	1.7	5	24	0.25	46.56	0.000	sp|Q03862|ARX1_YEAST:576:x:sp|Q03862|ARX1_YEAST:578
TKMKEMIQLPLE	sp|P32794|AF-		intralink	intralink	527	529	2	F1905_sailerc	2360.217	787.747	3.000	2.2	4	24	0.40	46.21	0.000	sp|P32794|AFG2_YEAST:527:x:sp|P32794|AFG2_YEAST:529
VYWSDIGGQEEL	sp|P32794|AF-		intralink	intralink	525	527	2	F1905_sailerc	2149.072	717.365	3.000	-2.0	5	24	0.35	45.46	0.000	sp|P32794|AFG2_YEAST:525:x:sp|P32794|AFG2_YEAST:527
DLMDTLYEKNHY	sp|Q02892|NC-		intralink	intralink	88	92	4	F1905_sailerc	2618.346	655.594	4.000	1.6	11	29	0.70	44.27	0.000	sp|Q02892|NOG1_YEAST:88:x:sp|Q02892|NOG1_YEAST:92
EHVKELLKYSNET	sp|P0CX43|RL-		intralink	intralink	14	18	4	F1905_sailerc	1854.979	619.334	3.000	3.4	2	18	0.55	43.02	0.000	sp|P0CX43|RL1A_YEAST:14:x:sp|P0CX43|RL1A_YEAST:18
DFAQTHILKLKTS	sp|Q03862|AR-		intralink	intralink	371	373	2	F1905_sailerc	1795.004	599.342	3.000	2.5	4	19	0.80	42.62	0.000	sp|Q03862|ARX1_YEAST:371:x:sp|Q03862|ARX1_YEAST:373
YGLNHVVSLIENK	sp|P29453|RL8-		intralink	intralink	146	147	1	F1905_sailerc	1950.097	651.040	3.000	1.8	2	21	0.42	42.35	0.000	sp|P29453|RL8B_YEAST:146:x:sp|P29453|RL8B_YEAST:147
LPAASLGDMVM	sp|P0CX41|RL-		intralink	intralink	63	66	3	F1905_sailerc	2449.346	613.344	4.000	0.8	1	28	0.54	42.26	0.000	sp|P0CX41|RL23A_YEAST:63:x:sp|P0CX41|RL23A_YEAST:66
LALVIKSGKYTLGY	sp|P14120|RL-		intralink	intralink	19	22	3	F1905_sailerc	1791.056	598.027	3.000	0.9	3	18	0.57	42.12	0.000	sp|P14120|RL30_YEAST:19:x:sp|P14120|RL30_YEAST:22
SSVNSKSGEIKSV	sp|P47019|ALE-		intralink	intralink	46	51	5	F1905_sailerc	2551.323	851.449	3.000	2.2	2	22	0.41	42.00	0.000	sp|P47019|ALB1_YEAST:46:x:sp|P47019|ALB1_YEAST:51
VSAAAASTSKSAE	sp|P38344|REI-		intralink	intralink	63	67	4	F1905_sailerc	2127.147	710.057	3.000	2.1	1	22	0.00	41.76	0.000	sp|P38344|REI1_YEAST:63:x:sp|P38344|REI1_YEAST:67
KRPVPKGATYGK	sp|P05748|RL-		intralink	intralink	72,72	77,77	5	F1905_sailerc	2519.388	630.855	4.000	0.7	7	19	0.60	41.59	0.000	sp|P05748|RL15A_YEAST:72:|:sp|P54780|RL15B_YEAST:72:x:sp|P05748|RL15A_YEAST:77:|:sp|P54780|RL15B_YEAST:77
SKAQQKTAEQVA	sp|P05737|RL-		intralink	intralink	17	21	4	F1905_sailerc	1781.930	594.984	3.000	1.4	2	24	0.50	41.45	0.000	sp|P05737|RL7A_YEAST:17:x:sp|P05737|RL7A_YEAST:21
KTVKAQRPITGAS	sp|P04449|RL-		intralink	intralink	74,74	77,77	3	F1905_sailerc	2076.233	693.085	3.000	0.9	4	22	0.48	41.30	0.000	sp|P04449|RL24A_YEAST:74:|:sp|P24000|RL24B_YEAST:74:x:sp|P04449|RL24A_YEAST:77:|:sp|P24000|RL24B_YEAST:77
VVIVKPHDEGSKS	sp|P0C2H6|RL-		intralink	intralink	27,27	34,34	7	F1905_sailerc	2916.565	973.196	3.000	1.3	5	24	0.39	40.95	0.000	sp|P0C2H6|RL27A_YEAST:27:|:sp|P0C2H7|RL27B_YEAST:27:x:sp|P0C2H6|RL27A_YEAST:34:|:sp|P0C2H7|RL27B_YEAST:34
ALATESGINFLAV	sp|P32794|AF-		intralink	intralink	582	589	7	F1905_sailerc	3176.649	1059.891	3.000	3.2	14	25	0.33	40.95	0.000	sp|P32794|AFG2_YEAST:582:x:sp|P32794|AFG2_YEAST:589
GLGTDANIDKFSL	sp|P32794|AF-		intralink	intralink	481	485	4	F1905_sailerc	2057.144	686.722	3.000	1.5	29	19	0.62	40.48	0.000	sp|P32794|AFG2_YEAST:481:x:sp|P32794|AFG2_YEAST:485
MAKEHKDIDASLI	sp|P38861|NM-		intralink	intralink	419	422	3	F1905_sailerc	2130.008	711.010	3.000	1.3	3	26	0.32	40.16	0.000	sp|P38861|NMD3_YEAST:419:x:sp|P38861|NMD3_YEAST:422
AFTKKVASANAT	sp|P26784|RL-		intralink	intralink	176	177	1	F1905_sailerc	2117.102	706.709	3.000	0.8	4	22	0.46	40.06	0.000	sp|P26784|RL16A_YEAST:176:x:sp|P26784|RL16A_YEAST:177
KGQSAGKLAAIVE	sp|P36105|RL-		intralink	intralink	25,25	31,31	6	F1905_sailerc	2006.145	669.723	3.000	1.7	2	22	0.85	40.04	0.000	sp|P36105|RL14A_YEAST:25:|:sp|P38754|RL14B_YEAST:25:x:sp|P36105|RL14A_YEAST:31:|:sp|P38754|RL14B_YEAST:31
ANVTHPELGVTVF	sp|P40078|NS-		intralink	intralink	201	202	1	F1905_sailerc	4041.250	1011.320	4.000	3.0	4	26	0.40	39.81	0.000	sp|P40078|NSA2_YEAST:201:x:sp|P40078|NSA2_YEAST:202
KLAKSMGNISQFV	sp|P38861|NM-		intralink	intralink	262	265	3	F1905_sailerc	2048.084	683.702	3.000	2.0	2	19	0.54	39.56	0.000	sp|P38861|NMD3_YEAST:262:x:sp|P38861|NMD3_YEAST:265
EQLASQLVDLKKE	sp|P0CX84|RL-		intralink	intralink	25	26	1	F1905_sailerc	2192.237	731.754	3.000	3.1	3	20	0.66	39.48	0.000	sp|P0CX84|RL35A_YEAST:25:x:sp|P0CX84|RL35A_YEAST:26
TKAQQKTAEQIA	sp|Q12213|RL-		intralink	intralink	17	21	4	F1905_sailerc	1809.960	604.328	3.000	0.9	2	19	0.42	39.35	0.000	sp|Q12213|RL7B_YEAST:17:x:sp|Q12213|RL7B_YEAST:21
GVLLYGPPGCSKT	sp|P32794|AF-		intralink	intralink	563	568	5	F1905_sailerc	3313.806	829.459	4.000	2.0	10	20	0.76	39.34	0.000	sp|P32794|AFG2_YEAST:563:x:sp|P32794|AFG2_YEAST:568
LIDVDGKPQIQVE	sp|P10591|HSI-		intralink	intralink	106	110	4	F1905_sailerc	3812.996	954.257	4.000	1.4	12	27	0.33	39.28	0.000	sp|P10591|HSP71_YEAST:106:x:sp|P10591|HSP71_YEAST:110
LCSGVTGLLFTDE	sp|P33201|MR-		intralink	intralink	113	117	4	F1905_sailerc	3277.617	1093.547	3.000	-1.5	13	23	0.37	39.26	0.000	sp|P33201|MRT4_YEAST:113:x:sp|P33201|MRT4_YEAST:117
TPFIPESVKNLKK-	sp|Q02892|NC-		intralink	intralink	383	386	3	F1905_sailerc	1637.941	546.988	3.000	0.9	3	15	0.56	39.09	0.000	sp|Q02892|NOG1_YEAST:383:x:sp|Q02892|NOG1_YEAST:386
APKYASKAVPHYI	sp|P04456|RL2-		intralink	intralink	45	49	4	F1905_sailerc	1738.924	580.649	3.000	4.9	2	18	0.49	39.05	0.000	sp|P04456|RL25_YEAST:45:x:sp|P04456|RL25_YEAST:49
KKATVDEFPLCVH	sp|P41805|RL-		intralink	intralink	39	40	1	F1905_sailerc	3620.853	906.221	4.000	3.6	6	20	0.49	38.97	0.000	sp|P41805|RL10_YEAST:39:x:sp|P41805|RL10_YEAST:40
LPAASLGDMVM	sp|P0CX41|RL-		intralink	intralink	63	64	1	F1905_sailerc	2449.348	613.345	4.000	1.8	3	28	0.59	38.80	0.000	sp|P0CX41|RL23A_YEAST:63:x:sp|P0CX41|RL23A_YEAST:64
DKNVKHSGNIQL	sp|P0CX53|RL-		intralink	intralink	96	99	3	F1905_sailerc	2429.294	810.773	3.000	1.2	1	20	0.50	38.74	0.000	sp|P0CX53|RL12A_YEAST:96:x:sp|P0CX53|RL12A_YEAST:99
LKEYQSKIIVFPR-I	sp|Q12690|RL-		intralink	intralink	117,117	122,122	5	F1905_sailerc	1758.008	587.010	3.000	-0.1	4	12	0.61	38.66	0.000	sp|P40212|RL13B_YEAST:117:|:sp|Q12690|RL13A_YEAST:117:x:sp|P40212|RL13B_YEAST:122:|:sp|Q12690|RL13A_YEAST:122
GMPHKFYQGKTC	sp|Q02753|RL-		intralink	intralink	55,55	60,60	5	F1905_sailerc	2291.180	764.735	3.000	1.4	1	17	0.45	38.27	0.000	sp|Q02753|RL21A_YEAST:55:|:sp|Q12672|RL21B_YEAST:55:x:sp|Q02753|RL21A_YEAST:60:|:sp|Q12672|RL21B_YEAST:60
LIDVDGKPQIQVE	sp|P10591|HSI-		intralink	intralink	98	110	12	F1905_sailerc	3812.995	954.256	4.000	0.9	4	18	0.45	38.27	0.000	sp|P10591|HSP71_YEAST:110:x:sp|P10591|HSP71_YEAST:98
QGKSKLIIIAANTP	sp|P14120|RL-		intralink	intralink	38	40	2	F1905_sailerc	1959.193	654.072	3.000	2.4	4	19	0.43	37.99	0.000	sp|P14120|RL30_YEAST:38:x:sp|P14120|RL30_YEAST:40
SSASSNSADAKAS	sp|P32794|AF-		intralink	intralink	24	27	3	F1905_sailerc	1722.845	575.290	3.000	1.8	4	19	0.43	37.97	0.000	sp|P32794|AFG2_YEAST:24:x:sp|P32794|AFG2_YEAST:27
KRPVPKGATYGK	sp|P05748|RL-		intralink	intralink	72,72	83,83	11	F1905_sailerc	2519.392	630.856	4.000	2.5	12	18	0.66	37.90	0.000	sp|P05748|RL15A_YEAST:72:|:sp|P54780|RL15B_YEAST:72:x:sp|P05748|RL15A_YEAST:83:|:sp|P54780|RL15B_YEAST:83
HFEKAFKGIAR-K	sp|P32794|AF-		intralink	intralink	752	755	3	F1905_sailerc	1440.789	481.271	3.000	0.9	133	12	0.54	37.51	0.000	sp|P32794|AFG2_YEAST:752:x:sp|P32794|AFG2_YEAST:755
KLATRPKCGDCG	sp|P87262|RL-		intralink	intralink	37,37	43,43	6	F1905_sailerc	2779.468	695.875	4.000	3.5	4	17	0.48	37.44	0.000	sp|P40525|RL34B_YEAST:37:|:sp|P87262|RL34A_YEAST:37:x:sp|P40525|RL34B_YEAST:43:|:sp|P87262|RL34A_YEAST:43
HKKSEELISQDTH	sp|P38861|NM-		intralink	intralink	223	224	1	F1905_sailerc	2397.186	800.070	3.000	2.1	2	18	0.40	37.36	0.000	sp|P38861|NMD3_YEAST:223:x:sp|P38861|NMD3_YEAST:224
SLVDNMITGVTKC	sp|P05738|RL-		intralink	intralink	84,84	87,87	3	F1905_sailerc	2054.076	685.700	3.000	0.3	3	21	0.41	37.22	0.000	sp|P05738|RL9A_YEAST:84:|:sp|P51401|RL9B_YEAST:84:x:sp|P05738|RL9A_YEAST:87:|:sp|P51401|RL9B_YEAST:87
VDSVAHPAPHDK	sp|Q08208|NC-		intralink	intralink	277	278	1	F1905_sailerc	1693.891	565.638	3.000	0.4	1	19	0.67	37.06	0.000	sp|Q08208|NOP12_YEAST:277:x:sp|Q08208|NOP12_YEAST:278
KAEKTGTKPAAVF	sp|P10664|RL4-		intralink	intralink	343	346	3	F1905_sailerc	2057.144	686.723	3.000	1.8	2	18	0.40	36.98	0.000	sp|P10664|RL4A_YEAST:343:x:sp|P10664|RL4A_YEAST:346
TKEAVAALKAVG	sp|P10664|RL4-		intralink	intralink	163,163	170,170	7	F1905_sailerc	2059.172	687.399	3.000	2.1	2	19	0.68	36.81	0.000	sp|P10664|RL4A_YEAST:163:|:sp|P49626|RL4B_YEAST:163:x:sp|P10664|RL4A_YEAST:170:|:sp|P49626|RL4B_YEAST:170
FGQSLFQCKQLKR	sp|Q02892|NC-		intralink	intralink	126	129	3	F1905_sailerc	1776.935	593.319	3.000	0.2	2	16	0.65	36.69	0.000	sp|Q02892|NOG1_YEAST:126:x:sp|Q02892|NOG1_YEAST:129
QVKKLEIQQHAR-	sp|P0CX25|RL4-		intralink	intralink	27	28	1	F1905_sailerc	1614.920	539.314	3.000	-0.5	4	18	0.76	36.50	0.000	sp|P0CX25|RL43A_YEAST:27:x:sp|P0CX25|RL43A_YEAST:28
DLNQLTFSAHTG	sp|P53145|LSG-		intralink	intralink	570	573	3	F1905_sailerc	2964.463	989.162	3.000	1.6	2	25	0.32	36.09	0.000	sp|P53145|LSG1_YEAST:570:x:sp|P53145|LSG1_YEAST:573
LSHLSSNFPFVHE	sp|Q03862|AR-		intralink	intralink	413	414	1	F1905_sailerc	3205.639	802.418	4.000	2.9	3	18	0.48	36.07	0.000	sp|Q03862|ARX1_YEAST:413:x:sp|Q03862|ARX1_YEAST:414
YGLNHVVALIENK	sp|P17076|RL8-		intralink	intralink	146	147	1	F1905_sailerc	1934.101	645.708	3.000	1.2	2	17	0.44	36.00	0.000	sp|P17076|RL8A_YEAST:146:x:sp|P17076|RL8A_YEAST:147
VVLIKKGQSAGK-I	sp|P36105|RL-		intralink	intralink	24,24	25,25	1	F1905_sailerc	1364.840	455.954	3.000	0.5	2	17	0.35	35.97	0.000	sp|P36105|RL14A_YEAST:24:|:sp|P38754|RL14B_YEAST:24:x:sp|P36105|RL14A_YEAST:25:|:sp|P38754|RL14B_YEAST:25
QLLTKIDKQGVYP	sp|Q03862|AR-		intralink	intralink	381	384	3	F1905_sailerc	1915.084	639.369	3.000	1.0	4	17	0.59	35.73	0.000	sp|Q03862|ARX1_YEAST:381:x:sp|Q03862|ARX1_YEAST:384
SVALDLYFQNKKN	sp|P47019|ALE-		intralink	intralink	62	63	1	F1905_sailerc	4008.015	1337.013	3.000	1.7	6	26	0.69	35.00	0.000	sp|P47019|ALB1_YEAST:62:x:sp|P47019|ALB1_YEAST:63
AKNPLTHSTPKNF	sp|P29453|RL8-		intralink	intralink	23	32	9	F1905_sailerc	2470.340	824.455	3.000	2.9	4	15	0.68	34.70	0.000	sp|P29453|RL8B_YEAST:23:x:sp|P29453|RL8B_YEAST:32
VTKATLELLKLIEP	sp|P05737|RL7-		intralink	intralink	121,121	128,128	7	F1905_sailerc	3225.759	807.448	4.000	0.5	4	18	0.46	34.52	0.000	sp|P05737|RL7A_YEAST:121:|:sp|Q12213|RL7B_YEAST:121:x:sp|P05737|RL7A_YEAST:128:|:sp|Q12213|RL7B_YEAST:128
NTAAETFKLFNKY	sp|P17076|RL8-		intralink	intralink	92,92	96,96	4	F1905_sailerc	2566.311	642.586	4.000	1.7	3	15	0.68	34.21	0.000	sp|P17076|RL8A_YEAST:92:|:sp|P29453|RL8B_YEAST:92:x:sp|P17076|RL8A_YEAST:96:|:sp|P29453|RL8B_YEAST:96
KILAHLDDPDSNK	sp|P34232|MT-		intralink	intralink	25	37	12	F1905_sailerc	3304.670	827.175	4.000	1.0	5	16	0.46	34.01	0.000	sp|P34232|MTR2_YEAST:25:x:sp|P34232|MTR2_YEAST:37
ASKFKLPAEFITRP	sp|P32794|AF-		intralink	intralink	27	29	2	F1905_sailerc	2191.217	731.414	3.000	0.9	9	19	0.57	33.85	0.000	sp|P32794|AFG2_YEAST:27:x:sp|P32794|AFG2_YEAST:29
SAGVQGSKVSKQ	sp|P24000|RL2-		intralink	intralink	137	140	3	F1905_sailerc	1639.890	547.638	3.000	0.5	2	15	0.74	33.83	0.000	sp|P24000|RL24B_YEAST:137:x:sp|P24000|RL24B_YEAST:140
IGPLGLSPKKVGE	sp|P0CX53|RL-		intralink	intralink	40	41	1	F1905_sailerc	1859.080	620.701	3.000	1.5	3	19	0.67	33.81	0.000	sp|P0CX53|RL12A_YEAST:40:x:sp|P0CX53|RL12A_YEAST:41
EAAAIAEGKSKQD	sp|P29453|RL8-		intralink	intralink	120	133	13	F1905_sailerc	2396.262	799.762	3.000	1.5	6	14	0.00	33.45	0.000	sp|P29453|RL8B_YEAST:120:x:sp|P29453|RL8B_YEAST:133
IPKVQKLVTDYFN	sp|P10591|HSI-		intralink	intralink	342	345	3	F1905_sailerc	1887.054	630.026	3.000	1.7	2	13	0.49	33.35	0.000	sp|P10591|HSP71_YEAST:342:x:sp|P10591|HSP71_YEAST:345
ELAELKVQKLSRP	sp|P0CX84|RL-		intralink	intralink	32	35	3	F1905_sailerc	2073.220	692.081	3.000	0.1	4	19	0.69	32.89	0.000	sp|P0CX84|RL35A_YEAST:32:x:sp|P0CX84|RL35A_YEAST:35
LLDDSGVIMPGM	sp|P32794|AF-		intralink	intralink	168	170	2	F1905_sailerc	2357.242	786.755	3.000	2.0	3	17	0.47	32.87	0.000	sp|P32794|AFG2_YEAST:168:x:sp|P32794|AFG2_YEAST:170
TLTAKALATESGIN	sp|P32794|AF-		intralink	intralink	568	582	14	F1905_sailerc	2870.588	718.655	4.000	2.9	14	15	0.63	32.85	0.000	sp|P32794|AFG2_YEAST:568:x:sp|P32794|AFG2_YEAST:582
LNPYAKVFAAEKL	sp|P10664|RL4-		intralink	intralink	332,332	338,338	6	F1905_sailerc	1873.035	625.353	3.000	-0.1	1	14	0.79	32.82	0.000	sp|P10664|RL4A_YEAST:332:|:sp|P49626|RL4B_YEAST:332:x:sp|P10664|RL4A_YEAST:338:|:sp|P49626|RL4B_YEAST:338
LPAEFITRPHPSKD	sp|P32794|AF-		intralink	intralink	42	46	4	F1905_sailerc	5042.517	1261.637	4.000	2.1	5	22	0.71	32.78	0.000	sp|P32794|AFG2_YEAST:42:x:sp|P32794|AFG2_YEAST:46
LEQYKKNKVNER-	sp|Q03862|AR-		intralink	intralink	81	83	2	F1905_sailerc	1720.880	574.635	3.000	1.0	2	12	0.75	32.68	0.000	sp|Q03862|ARX1_YEAST:81:x:sp|Q03862|ARX1_YEAST:83
INSGTFVKVTKATI	sp|P05737|RL7-		intralink	intralink	118,118	121,121	3	F1905_sailerc	2099.227	700.750	3.000	1.1	2	16	0.82	32.32	0.000	sp|P05737|RL7A_YEAST:118:|:sp|Q12213|RL7B_YEAST:118:x:sp|P05737|RL7A_YEAST:121:|:sp|Q12213|RL7B_YEAST:121
LWDPKSGQCLGD	sp|P25382|NLE	sp|P25382|NLE1_YEAST	xlink	intra-protein xl	216	345	129	F1905_sailerc	2966.483	742.629	4.000	0.7	2	13	0.71	46.51	0.000	sp|P25382|NLE1_YEAST:216:x:sp|P25382|NLE1_YEAST:345
IVKAGGECITLDQI	sp|P0CX49|RL1	sp|P0CX49|RL18A_YEAST	xlink	intra-protein xl	116	98	18	F1905_sailerc	3471.921	868.988	4.000	1.2	3	15	0.60	45.09	0.000	sp|P0CX49|RL18A_YEAST:116:x:sp|P0CX49|RL18A_YEAST:98
AEKTDLTNILIR-T	sp|P39715|ECM	sp|P39715|ECM1_YEAST	xlink	intra-protein xl	57	70	13	F1905_sailerc	2781.554	696.396	4.000	2.0	2	12	0.66	44.36	0.000	sp|P39715|ECM1_YEAST:57:x:sp|P39715|ECM1_YEAST:70
KLLEIAR-VMQEK	sp|P38344|REI	sp|P38344|REI1_YEAST	xlink	intra-protein xl	90	152	62	F1905_sailerc	1882.109	471.535	4.000	0.7	2	10	0.80	44.05	0.000	sp|P38344|REI1_YEAST:152:x:sp|P38344|REI1_YEAST:90
GSQKTQD-LPSGA	sp|P0CX45|RL2	sp|P0CX45|RL2A_YEAST	xlink	intra-protein xl	251	155	96	F1905_sailerc	1599.849	534.291	3.000	1.2	3	10	0.77	43.95	0.000	sp|P0CX45|RL2A_YEAST:155:x:sp|P0CX45|RL2A_YEAST:251
LVKALNFTNR-AK	sp|P39715|ECM	sp|P39715|ECM1_YEAST	xlink	intra-protein xl	115	135	20	F1905_sailerc	2305.296	577.332	4.000	2.4	4	12	0.74	43.26	0.000	sp|P39715|ECM1_YEAST:115:x:sp|P39715|ECM1_YEAST:135
LVKALNFTNR-IAK	sp|P39715|ECM	sp|P39715|ECM1_YEAST	xlink	intra-protein xl	115	129	14	F1905_sailerc	2086.232	522.566	4.000	2.6	2	9	0.71	43.08	0.000	sp|P39715|ECM1_YEAST:115:x:sp|P39715|ECM1_YEAST:129
EKALNEEAEAR-D	sp|P0CX82|RL1	sp|P0CX82|RL19A_YEAST	xlink	intra-protein xl	153	186	33	F1905_sailerc	2270.127	757.717	3.000	-0.3	4	10	0.55	43.05	0.000	sp|P0CX82|RL19A_YEAST:153:x:sp|P0CX82|RL19A_YEAST:186
GSSSLYTLVINDA	sp|P49167|RL3	sp|P49167|RL38_YEAST	xlink	intra-protein xl	61	9	52	F1905_sailerc	3465.865	867.474	4.000	1.2	4	12	0.53	42.48	0.000	sp|P49167|RL38_YEAST:61:x:sp|P49167|RL38_YEAST:9
KVMPAIVVR-GKP	sp|P0CX41|RL2	sp|P0CX41|RL23A_YEAST	xlink	intra-protein xl	72	66	6	F1905_sailerc	1848.103	463.034	4.000	0.8	7	7	0.67	42.01	0.000	sp|P0CX41|RL23A_YEAST:66:x:sp|P0CX41|RL23A_YEAST:72
AGLLKPAR-NLKY	sp|P47019|ALE	sp|P47019|ALB1_YEAST	xlink	intra-protein xl	37	102	65	F1905_sailerc	1955.135	489.792	4.000	2.0	1	9	0.74	41.96	0.000	sp|P47019|ALB1_YEAST:102:x:sp|P47019|ALB1_YEAST:37
SSASSNSADAKAS	sp|P32794|AF(	sp|P32794|AFG2_YEAST	xlink	intra-protein xl	24	13	11	F1905_sailerc	2599.226	650.814	4.000	1.8	2	17	0.76	41.42	0.022	sp|P32794|AFG2_YEAST:13:x:sp|P32794|AFG2_YEAST:24
AKALGIK-VTNPK	sp|P38061|RL3	sp|P38061|RL32_YEAST	xlink	intra-protein xl	113	123	10	F1905_sailerc	1607.931	403.001	4.000	0.9	4	9	0.87	41.30	0.022	sp|P38061|RL32_YEAST:113:x:sp|P38061|RL32_YEAST:123
KVSSASAAASESD	sp|P26785|RL1	sp|P26785|RL16B_YEAST	xlink	intra-protein xl	176	169	7	F1905_sailerc	2603.300	651.833	4.000	1.2	9	7	0.66	41.01	0.022	sp|P26785|RL16B_YEAST:169:x:sp|P26785|RL16B_YEAST:176
INNVLNKIHVAQP	sp|Q02892|NC	sp|Q02892|NOG1_YEAST	xlink	intra-protein xl	360	349	11	F1905_sailerc	2669.512	534.910	5.000	1.0	7	8	0.85	40.98	0.022	sp|Q02892|NOG1_YEAST:349:x:sp|Q02892|NOG1_YEAST:360
KLLVDASAK-NLK	sp|P47019|ALE	sp|P47019|ALB1_YEAST	xlink	intra-protein xl	108	102	6	F1905_sailerc	2074.187	519.555	4.000	4.1	4	10	0.77	40.69	0.022	sp|P47019|ALB1_YEAST:102:x:sp|P47019|ALB1_YEAST:108
GEMKGSAITGPV	sp|P0CX41|RL2	sp|P0CX41|RL23A_YEAST	xlink	intra-protein xl	110	72	38	F1905_sailerc	2480.389	621.105	4.000	1.4	2	11	0.53	40.48	0.022	sp|P0CX41|RL23A_YEAST:110:x:sp|P0CX41|RL23A_YEAST:72
SSVNSKSGEIK-KL	sp|P47019|ALE	sp|P47019|ALB1_YEAST	xlink	intra-protein xl	46	108	62	F1905_sailerc	2216.230	739.751	3.000	1.6	1	10	0.85	40.37	0.022	sp|P47019|ALB1_YEAST:108:x:sp|P47019|ALB1_YEAST:46
GSSSLYTLVINDA	sp|P49167|RL3	sp|P49167|RL38_YEAST	xlink	intra-protein xl	61	64	3	F1905_sailerc	3097.771	775.451	4.000	2.2	69	13	0.61	40.31	0.022	sp|P49167|RL38_YEAST:61:x:sp|P49167|RL38_YEAST:64
FDILTKQFSR-ESV	sp|P32794|AF(	sp|P32794|AFG2_YEAST	xlink	intra-protein xl	425	467	42	F1905_sailerc	2482.336	621.592	4.000	4.2	2	11	0.62	39.88	0.022	sp|P32794|AFG2_YEAST:425:x:sp|P32794|AFG2_YEAST:467
ACEKLLASR-LKS	sp|Q02892|NC	sp|Q02892|NOG1_YEAST	xlink	intra-protein xl	338	349	11	F1905_sailerc	1902.037	635.020	3.000	0.8	4	7	0.93	39.68	0.022	sp|Q02892|NOG1_YEAST:338:x:sp|Q02892|NOG1_YEAST:349
TLASVTKEILGTAC	sp|P0CX53|RL1	sp|P0CX53|RL12A_YEAST	xlink	intra-protein xl	130	119	11	F1905_sailerc	2836.480	710.128	4.000	1.6	2	4	0.64	39.17	0.022	sp|P0CX53|RL12A_YEAST:119:x:sp|P0CX53|RL12A_YEAST:130
GAVSGQKAGLIA	sp|P0CX45|RL2	sp|P0CX45|RL2A_YEAST	xlink	intra-protein xl	234	155	79	F1905_sailerc	2135.243	534.819	4.000	0.2	1	4	0.95	39.14	0.022	sp|P0CX45|RL2A_YEAST:155:x:sp|P0CX45|RL2A_YEAST:234
VAAKIAALAGQQ-	sp|P26321|RL5	sp|P26321|RL5_YEAST	xlink	intra-protein xl	289	270	19	F1905_sailerc	2330.255	777.759	3.000	2.9	3	10	0.89	39.01	0.022	sp|P26321|RL5_YEAST:270:x:sp|P26321|RL5_YEAST:289
KVGEDIAKATK-EF	sp|P0CX53|RL1	sp|P0CX53|RL12A_YEAST	xlink	intra-protein xl	48	54	6	F1905_sailerc	2017.149	505.295	4.000	1.6	3	7	0.82	38.69	0.022	sp|P0CX53|RL12A_YEAST:48:x:sp|P0CX53|RL12A_YEAST:54
IGPLGLSPKK-ATK	sp|P0CX53|RL1	sp|P0CX53|RL12A_YEAST	xlink	intra-protein xl	40	51	11	F1905_sailerc	1869.102	468.283	4.000	2.6	5	7	0.77	38.66	0.022	sp|P0CX53|RL12A_YEAST:40:x:sp|P0CX53|RL12A_YEAST:51
LLDDSGVIMPGM	sp|P32794|AF(	sp|P32794|AFG2_YEAST	xlink	intra-protein xl	168	481	313	F1905_sailerc	3835.015	959.762	4.000	0.1	2	12	0.78	38.51	0.022	sp|P32794|AFG2_YEAST:168:x:sp|P32794|AFG2_YEAST:481
GLGTDANIDKFSL	sp|P32794|AF(	sp|P32794|AFG2_YEAST	xlink	intra-protein xl	481	467	14	F1905_sailerc	2706.432	677.616	4.000	2.2	6	11	0.64	38.48	0.022	sp|P32794|AFG2_YEAST:467:x:sp|P32794|AFG2_YEAST:481
QKSVETSNGGVE	sp|Q03862|AR	sp|Q03862|ARX1_YEAST	xlink	intra-protein xl	578	83	495	F1905_sailerc	2619.284	874.102	3.000	1.1	9	4	0.41	38.16	0.022	sp|Q03862|ARX1_YEAST:578:x:sp|Q03862|ARX1_YEAST:83
VSAAAASTSKSAE	sp|P38344|REI	sp|P38344|REI1_YEAST	xlink	intra-protein xl	63	90	27	F1905_sailerc	2286.280	572.578	4.000	0.1	2	7	0.82	38.03	0.022	sp|P38344|REI1_YEAST:63:x:sp|P38344|REI1_YEAST:90
KLLVDASAK-AGLI	sp|P47019|ALE	sp|P47019|ALB1_YEAST	xlink	intra-protein xl	108	37	71	F1905_sailerc	1906.164	477.549	4.000	1.2	4	10	0.82	37.89	0.022	sp|P47019|ALB1_YEAST:108:x:sp|P47019|ALB1_YEAST:37
FDILTKQFSR-AFK	sp|P32794|AF(	sp|P32794|AFG2_YEAST	xlink	intra-protein xl	425	755	330	F1905_sailerc	2153.202	539.308	4.000	0.9	1	6	0.87	37.75	0.022	sp|P32794|AFG2_YEAST:425:x:sp|P32794|AFG2_YEAST:755
AKSAGVQGSK-VS	sp|P24000|RL2	sp|P24000|RL24B_YEAST	xlink	intra-protein xl	129	140	11	F1905_sailerc	1857.033	465.266	4.000	0.4	3	5	0.95	37.74	0.022	sp|P24000|RL24B_YEAST:129:x:sp|P24000|RL24B_YEAST:140
SSVNSKSGEIK-AG	sp|P47019|ALE	sp|P47019|ALB1_YEAST	xlink	intra-protein xl	46	37	9	F1905_sailerc	2097.180	700.068	3.000	0.5	5	10	0.88	37.42	0.022	sp|P47019|ALB1_YEAST:37:x:sp|P47019|ALB1_YEAST:46
KVMPAIVVR-KGK	sp|P0CX41|RL2	sp|P0CX41|RL23A_YEAST	xlink	intra-protein xl	72	64	8	F1905_sailerc	1976.202	495.058	4.000	2.4	2	9	0.93	37.25	0.022	sp|P0CX41|RL23A_YEAST:64:x:sp|P0CX41|RL23A_YEAST:72
LLGPQLSKAGK-L	sp|P0CX43|RL1	sp|P0CX43|RL1A_YEAST	xlink	intra-protein xl	130	95	35	F1905_sailerc	1992.211	399.450	5.000	0.7	3	5	0.92	37.15	0.022	sp|P0CX43|RL1A_YEAST:130:x:sp|P0CX43|RL1A_YEAST:95
KNESQNSTAVTLC	sp|P47019|ALE	sp|P47019|ALB1_YEAST	xlink	intra-protein xl	63	51	12	F1905_sailerc	4540.299	1136.083	4.000	1.2	3	12	0.37	37.06	0.022	sp|P47019|ALB1_YEAST:51:x:sp|P47019|ALB1_YEAST:63
MKEMIQLPLEAS	sp|P32794|AF(	sp|P32794|AFG2_YEAST	xlink	intra-protein xl	529	755	226	F1905_sailerc	2892.536	724.142	4.000	4.0	3	6	0.54	36.70	0.022	sp|P32794|AFG2_YEAST:529:x:sp|P32794|AFG2_YEAST:755
MKEMIQLPLEAS	sp|P32794|AF(	sp|P32794|AFG2_YEAST	xlink	intra-protein xl	529	525	4	F1905_sailerc	3882.946	971.744	4.000	0.9	23	12	0.82	36.69	0.022	sp|P32794|AFG2_YEAST:525:x:sp|P32794|AFG2_YEAST:529
AEKTDLTNILIR-A	sp|P39715|ECM	sp|P39715|ECM1_YEAST	xlink	intra-protein xl	57	135	78	F1905_sailerc	2516.398	630.107	4.000	0.8	1	8	0.80	36.62	0.022	sp|P39715|ECM1_YEAST:135:x:sp|P39715|ECM1_YEAST:57
EITDIKQFLELTR-K	sp|P49167|RL3	sp|P49167|RL38_YEAST	xlink	intra-protein xl	9	64	55	F1905_sailerc	2979.730	745.940	4.000	1.5	26	13	0.66	36.62	0.022	sp|P49167|RL38_YEAST:64:x:sp|P49167|RL38_YEAST:9
SSASSNSADAKAS	sp|P32794|AF(	sp|P32794|AFG2_YEAST	xlink	intra-protein xl	24	12	12	F1905_sailerc	2727.319	682.838	4.000	1.1	1	9	0.74	36.48	0.022	sp|P32794|AFG2_YEAST:12:x:sp|P32794|AFG2_YEAST:24
TPFIPESVKNLK-K	sp|Q02892|NC	sp|Q02892|NOG1_YEAST	xlink	intra-protein xl	383	387	4	F1905_sailerc	2642.366	661.599	4.000	2.5	4	7	0.85	36.40	0.022	sp|Q02892|NOG1_YEAST:383:x:sp|Q02892|NOG1_YEAST:387
SSASSNSADAKAS	sp|P32794|AF(	sp|P32794|AFG2_YEAST	xlink	intra-protein xl	24	13	11	F1905_sailerc	2727.319	546.472	5.000	1.0	1	10	0.94	36.33	0.022	sp|P32794|AFG2_YEAST:13:x:sp|P32794|AFG2_YEAST:24
IAWAKYFISK-ADL	sp|P53145|LSC	sp|P53145|LSG1_YEAST	xlink	intra-protein xl	251	243	8	F1905_sailerc	2151.239	718.088	3.000	2.3	2	7	0.91	36.30	0.022	sp|P53145|LSG1_YEAST:243:x:sp|P53145|LSG1_YEAST:251
QKSVETSNGGVE	sp|Q03862|AR	sp|Q03862|ARX1_YEAST	xlink	intra-protein xl	578	570	8	F1905_sailerc	3264.667	817.174	4.000	1.4	2	7	0.48	36.04	0.022	sp|Q03862|ARX1_YEAST:570:x:sp|Q03862|ARX1_YEAST:578
LGISAPKGVLLYG	sp|P32794|AF(	sp|P32794|AFG2_YEAST	xlink	intra-protein xl	551	568	17	F1905_sailerc	3998.224	1000.564	4.000	1.7	2	12	0.40	36.03	0.022	sp|P32794|AFG2_YEAST:551:x:sp|P32794|AFG2_YEAST:568
QSDALEPEVKDLS	sp|P39715|ECM	sp|P39715|ECM1_YEAST	xlink	intra-protein xl	47	57	10	F1905_sailerc	3448.833	863.216	4.000	0.9	5	9	0.69	35.97	0.022	sp|P39715|ECM1_YEAST:47:x:sp|P39715|ECM1_YEAST:57
VRGSSSLYTLVIN	sp|P49167|RL3	sp|P49167|RL38_YEAST	xlink	intra-protein xl	61	64	3	F1905_sailerc	3352.938	671.595	5.000	1.2	4	9	0.82	35.89	0.022	sp|P49167|RL38_YEAST:61:x:sp|P49167|RL38_YEAST:64
LIQSLPPTLKVNRL	sp|P49167|RL3	sp|P49167|RL38_YEAST	xlink	intra-protein xl	74	21	53	F1905_sailerc	2816.688	564.345	5.000	1.1	2	9	0.83	35.65	0.022	sp|P49167|RL38_YEAST:21:x:sp|P49167|RL38_YEAST:74
SSASSNSADAKAS	sp|P32794|AF(	sp|P32794|AFG2_YEAST	xlink	intra-protein xl	24	24	0	F1905_sailerc	2757.293	690.331	4.000	1.2	4	16	0.89	35.49	0.022	sp|P32794|AFG2_YEAST:24:x:sp|P32794|AFG2_YEAST:24
KSSASSNSADAK-	sp|P32794|AF(	sp|P32794|AFG2_YEAST	xlink	intra-protein xl	13	755	742	F1905_sailerc	2051.067	684.697	3.000	0.9	4	5	0.83	35.41	0.022	sp|P32794|AFG2_YEAST:13:x:sp|P32794|AFG2_YEAST:755
KKSSASSNSADAK	sp|P32794|AF(	sp|P32794|AFG2_YEAST	xlink	intra-protein xl	13	13	0	F1905_sailerc	2697.345	540.477	5.000	1.1	4	17	0.94	35.35	0.022	sp|P32794|AFG2_YEAST:13:x:sp|P32794|AFG2_YEAST:13
KLEIQQHAR-KVG	sp|P0CX25|RL4	sp|P0CX25|RL43A_YEAST	xlink	intra-protein xl	28	7	21	F1905_sailerc	1961.141	654.721	3.000	-0.6	7	6	0.87	35.27	0.022	sp|P0CX25|RL43A_YEAST:28:x:sp|P0CX25|RL43A_YEAST:7
VGKGDDEANGAT	sp|P14126|RL3	sp|P14126|RL3_YEAST	xlink	intra-protein xl	287	168	119	F1905_sailerc	2560.279	854.434	3.000	0.8	5	6	0.74	35.21	0.022	sp|P14126|RL3_YEAST:168:x:sp|P14126|RL3_YEAST:287
GVLLYGPPGCSKT	sp|P32794|AF(	sp|P32794|AFG2_YEAST	xlink	intra-protein xl	563	467	96	F1905_sailerc	2989.602	748.408	4.000	1.2	1	6	0.78	35.10	0.022	sp|P32794|AFG2_YEAST:467:x:sp|P32794|AFG2_YEAST:563
AKGIDIVDLAR-KE	sp|P53145|LSC	sp|P53145|LSG1_YEAST	xlink	intra-protein xl	544	534	10	F1905_sailerc	2322.296	581.582	4.000	2.2	1	7	0.92	35.09	0.022	sp|P53145|LSG1_YEAST:534:x:sp|P53145|LSG1_YEAST:544
ICANKYMTTVSGF	sp|P41805|RL1	sp|P41805|RL10_YEAST	xlink	intra-protein xl	74	158	84	F1905_sailerc	2632.310	878.444	3.000	-1.7	5	7	0.43	35.09	0.022	sp|P41805|RL10_YEAST:158:x:sp|P41805|RL10_YEAST:74
KNESQNSTAVTLC	sp|P47019|ALE	sp|P47019|ALB1_YEAST	xlink	intra-protein xl	63	108	45	F1905_sailerc	3672.919	919.238	4.000	0.5	7	6	0.90	35.08	0.022	sp|P47019|ALB1_YEAST:108:x:sp|P47019|ALB1_YEAST:63
VAAKIAALAGQQ-	sp|P26321|RL5	sp|P26321|RL5_YEAST	xlink	intra-protein xl	289	8	281	F1905_sailerc	2348.241	783.755	3.000	3.2	1	5	0.62	34.98	0.022	sp|P26321|RL5_YEAST:289:x:sp|P26321|RL5_YEAST:8
DKNILQESVLNK-	sp|Q03862|AR	sp|Q03862|ARX1_YEAST	xlink	intra-protein xl	17	83	66	F1905_sailerc	2296.245	766.423	3.000	2.9	2	4	0.63	34.54	0.022	sp|Q03862|ARX1_YEAST:17:x:sp|Q03862|ARX1_YEAST:83
ANMLENMQKSQ	sp|P38344|REI	sp|P38344|REI1_YEAST	xlink	intra-protein xl	105	90	15	F1905_sailerc	3213.639	804.418	4.000	0.5	4	5	0.55	34.48	0.022	sp|P38344|REI1_YEAST:105:x:sp|P38344|REI1_YEAST:90
ALVEHIIQAKADA	sp|P0CX82|RL1	sp|P0CX82|RL19A_YEAST	xlink	intra-protein xl	146	153	7	F1905_sailerc	3058.608	765.660	4.000	1.0	3	9	0.81	34.15	0.022	sp|P0CX82|RL19A_YEAST:146:x:sp|P0CX82|RL19A_YEAST:153
IGLVKYMSEK-VM	sp|P38344|REI	sp|P38344|REI1_YEAST	xlink	intra-protein xl	205	152	53	F1905_sailerc	2207.209	736.744	3.000	1.3	4	9	0.67	34.08	0.022	sp|P38344|REI1_YEAST:152:x:sp|P38344|REI1_YEAST:205
IGPLGLSPKK-VGE	sp|P0CX53|RL1	sp|P0CX53|RL12A_YEAST	xlink	intra-protein xl	40	48	8	F1905_sailerc	2177.272	726.765	3.000	2.4	6	10	0.66	33.87	0.022	sp|P0CX53|RL12A_YEAST:40:x:sp|P0CX53|RL12A_YEAST:48
VGKGDDEANGAT	sp|P14126|RL3	sp|P14126|RL3_YEAST	xlink	intra-protein xl	287	168	119	F1905_sailerc	2688.374	538.683	5.000	0.8	3	5	0.84	33.71	0.022	sp|P14126|RL3_YEAST:168:x:sp|P14126|RL3_YEAST:287
ANMLENMQKSQ	sp|P38344|REI	sp|P38344|REI1_YEAST	xlink	intra-protein xl	105	152	47	F1905_sailerc	3274.603	819.659	4.000	1.0	2	3	0.94	33.71	0.022	sp|P38344|REI1_YEAST:105:x:sp|P38344|REI1_YEAST:152
AEKDYELFLQELE	sp|P38861|NM	sp|P38861|NMD3_YEAST	xlink	intra-protein xl	444	436	8	F1905_sailerc	3495.676	1166.233	3.000	1.3	2	7	0.41	33.70	0.022	sp|P38861|NMD3_YEAST:436:x:sp|P38861|NMD3_YEAST:444
KYQPSIIFIDEIDSI	sp|P32794|AF(	sp|P32794|AFG2_YEAST	xlink	intra-protein xl	336	13	323	F1905_sailerc	3507.770	1170.265	3.000	-0.9	7	8	0.82	33.67	0.022	sp|P32794|AFG2_YEAST:13:x:sp|P32794|AFG2_YEAST:336
KSNEEAPR-QKLE	sp|P38202|YB(	sp|P38202|YBC8_YEAST	xlink	intra-protein xl	65	45	20	F1905_sailerc	1940.021	486.013	4.000	-0.5	2	6	0.89	33.40	0.022	sp|P38202|YBC8_YEAST:45:x:sp|P38202|YBC8_YEAST:65
GAVSGQKAGLIA	sp|P0CX45|RL2	sp|P0CX45|RL2A_YEAST	xlink	intra-protein xl	234	251	17	F1905_sailerc	2198.171	733.732	3.000	2.5	2	7	0.72	33.38	0.022	sp|P0CX45|RL2A_YEAST:234:x:sp|P0CX45|RL2A_YEAST:251

KYQPSIIFIDEIDSI/	sp|P32794|AF(	sp|P32794|AFG2_YEAST	xlink	intra-protein xl	336	24	312	F1905_sailerc	3665.853	1222.959	3.000	2.9	2	4	0.94	33.34	0.022	sp|P32794|AFG2_YEAST:24:x:sp|P32794|AFG2_YEAST:336
KNESQNSTAVTLC	sp|P47019|ALE	sp|P47019|ALB1_YEAST	xlink	intra-protein xl	63	102	39	F1905_sailerc	3721.897	931.482	4.000	2.8	3	5	0.61	33.31	0.022	sp|P47019|ALB1_YEAST:102:x:sp|P47019|ALB1_YEAST:63
SSASSNSADAKAS	sp|P32794|AF(	sp|P32794|AFG2_YEAST	xlink	intra-protein xl	24	118	94	F1905_sailerc	2677.360	893.461	3.000	1.6	6	14	0.70	33.17	0.022	sp|P32794|AFG2_YEAST:118:x:sp|P32794|AFG2_YEAST:24
VGEDIAKATK-EFK	sp|P0CX53|RL1	sp|P0CX53|RL12A_YEAST	xlink	intra-protein xl	48	54	6	F1905_sailerc	1889.053	473.271	4.000	1.0	3	7	0.88	33.05	0.022	sp|P0CX53|RL12A_YEAST:48:x:sp|P0CX53|RL12A_YEAST:54
VTVGSLQGYNILE(	sp|P32794|AF(	sp|P32794|AFG2_YEAST	xlink	intra-protein xl	146	168	22	F1905_sailerc	4894.535	979.915	5.000	2.1	4	12	0.67	33.04	0.022	sp|P32794|AFG2_YEAST:146:x:sp|P32794|AFG2_YEAST:168
GPEIFNKYVGESEI	sp|P32794|AF(	sp|P32794|AFG2_YEAST	xlink	intra-protein xl	589	755	166	F1905_sailerc	2523.319	631.837	4.000	2.5	1	5	0.76	33.02	0.022	sp|P32794|AFG2_YEAST:589:x:sp|P32794|AFG2_YEAST:755
KKSSASSNSADAK	sp|P32794|AF(	sp|P32794|AFG2_YEAST	xlink	intra-protein xl	13	118	105	F1905_sailerc	2647.385	662.854	4.000	1.4	14	7	0.92	33.00	0.022	sp|P32794|AFG2_YEAST:118:x:sp|P32794|AFG2_YEAST:13
KLVESNPELLR-EK	sp|Q07915|RLI	sp|Q07915|RLP24_YEAST	xlink	intra-protein xl	125	118	7	F1905_sailerc	2241.238	748.087	3.000	0.5	2	5	0.47	32.92	0.028	sp|Q07915|RLP24_YEAST:118:x:sp|Q07915|RLP24_YEAST:125
AQKQEMER-MAI	sp|P38861|NM	sp|P38861|NMD3_YEAST	xlink	intra-protein xl	436	419	17	F1905_sailerc	1898.936	633.987	3.000	1.1	3	7	0.65	32.87	0.028	sp|P38861|NMD3_YEAST:419:x:sp|P38861|NMD3_YEAST:436
TLTAKALATESGIN	sp|P32794|AF(	sp|P32794|AFG2_YEAST	xlink	intra-protein xl	568	589	21	F1905_sailerc	3708.967	928.250	4.000	1.6	4	8	0.88	32.76	0.028	sp|P32794|AFG2_YEAST:568:x:sp|P32794|AFG2_YEAST:589
LGISAPKGVLLYGF	sp|P32794|AF(	sp|P32794|AFG2_YEAST	xlink	intra-protein xl	551	755	204	F1905_sailerc	2812.572	704.151	4.000	1.5	2	3	0.64	32.58	0.028	sp|P32794|AFG2_YEAST:551:x:sp|P32794|AFG2_YEAST:755
ANQLLEKQK-ADL	sp|P53145|LSC	sp|P53145|LSG1_YEAST	xlink	intra-protein xl	274	243	31	F1905_sailerc	1996.157	500.047	4.000	-0.0	1	7	0.92	32.57	0.028	sp|P53145|LSG1_YEAST:243:x:sp|P53145|LSG1_YEAST:274
KKSSASSNSADAK	sp|P32794|AF(	sp|P32794|AFG2_YEAST	xlink	intra-protein xl	12	118	106	F1905_sailerc	2647.387	530.485	5.000	2.1	2	12	0.86	32.48	0.028	sp|P32794|AFG2_YEAST:118:x:sp|P32794|AFG2_YEAST:12
LGISAPKGVLLYGF	sp|P32794|AF(	sp|P32794|AFG2_YEAST	xlink	intra-protein xl	551	551	0	F1905_sailerc	3964.171	992.051	4.000	3.6	2	16	0.43	32.43	0.028	sp|P32794|AFG2_YEAST:551:x:sp|P32794|AFG2_YEAST:551
EQYAAESKK-LSKE	sp|P26321|RL5	sp|P26321|RL5_YEAST	xlink	intra-protein xl	270	279	9	F1905_sailerc	1950.994	651.339	3.000	1.9	2	6	0.91	32.32	0.028	sp|P26321|RL5_YEAST:270:x:sp|P26321|RL5_YEAST:279
SVALDLYFQNKK-!	sp|P47019|ALE	sp|P47019|ALB1_YEAST	xlink	intra-protein xl	62	46	16	F1905_sailerc	2697.428	900.151	3.000	2.1	3	6	0.62	32.24	0.028	sp|P47019|ALB1_YEAST:46:x:sp|P47019|ALB1_YEAST:62
VRLPSGAKK-GSQ	sp|P0CX45|RL2	sp|P0CX45|RL2A_YEAST	xlink	intra-protein xl	155	251	96	F1905_sailerc	1855.018	464.762	4.000	1.1	2	8	0.80	32.18	0.028	sp|P0CX45|RL2A_YEAST:155:x:sp|P0CX45|RL2A_YEAST:251
KSSASSNSADAKA	sp|P32794|AF(	sp|P32794|AFG2_YEAST	xlink	intra-protein xl	24	24	0	F1905_sailerc	2885.388	578.085	5.000	1.1	1	9	0.77	32.03	0.028	sp|P32794|AFG2_YEAST:24:x:sp|P32794|AFG2_YEAST:24
QATKDAGTIAGLN	sp|P10591|HSI	sp|P10591|HSP71_YEAST	xlink	intra-protein xl	157	509	352	F1905_sailerc	2725.489	909.504	3.000	1.0	4	6	0.48	32.03	0.028	sp|P10591|HSP71_YEAST:157:x:sp|P10591|HSP71_YEAST:509

monolinks

KFNTEESGVDLHE	sp|P32794|AF(	-	monolink	monolink	699	n/a	-	F1905_sailerc	2114.032	705.685	3.000	1.8	4	35	0.35	54.11	0.000	sp|P32794|AFG2_YEAST:699
KTVKAQRPITGAS	sp|P04449|RL2	-	monolink	monolink	77,77	n/a	-	F1905_sailerc	2094.243	524.569	4.000	0.8	4	33	0.67	52.89	0.000	sp|P04449|RL24A_YEAST:77:|:sp|P24000|RL24B_YEAST:77
TRDLDLIYNDLSTK	sp|Q08004|BU	-	monolink	monolink	28	n/a	-	F1905_sailerc	2393.235	798.753	3.000	0.8	6	28	0.39	52.44	0.000	sp|Q08004|BUD20_YEAST:28
AEKDYELFLQELEE	sp|P38861|NM	-	monolink	monolink	444	n/a	-	F1905_sailerc	2495.199	832.741	3.000	1.3	8	24	0.30	52.35	0.000	sp|P38861|NMD3_YEAST:444
VIQKLLDDSGVIM	sp|P32794|AF(	-	monolink	monolink	150	n/a	-	F1905_sailerc	2614.414	654.611	4.000	1.0	19	33	0.39	52.28	0.000	sp|P32794|AFG2_YEAST:150
TLTAKALATESGIN	sp|P32794|AF(	-	monolink	monolink	568	n/a	-	F1905_sailerc	2102.204	701.742	3.000	2.4	23	29	0.52	52.17	0.000	sp|P32794|AFG2_YEAST:568
FDILTKQFSR-K6-1	sp|P32794|AF(	-	monolink	monolink	425	n/a	-	F1905_sailerc	1409.757	470.927	3.000	1.2	2	21	0.30	51.92	0.000	sp|P32794|AFG2_YEAST:425
MEEHMSTLGHDN	sp|Q02892|NC	-	monolink	monolink	548	n/a	-	F1905_sailerc	2613.165	872.063	3.000	1.2	6	29	0.25	51.85	0.000	sp|Q02892|NOG1_YEAST:548
TLTAKALATESGIN	sp|P32794|AF(	-	monolink	monolink	568	n/a	-	F1905_sailerc	2103.184	702.069	3.000	0.7	105	29	0.47	51.77	0.000	sp|P32794|AFG2_YEAST:568
VLLSAPLSKELR-KS	sp|P05743|RL2	-	monolink	monolink	37,37	n/a	-	F1905_sailerc	1480.887	494.637	3.000	0.5	2	21	0.59	51.59	0.000	sp|P05743|RL26A_YEAST:37:|:sp|P53221|RL26B_YEAST:37
TLASVTKEILGTAC	sp|P0CX53|RL1	-	monolink	monolink	130	n/a	-	F1905_sailerc	2145.151	716.058	3.000	2.3	3	33	0.62	50.32	0.000	sp|P0CX53|RL12A_YEAST:130
AEKDYELFLQELEE	sp|P38861|NM	-	monolink	monolink	444	n/a	-	F1905_sailerc	2494.213	832.412	3.000	0.7	5	24	0.34	50.31	0.000	sp|P38861|NMD3_YEAST:444
RAEAKAQGVAVG	sp|Q02753|RL	-	monolink	monolink	120,120	n/a	-	F1905_sailerc	1622.949	541.991	3.000	1.4	3	24	0.50	50.02	0.000	sp|Q02753|RL21A_YEAST:120:|:sp|Q12672|RL21B_YEAST:120
VLIDGPKAGVPR-I	sp|P36105|RL1	-	monolink	monolink	50,50	n/a	-	F1905_sailerc	1376.804	459.942	3.000	0.9	2	22	0.00	49.85	0.000	sp|P36105|RL14A_YEAST:50:|:sp|P38754|RL14B_YEAST:50
LPAASLGDMVMA	sp|P0CX41|RL2	-	monolink	monolink	63	n/a	-	F1905_sailerc	1786.961	596.662	3.000	2.1	3	25	0.29	49.56	0.000	sp|P0CX41|RL23A_YEAST:63
RGGQSKACPVGN	sp|P40010|NU	-	monolink	monolink	309	n/a	-	F1905_sailerc	2300.156	767.727	3.000	0.9	6	29	0.35	49.04	0.000	sp|P40010|NUG1_YEAST:309
AQVQPPYATKVT	sp|P32794|AF(	-	monolink	monolink	128	n/a	-	F1905_sailerc	3308.637	1103.887	3.000	1.8	83	28	0.21	48.62	0.000	sp|P32794|AFG2_YEAST:128
FDILTKQFSR-K6-1	sp|P32794|AF(	-	monolink	monolink	425	n/a	-	F1905_sailerc	1408.773	470.599	3.000	1.0	2	20	0.32	48.48	0.000	sp|P32794|AFG2_YEAST:425
SVKFVQGLLQNA	sp|P05740|RL1	-	monolink	monolink	89,89	n/a	-	F1905_sailerc	2114.140	705.721	3.000	1.1	6	30	0.47	48.39	0.000	sp|P05740|RL17A_YEAST:89:|:sp|P46990|RL17B_YEAST:89
EIFLEMPKVYWSD	sp|P32794|AF(	-	monolink	monolink	512	n/a	-	F1905_sailerc	2665.336	889.453	3.000	0.4	5	31	0.45	48.23	0.000	sp|P32794|AFG2_YEAST:512
HAKQGESDRHNA	sp|Q02892|NC	-	monolink	monolink	618	n/a	-	F1905_sailerc	2018.031	505.515	4.000	0.6	4	26	0.46	48.16	0.000	sp|Q02892|NOG1_YEAST:618
RAEAKAQGVAVG	sp|Q02753|RL	-	monolink	monolink	120,120	n/a	-	F1905_sailerc	1623.935	542.319	3.000	2.3	1	26	0.45	48.01	0.000	sp|Q02753|RL21A_YEAST:120:|:sp|Q12672|RL21B_YEAST:120
LKVPPTIAQFQYTI	sp|P17076|RL8	-	monolink	monolink	70,70	n/a	-	F1905_sailerc	2045.124	682.716	3.000	2.0	3	22	0.44	47.48	0.000	sp|P17076|RL8A_YEAST:70:|:sp|P29453|RL8B_YEAST:70
LLPFVKGNVGFVF	sp|P05317|RLA	-	monolink	monolink	81	n/a	-	F1905_sailerc	2617.450	873.491	3.000	3.7	3	27	0.44	47.47	0.000	sp|P05317|RLA0_YEAST:81
SVTQESALDEFLST	sp|P53145|LSC	-	monolink	monolink	86	n/a	-	F1905_sailerc	2955.433	986.152	3.000	-0.6	3	28	0.37	47.43	0.000	sp|P53145|LSG1_YEAST:86
NILQESVLNKYR-K	sp|Q03862|AR	-	monolink	monolink	27	n/a	-	F1905_sailerc	1631.890	544.971	3.000	1.0	2	21	0.41	47.23	0.000	sp|Q03862|ARX1_YEAST:27
ALVEHIIQAKADA(	sp|P0CX82|RL1	-	monolink	monolink	146	n/a	-	F1905_sailerc	1817.017	606.680	3.000	0.6	2	23	0.61	47.20	0.000	sp|P0CX82|RL19A_YEAST:146
CTKKFNTEESGVD	sp|P32794|AF(	-	monolink	monolink	699	n/a	-	F1905_sailerc	2504.189	835.738	3.000	1.6	1	25	0.41	47.17	0.000	sp|P32794|AFG2_YEAST:699
VYWSDIGGQEELI	sp|P32794|AF(	-	monolink	monolink	525	n/a	-	F1905_sailerc	1906.970	636.664	3.000	1.0	2	22	0.47	47.03	0.000	sp|P32794|AFG2_YEAST:525
VTVGSLQGYNILE(	sp|P32794|AF(	-	monolink	monolink	146	n/a	-	F1905_sailerc	2693.363	674.349	4.000	-1.0	27	36	0.50	46.91	0.000	sp|P32794|AFG2_YEAST:146
SKEQLASQLVDLK	sp|P0CX84|RL3	-	monolink	monolink	14	n/a	-	F1905_sailerc	1741.985	581.669	3.000	1.2	4	22	0.44	46.73	0.000	sp|P0CX84|RL35A_YEAST:14
GSSSLYTLVINDA(	sp|P49167|RL3	-	monolink	monolink	61	n/a	-	F1905_sailerc	1878.997	627.340	3.000	1.6	4	26	0.35	46.55	0.000	sp|P49167|RL38_YEAST:61
GHRVEKIPEIPLVV	sp|P10664|RL4	-	monolink	monolink	144,144	n/a	-	F1905_sailerc	2742.526	686.639	4.000	3.5	13	24	0.60	46.55	0.000	sp|P10664|RL4A_YEAST:144:|:sp|P49626|RL4B_YEAST:144
EITDIKQFLELTR-K	sp|P49167|RL3	-	monolink	monolink	9	n/a	-	F1905_sailerc	1760.957	587.994	3.000	0.8	4	23	0.65	46.42	0.000	sp|P49167|RL38_YEAST:9
MKEMIQLPLEASE	sp|P32794|AF(	-	monolink	monolink	529	n/a	-	F1905_sailerc	2149.082	717.369	3.000	1.1	17	22	0.39	46.33	0.000	sp|P32794|AFG2_YEAST:529
YIASKTHGYVGAD	sp|P32794|AF(	-	monolink	monolink	448	n/a	-	F1905_sailerc	2251.134	751.386	3.000	1.3	21	25	0.47	46.28	0.000	sp|P32794|AFG2_YEAST:448
GVVWTESHQEAD	sp|Q03862|AR	-	monolink	monolink	285	n/a	-	F1905_sailerc	3153.550	1052.191	3.000	1.1	9	23	0.36	46.13	0.000	sp|Q03862|ARX1_YEAST:285
IINEPTAAAIAYGL	sp|P10591|HSI	-	monolink	monolink	185	n/a	-	F1905_sailerc	1943.064	648.696	3.000	1.2	1	26	0.51	45.97	0.000	sp|P10591|HSP71_YEAST:185
YNLPEPLSYAAVG	sp|P32794|AF(	-	monolink	monolink	252	n/a	-	F1905_sailerc	2661.384	888.136	3.000	2.0	7	26	0.43	45.94	0.000	sp|P32794|AFG2_YEAST:252
VTLKDVESAMVD	sp|P32794|AF(	-	monolink	monolink	489	n/a	-	F1905_sailerc	2273.178	569.302	4.000	1.1	20	23	0.47	45.92	0.000	sp|P32794|AFG2_YEAST:489
SALGETQKDTYQ\	sp|P53742|NO	-	monolink	monolink	101	n/a	-	F1905_sailerc	1977.045	660.023	3.000	1.6	2	26	0.53	45.88	0.000	sp|P53742|NOG2_YEAST:101
TKEAVAALKAVGA	sp|P10664|RL4	-	monolink	monolink	170,170	n/a	-	F1905_sailerc	2076.195	520.057	4.000	0.2	2	27	0.64	45.87	0.000	sp|P10664|RL4A_YEAST:170:|:sp|P49626|RL4B_YEAST:170
VLEQLSGQTPVQS	sp|P0C0W9|RL	-	monolink	monolink	49,49	n/a	-	F1905_sailerc	1895.051	632.691	3.000	1.5	2	21	0.42	45.74	0.000	sp|P0C0W9|RL11A_YEAST:49:|:sp|Q3E757|RL11B_YEAST:49
MKEMIQLPLEASE	sp|P32794|AF(	-	monolink	monolink	529	n/a	-	F1905_sailerc	2148.103	717.042	3.000	3.2	7	26	0.35	45.67	0.000	sp|P32794|AFG2_YEAST:529
EITDIKQFLELTR-K	sp|P49167|RL3	-	monolink	monolink	9	n/a	-	F1905_sailerc	1759.976	587.666	3.000	2.1	2	22	0.69	45.67	0.000	sp|P49167|RL38_YEAST:9
IVKAGGECITLDQI	sp|P0CX49|RL1	-	monolink	monolink	116	n/a	-	F1905_sailerc	1998.088	667.037	3.000	3.2	1	20	0.63	45.35	0.000	sp|P0CX49|RL18A_YEAST:116
TAGQIAQTALKYV	sp|Q03862|AR	-	monolink	monolink	40	n/a	-	F1905_sailerc	2763.447	691.870	4.000	1.1	5	27	0.50	45.31	0.000	sp|Q03862|ARX1_YEAST:40
AQVQPPYATKVT	sp|P32794|AF(	-	monolink	monolink	128	n/a	-	F1905_sailerc	3307.656	1103.560	3.000	2.8	28	32	0.21	45.07	0.000	sp|P32794|AFG2_YEAST:128
FKLPAEFITRPHPS	sp|P32794|AF(	-	monolink	monolink	29	n/a	-	F1905_sailerc	1922.080	481.528	4.000	0.9	2	24	0.51	44.83	0.000	sp|P32794|AFG2_YEAST:29
KAQVQPPYATKV	sp|P32794|AF(	-	monolink	monolink	128	n/a	-	F1905_sailerc	3436.727	860.190	4.000	0.3	38	25	0.36	44.79	0.000	sp|P32794|AFG2_YEAST:128
LLDDSGVIMPGM	sp|P32794|AF(	-	monolink	monolink	168	n/a	-	F1905_sailerc	2375.252	792.758	3.000	1.6	20	25	0.35	44.77	0.000	sp|P32794|AFG2_YEAST:168
GSTHITFSKETQAI	sp|P32794|AF(	-	monolink	monolink	228	n/a	-	F1905_sailerc	1960.005	654.343	3.000	1.7	6	23	0.51	44.55	0.000	sp|P32794|AFG2_YEAST:228
KVACIGAWHPAH	sp|P14126|RL3	-	monolink	monolink	248	n/a	-	F1905_sailerc	2330.198	583.557	4.000	2.1	3	30	0.54	44.51	0.000	sp|P14126|RL3_YEAST:248
TKEAVAALKAVGA	sp|P10664|RL4	-	monolink	monolink	170,170	n/a	-	F1905_sailerc	2077.181	520.303	4.000	1.2	5	25	0.57	44.45	0.000	sp|P10664|RL4A_YEAST:170:|:sp|P49626|RL4B_YEAST:170
KANLLLVNKADLL	sp|P53145|LSC	-	monolink	monolink	237	n/a	-	F1905_sailerc	1809.099	604.041	3.000	0.5	2	25	0.57	44.40	0.000	sp|P53145|LSG1_YEAST:237
KYNLPEPLSYAAV(	sp|P32794|AF(	-	monolink	monolink	252	n/a	-	F1905_sailerc	2789.480	930.834	3.000	2.2	9	24	0.55	44.35	0.000	sp|P32794|AFG2_YEAST:252
AERVEDQKVVDK	sp|Q02326|RL(	-	monolink	monolink	139	n/a	-	F1905_sailerc	1570.821	524.615	3.000	0.6	2	20	0.55	44.30	0.000	sp|Q02326|RL6A_YEAST:139
VTLKDVESAMVD	sp|P32794|AF(	-	monolink	monolink	489	n/a	-	F1905_sailerc	2272.193	569.056	4.000	0.4	11	20	0.44	44.30	0.000	sp|P32794|AFG2_YEAST:489
SKEQLASQLVDLK	sp|P0CX84|RL3	-	monolink	monolink	25	n/a	-	F1905_sailerc	1741.985	581.669	3.000	1.4	2	26	0.43	44.17	0.000	sp|P0CX84|RL35A_YEAST:25
KLRDPLAYLEQVR	sp|Q02892|NC	-	monolink	monolink	142	n/a	-	F1905_sailerc	1755.009	586.011	3.000	2.4	4	21	0.58	44.01	0.000	sp|Q02892|NOG1_YEAST:142
NVKHSGNIQLDEI	sp|P0CX53|RL1	-	monolink	monolink	99	n/a	-	F1905_sailerc	2204.183	735.735	3.000	1.1	5	25	0.52	43.94	0.000	sp|P0CX53|RL12A_YEAST:99
KVLIDGPKAGVPR	sp|P36105|RL1	-	monolink	monolink	50,50	n/a	-	F1905_sailerc	1504.901	502.642	3.000	2.2	5	19	0.41	43.93	0.000	sp|P36105|RL14A_YEAST:50:|:sp|P38754|RL14B_YEAST:50
SSVGVIINKMVGN	sp|Q02753|RL	-	monolink	monolink	78,78	n/a	-	F1905_sailerc	1628.895	543.973	3.000	1.5	4	22	0.54	43.91	0.000	sp|Q02753|RL21A_YEAST:78:|:sp|Q12672|RL21B_YEAST:78
GLDATKLYVSHIQ'	sp|P05740|RL1	-	monolink	monolink	111,111	n/a	-	F1905_sailerc	2237.211	746.745	3.000	2.2	8	25	0.56	43.85	0.000	sp|P05740|RL17A_YEAST:111:|:sp|P46990|RL17B_YEAST:111
NHYKISLAAISR-K	sp|Q02892|NC	-	monolink	monolink	92	n/a	-	F1905_sailerc	1527.842	510.288	3.000	0.6	2	22	0.61	43.32	0.000	sp|Q02892|NOG1_YEAST:92
WAAAAVCEKWA.	sp|P36105|RL1	-	monolink	monolink	93	n/a	-	F1905_sailerc	2047.985	683.669	3.000	1.1	2	22	0.33	43.26	0.000	sp|P36105|RL14A_YEAST:93
GLGTDANIDKFSLI	sp|P32794|AF(	-	monolink	monolink	481	n/a	-	F1905_sailerc	1633.860	545.628	3.000	2.5	6	24	0.39	43.07	0.000	sp|P32794|AFG2_YEAST:481
GFTLAEVKAAGLT	sp|Q12690|RL	-	monolink	monolink	81,81	n/a	-	F1905_sailerc	1965.063	656.029	3.000	3.2	2	26	0.48	43.05	0.000	sp|P40212|RL13B_YEAST:81:|:sp|Q12690|RL13A_YEAST:81
VAQLPPISFETFDS	sp|P38344|REI	-	monolink	monolink	53	n/a	-	F1905_sailerc	2707.398	903.474	3.000	0.7	8	27	0.39	43.02	0.000	sp|P38344|REI1_YEAST:53
YIASKTHGYVGAD	sp|P32794|AF(	-	monolink	monolink	448	n/a	-	F1905_sailerc	2250.147	751.057	3.000	0.1	7	30	0.53	42.92	0.000	sp|P32794|AFG2_YEAST:448
YVYAHFPINVNIVI	sp|P05738|RL9	-	monolink	monolink	106,106	n/a	-	F1905_sailerc	2332.215	778.413	3.000	2.1	3	28	0.51	42.81	0.000	sp|P05738|RL9A_YEAST:106:|:sp|P51401|RL9B_YEAST:106
IFEFPKTTVAALR-I	sp|P0CX49|RL1	-	monolink	monolink	98	n/a	-	F1905_sailerc	1647.927	550.317	3.000	1.9	2	24	0.67	42.78	0.000	sp|P0CX49|RL18A_YEAST:98
ALVEHIIQAKADA(	sp|P0CX82|RL1	-	monolink	monolink	146	n/a	-	F1905_sailerc	1818.002	607.008	3.000	1.1	4	20	0.55	42.53	0.000	sp|P0CX82|RL19A_YEAST:146
SFGKMEEHMSTL(	sp|Q02892|NC	-	monolink	monolink	530	n/a	-	F1905_sailerc	2634.182	879.069	3.000	2.2	6	24	0.28	42.38	0.000	sp|Q02892|NOG1_YEAST:530
KAQVQPPYATKV	sp|P32794|AF(	-	monolink	monolink	118	n/a	-	F1905_sailerc	3435.741	859.943	4.000	-0.3	4	25	0.36	42.31	0.000	sp|P32794|AFG2_YEAST:118
HVLDSEAIKYIASK	sp|P32794|AF(	-	monolink	monolink	443	n/a	-	F1905_sailerc	1728.933	577.319	3.000	1.7	3	22	0.56	42.16	0.000	sp|P32794|AFG2_YEAST:443
LVNHFIQEFKR-K1	sp|P10591|HSI	-	monolink	monolink	243	n/a	-	F1905_sailerc	1585.862	529.629	3.000	0.5	2	16	0.47	42.14	0.000	sp|P10591|HSP71_YEAST:243
ITIDSPYLVCTEGEI	sp|P33201|MR	-	monolink	monolink	194	n/a	-	F1905_sailerc	2363.191	788.738	3.000	-0.9	5	22	0.45	42.12	0.000	sp|P33201|MRT4_YEAST:194
LLLNSLNALKLINE	sp|Q12019|MI	-	monolink	monolink	2480	n/a	-	F1905_sailerc	1851.110	618.044	3.000	0.7	1	24	0.70	42.07	0.000	sp|Q12019|MDN1_YEAST:2480
KRPVPKGATYGKF	sp|P05748|RL1	-	monolink	monolink	77,77	n/a	-	F1905_sailerc	2537.401	635.358	4.000	1.7	5	21	0.48	42.05	0.000	sp|P05748|RL15A_YEAST:77:|:sp|P54780|RL15B_YEAST:77
FKLPAEFITRPHPS	sp|P32794|AF(	-	monolink	monolink	29	n/a	-	F1905_sailerc	1923.064	481.774	4.000	1.0	4	20	0.50	42.04	0.000	sp|P32794|AFG2_YEAST:29
LCSGVTGLLFTDEI	sp|P33201|MR	-	monolink	monolink	113	n/a	-	F1905_sailerc	2790.370	931.131	3.000	1.0	5	24	0.34	42.00	0.000	sp|P33201|MRT4_YEAST:113
GILLHGPPGTGKT	sp|P32794|AF(	-	monolink	monolink	292	n/a	-	F1905_sailerc	1915.110	639.378	3.000	1.1	3	22	0.52	41.98	0.000	sp|P32794|AFG2_YEAST:292
SKEQLASQLVDLK	sp|P0CX84|RL3	-	monolink	monolink	14	n/a	-	F1905_sailerc	1741.003	581.342	3.000	2.6	2	23	0.52	41.94	0.000	sp|P0CX84|RL35A_YEAST:14
KKVMPAIVVR-K1	sp|P0CX41|RL2	-	monolink	monolink	71	n/a	-	F1905_sailerc	1295.801	432.941	3.000	0.6	2	16	0.29	41.93	0.000	sp|P0CX41|RL23A_YEAST:71
KLRDPLAYLEQVR	sp|Q02892|NC	-	monolink	monolink	142	n/a	-	F1905_sailerc	1755.988	586.337	3.000	0.1	2	17	0.69	41.91	0.000	sp|Q02892|NOG1_YEAST:142
KEQVQNAAKAK-I	sp|P53145|LSC	-	monolink	monolink	542	n/a	-	F1905_sailerc	1369.757	457.594	3.000	0.7	3	22	0.50	41.90	0.000	sp|P53145|LSG1_YEAST:542
HALHGTAKALAA/	sp|P05747|RL2	-	monolink	monolink	52	n/a	-	F1905_sailerc	1513.874	505.632	3.000	0.5	4	32	0.73	41.80	0.000	sp|P05747|RL29_YEAST:52
VDKPLLKAGR-K7-	sp|P0CX45|RL2	-	monolink	monolink	181	n/a	-	F1905_sailerc	1251.755	418.260	3.000	0.1	2	15	0.00	41.76	0.000	sp|P0CX45|RL2A_YEAST:181
HVLDSEAIKYIASK	sp|P32794|AF(	-	monolink	monolink	443	n/a	-	F1905_sailerc	1727.948	576.990	3.000	1.2	2	21	0.76	41.73	0.000	sp|P32794|AFG2_YEAST:443
DLDLIYNDLSTKES	sp|Q08004|BU	-	monolink	monolink	28	n/a	-	F1905_sailerc	2136.084	713.036	3.000	0.3	6	22	0.61	41.66	0.000	sp|Q08004|BUD20_YEAST:28
NTAAETFKLFNK-I	sp|P17076|RL8	-	monolink	monolink	92,92	n/a	-	F1905_sailerc	1538.800	513.941	3.000	1.4	2	21	0.48	41.66	0.000	sp|P17076|RL8A_YEAST:92:|:sp|P29453|RL8B_YEAST:92
VTVGSLQGYNILE(	sp|P32794|AF(	-	monolink	monolink	146	n/a	-	F1905_sailerc	2692.383	674.104	4.000	0.7	9	33	0.54	41.54	0.000	sp|P32794|AFG2_YEAST:146
ASKVLEQLSGQTP	sp|P0C0W9|RL	-	monolink	monolink	35,35	n/a	-	F1905_sailerc	1955.062	652.695	3.000	2.4	3	25	0.46	41.49	0.000	sp|P0C0W9|RL11A_YEAST:35:|:sp|Q3E757|RL11B_YEAST:35
VVATLLTLMDGM	sp|P32794|AF(	-	monolink	monolink	382	n/a	-	F1905_sailerc	3675.027	919.764	4.000	1.4	4	27	0.42	41.44	0.000	sp|P32794|AFG2_YEAST:382
GPEIFNKYVGESEI	sp|P32794|AF(	-	monolink	monolink	589	n/a	-	F1905_sailerc	1778.886	593.970	3.000	1.3	2	24	0.53	41.39	0.000	sp|P32794|AFG2_YEAST:589
KFLDGIYVSHK-K1	sp|P05738|RL9	-	monolink	monolink	174,174	n/a	-	F1905_sailerc	1460.803	487.942	3.000	0.4	2	19	0.60	41.38	0.000	sp|P05738|RL9A_YEAST:174:|:sp|P51401|RL9B_YEAST:174
GLGTDANIDKFSLI	sp|P32794|AF(	-	monolink	monolink	481	n/a	-	F1905_sailerc	1632.874	545.299	3.000	1.0	3	27	0.57	41.18	0.000	sp|P32794|AFG2_YEAST:481
KSELEYYAMLSKTI	sp|P14120|RL3	-	monolink	monolink	64	n/a	-	F1905_sailerc	1845.945	616.323	3.000	0.7	1	19	0.54	41.16	0.000	sp|P14120|RL30_YEAST:64
HALHGTAKALAA/	sp|P05747|RL2	-	monolink	monolink	52	n/a	-	F1905_sailerc	1514.858	505.961	3.000	1.0	7	30	0.61	41.12	0.000	sp|P05747|RL29_YEAST:52
LSRPSLPKIK-K8-1	sp|P0CX84|RL3	-	monolink	monolink	43	n/a	-	F1905_sailerc	1293.803	432.276	3.000	1.1	2	16	0.51	41.03	0.000	sp|P0CX84|RL35A_YEAST:43
ELVATTLKAMAR-	sp|Q07915|RLI	-	monolink	monolink	91	n/a	-	F1905_sailerc	1458.814	487.279	3.000	2.0	2	19	0.31	40.87	0.000	sp|Q07915|RLP24_YEAST:91
QALILKQFGIAASE	sp|P33201|MR	-	monolink	monolink	204	n/a	-	F1905_sailerc	1918.096	640.373	3.000	1.6	2	22	0.51	40.87	0.000	sp|P33201|MRT4_YEAST:204
KVVIVKPHDEGSK	sp|P0C2H6|RL	-	monolink	monolink	27,27	n/a	-	F1905_sailerc	1590.900	531.308	3.000	1.3	4	19	0.63	40.84	0.000	sp|P0C2H6|RL27A_YEAST:27:|:sp|P0C2H7|RL27B_YEAST:27
TVKAQRPITGASLI	sp|P04449|RL2	-	monolink	monolink	77,77	n/a	-	F1905_sailerc	1966.150	656.391	3.000	1.8	4	26	0.55	40.52	0.000	sp|P04449|RL24A_YEAST:77:|:sp|P24000|RL24B_YEAST:77
TAGQIAQTALKYV	sp|Q03862|AR	-	monolink	monolink	40	n/a	-	F1905_sailerc	2764.432	692.116	4.000	1.2	25	24	0.45	40.44	0.000	sp|Q03862|ARX1_YEAST:40
SCGVDAMSVDDL	sp|P0CX43|RL1	-	monolink	monolink	91	n/a	-	F1905_sailerc	1679.775	560.933	3.000	0.1	3	21	0.38	40.39	0.000	sp|P0CX43|RL1A_YEAST:91
GRIPNVPVIVKAR-	sp|P02406|RL2	-	monolink	monolink	126	n/a	-	F1905_sailerc	1572.983	525.336	3.000	0.4	2	18	0.42	40.39	0.000	sp|P02406|RL28_YEAST:126
KLEIQQHAR-K1-1	sp|P0CX25|RL4	-	monolink	monolink	28	n/a	-	F1905_sailerc	1277.710	426.911	3.000	0.9	1	17	0.29	40.30	0.000	sp|P0CX25|RL43A_YEAST:28
EIFLEMPKVYWSD	sp|P32794|AF(	-	monolink	monolink	512	n/a	-	F1905_sailerc	2666.323	889.782	3.000	1.5	23	26	0.42	40.28	0.000	sp|P32794|AFG2_YEAST:512
ILGWVLPNTSAA/	sp|P40010|NU	-	monolink	monolink	466	n/a	-	F1905_sailerc	3211.668	1071.564	3.000	-1.3	4	25	0.41	40.25	0.000	sp|P40010|NUG1_YEAST:466
AEKTDLTNILIR-K3	sp|P39715|ECI	-	monolink	monolink	57	n/a	-	F1905_sailerc	1541.867	514.964	3.000	0.5	4	21	0.66	40.20	0.000	sp|P39715|ECM1_YEAST:57
ELVATTLKAMAR-	sp|Q07915|RLI	-	monolink	monolink	91	n/a	-	F1905_sailerc	1457.828	486.950	3.000	0.1	2	18	0.63	40.08	0.000	sp|Q07915|RLP24_YEAST:91
VYWSDIGGQEELI	sp|P32794|AF(	-	monolink	monolink	525	n/a	-	F1905_sailerc	1907.956	636.993	3.000	2.0	4	23	0.39	40.06	0.000	sp|P32794|AFG2_YEAST:525
KGQSAGKLAAIVE	sp|P36105|RL1	-	monolink	monolink	31,31	n/a	-	F1905_sailerc	2024.156	675.727	3.000	2.3	4	29	0.64	40.05	0.000	sp|P36105|RL14A_YEAST:31:|:sp|P38754|RL14B_YEAST:31
VVATLLTLMDGM	sp|P32794|AF(	-	monolink	monolink	382	n/a	-	F1905_sailerc	3676.010	920.010	4.000	1.3	6	23	0.37	39.94	0.000	sp|P32794|AFG2_YEAST:382
LSHLSSNFPFVHEI	sp|Q03862|AR	-	monolink	monolink	413	n/a	-	F1905_sailerc	2867.439	956.821	3.000	1.5	13	26	0.37	39.90	0.000	sp|Q03862|ARX1_YEAST:413
MSSDRHVLDSEAI	sp|P32794|AF(	-	monolink	monolink	443	n/a	-	F1905_sailerc	2304.183	577.054	4.000	2.1	5	16	0.62	39.84	0.000	sp|P32794|AFG2_YEAST:443
KVVIVKPHDEGSK	sp|P0C2H6|RL	-	monolink	monolink	27,27	n/a	-	F1905_sailerc	1589.918	530.981	3.000	2.5	5	19	0.71	39.77	0.000	sp|P0C2H6|RL27A_YEAST:27:|:sp|P0C2H7|RL27B_YEAST:27
GIVKQIVHDSGR-I	sp|P0CX45|RL2	-	monolink	monolink	46	n/a	-	F1905_sailerc	1462.827	488.617	3.000	0.9	2	19	0.43	39.76	0.000	sp|P0CX45|RL2A_YEAST:46
IPPKPRKVLQLLR-I	sp|P05737|RL7	-	monolink	monolink	101,101	n/a	-	F1905_sailerc	1713.105	429.284	4.000	1.0	1	18	0.91	39.73	0.000	sp|P05737|RL7A_YEAST:101:|:sp|Q12213|RL7B_YEAST:101
LLDDSGVIMPGM	sp|P32794|AF(	-	monolink	monolink	168	n/a	-	F1905_sailerc	2374.270	792.431	3.000	2.7	7	23	0.41	39.70	0.000	sp|P32794|AFG2_YEAST:168
GQSAGKLAAIVEII	sp|P36105|RL1	-	monolink	monolink	31,31	n/a	-	F1905_sailerc	1896.060	633.028	3.000	1.9	1	25	0.62	39.68	0.000	sp|P36105|RL14A_YEAST:31:|:sp|P38754|RL14B_YEAST:31
IINSSEIQSAIRPAG	sp|P10664|RL4	-	monolink	monolink	308,308	n/a	-	F1905_sailerc	2422.335	808.453	3.000	2.2	5	24	0.46	39.65	0.000	sp|P10664|RL4A_YEAST:308:|:sp|P49626|RL4B_YEAST:308
GIVKQIVHDSGR-I	sp|P0CX45|RL2	-	monolink	monolink	46	n/a	-	F1905_sailerc	1463.810	488.944	3.000	-0.0	4	18	0.38	39.62	0.000	sp|P0CX45|RL2A_YEAST:46
GQSAGKLAAIVEII	sp|P36105|RL1	-	monolink	monolink	31,31	n/a	-	F1905_sailerc	1895.076	632.700	3.000	1.9	3	24	0.62	39.60	0.000	sp|P36105|RL14A_YEAST:31:|:sp|P38754|RL14B_YEAST:31
ALATESGINFLAVI	sp|P32794|AF(	-	monolink	monolink	582	n/a	-	F1905_sailerc	2373.299	792.107	3.000	1.8	5	29	0.46	39.54	0.000	sp|P32794|AFG2_YEAST:582
LKEYQSKIIVFPR-K	sp|Q12690|RL	-	monolink	monolink	122,122	n/a	-	F1905_sailerc	1776.021	593.015	3.000	1.2	2	19	0.64	39.53	0.000	sp|P40212|RL13B_YEAST:122:|:sp|Q12690|RL13A_YEAST:122
KTVAGGAYTVSTA	sp|P0CX25|RL4	-	monolink	monolink	62	n/a	-	F1905_sailerc	1950.045	651.023	3.000	1.6	2	20	0.64	39.53	0.000	sp|P0CX25|RL43A_YEAST:62
LFHSIKPLFANK-KI	sp|Q02892|NC	-	monolink	monolink	275	n/a	-	F1905_sailerc	1568.909	523.977	3.000	0.5	2	21	0.54	39.44	0.000	sp|Q02892|NOG1_YEAST:275
VEKIPEIPLVVSTDI	sp|P10664|RL4	-	monolink	monolink	144,144	n/a	-	F1905_sailerc	2392.339	798.454	3.000	1.4	7	20	0.37	39.36	0.000	sp|P10664|RL4A_YEAST:144:|:sp|P49626|RL4B_YEAST:144
AKNPLTHSTPK-K2	sp|P29453|RL8	-	monolink	monolink	23	n/a	-	F1905_sailerc	1347.752	450.259	3.000	0.8	3	18	0.31	39.34	0.000	sp|P29453|RL8B_YEAST:23
VVANTSNAHVLTI	sp|P32794|AF(	-	monolink	monolink	318	n/a	-	F1905_sailerc	3379.805	845.959	4.000	1.9	64	22	0.56	39.34	0.000	sp|P32794|AFG2_YEAST:318
GLDATKLYVSHIQ'	sp|P05740|RL1	-	monolink	monolink	111,111	n/a	-	F1905_sailerc	2236.220	746.414	3.000	-0.7	3	21	0.60	39.30	0.000	sp|P05740|RL17A_YEAST:111:|:sp|P46990|RL17B_YEAST:111
DHGKETCTAYIHP	sp|P32794|AF(	-	monolink	monolink	46	n/a	-	F1905_sailerc	3585.726	1196.250	3.000	0.7	6	25	0.22	39.27	0.000	sp|P32794|AFG2_YEAST:46
SKLVTLAQTDKK-I	sp|P33201|MR	-	monolink	monolink	8	n/a	-	F1905_sailerc	1486.864	496.629	3.000	2.2	3	19	0.66	39.19	0.000	sp|P33201|MRT4_YEAST:8
GIAIPTTIDIDQISG	sp|Q03862|AR	-	monolink	monolink	119	n/a	-	F1905_sailerc	3936.955	1313.326	3.000	-0.5	3	25	0.25	39.18	0.000	sp|Q03862|ARX1_YEAST:119
VQEITQSVGPEKE	sp|Q08004|BU	-	monolink	monolink	124	n/a	-	F1905_sailerc	3561.837	1188.287	3.000	-1.3	9	23	0.40	39.14	0.000	sp|Q08004|BUD20_YEAST:124
FQLDRQEKEAFLE	sp|P53145|LSC	-	monolink	monolink	156	n/a	-	F1905_sailerc	2150.082	717.702	3.000	1.3	2	20	0.50	39.14	0.000	sp|P53145|LSG1_YEAST:156
LFHSIKPLFANK-KI	sp|Q02892|NC	-	monolink	monolink	275	n/a	-	F1905_sailerc	1569.894	524.306	3.000	1.4	3	22	0.58	39.01	0.000	sp|Q02892|NOG1_YEAST:275
KNESQNSTAVTLC	sp|P47019|ALE	-	monolink	monolink	63	n/a	-	F1905_sailerc	2746.379	916.468	3.000	2.1	16	27	0.36	38.97	0.000	sp|P47019|ALB1_YEAST:63
KKVMPAIVVR-K2	sp|P0CX41|RL2	-	monolink	monolink	72	n/a	-	F1905_sailerc	1295.800	432.941	3.000	0.2	2	15	0.47	38.91	0.000	sp|P0CX41|RL23A_YEAST:72
LEKADKFSVPLPK-	sp|P40078|NS/	-	monolink	monolink	114	n/a	-	F1905_sailerc	1626.929	543.318	3.000	3.7	1	17	0.67	38.89	0.000	sp|P40078|NSA2_YEAST:114
YNAFIASEVLIKQV	sp|P0CX43|RL1	-	monolink	monolink	118	n/a	-	F1905_sailerc	2003.110	668.711	3.000	0.5	3	23	0.40	38.87	0.000	sp|P0CX43|RL1A_YEAST:118
DLDLIYNDLSTKES	sp|Q08004|BU	-	monolink	monolink	28	n/a	-	F1905_sailerc	2135.106	712.710	3.000	2.9	4	25	0.66	38.84	0.000	sp|Q08004|BUD20_YEAST:28
TLASVTKEILGTAC	sp|P0CX53|RL1	-	monolink	monolink	130	n/a	-	F1905_sailerc	2146.133	716.386	3.000	1.3	5	26	0.61	38.76	0.000	sp|P0CX53|RL12A_YEAST:130
IFEFPKTTVAALR-I	sp|P0CX49|RL1	-	monolink	monolink	98	n/a	-	F1905_sailerc	1646.942	549.989	3.000	1.5	3	22	0.56	38.71	0.000	sp|P0CX49|RL18A_YEAST:98
DKNILQESVLNKYI	sp|Q03862|AR	-	monolink	monolink	27	n/a	-	F1905_sailerc	1875.012	626.012	3.000	1.1	1	20	0.49	38.69	0.000	sp|Q03862|ARX1_YEAST:27
VNNQLIKVAVHN	sp|P05738|RL9	-	monolink	monolink	54	n/a	-	F1905_sailerc	1773.004	592.009	3.000	1.6	3	24	0.56	38.56	0.000	sp|P05738|RL9A_YEAST:54
KYNLPEPLSYAAV(	sp|P32794|AF(	-	monolink	monolink	252	n/a	-	F1905_sailerc	2788.497	930.507	3.000	2.6	5	24	0.42	38.42	0.000	sp|P32794|AFG2_YEAST:252
IINSSEIQSAIRPAG	sp|P10664|RL4	-	monolink	monolink	308,308	n/a	-	F1905_sailerc	2423.315	606.837	4.000	0.8	4	18	0.71	38.33	0.000	sp|P10664|RL4A_YEAST:308:|:sp|P49626|RL4B_YEAST:308
SAEKEKPVTKK-K4	sp|P38344|REI	-	monolink	monolink	67	n/a	-	F1905_sailerc	1399.793	467.606	3.000	0.6	2	16	0.54	38.30	0.000	sp|P38344|REI1_YEAST:67
ALATESGINFLAVI	sp|P32794|AF(	-	monolink	monolink	582	n/a	-	F1905_sailerc	2374.285	792.436	3.000	2.8	11	29	0.41	38.17	0.000	sp|P32794|AFG2_YEAST:582
SCGVDAMSVDDL	sp|P0CX43|RL1	-	monolink	monolink	92	n/a	-	F1905_sailerc	1679.776	560.933	3.000	1.1	1	19	0.00	38.09	0.000	sp|P0CX43|RL1A_YEAST:92
APKWYPSEDVAA	sp|Q02326|RL(	-	monolink	monolink	8	n/a	-	F1905_sailerc	1857.006	620.010	3.000	1.1	2	19	0.47	38.09	0.000	sp|Q02326|RL6A_YEAST:8
KGLVTLETVDSYT(	sp|Q03862|AR	-	monolink	monolink	346	n/a	-	F1905_sailerc	2118.123	530.539	4.000	1.0	1	19	0.62	38.09	0.000	sp|Q03862|ARX1_YEAST:346
LPAASLGDMVMA	sp|P0CX41|RL2	-	monolink	monolink	64	n/a	-	F1905_sailerc	1785.978	596.334	3.000	2.5	1	23	0.31	38.06	0.000	sp|P0CX41|RL23A_YEAST:64
GLGTDANIDKFSLI	sp|P32794|AF(	-	monolink	monolink	485	n/a	-	F1905_sailerc	2075.156	692.726	3.000	2.0	7	20	0.48	38.05	0.000	sp|P32794|AFG2_YEAST:485
TGTKPAAVFTETLI	sp|P10664|RL4	-	monolink	monolink	360	n/a	-	F1905_sailerc	1870.972	624.665	3.000	2.0	2	19	0.50	38.04	0.000	sp|P10664|RL4A_YEAST:360
KAQVQPPYATKV	sp|P32794|AF(	-	monolink	monolink	128	n/a	-	F1905_sailerc	3435.740	859.943	4.000	-0.6	9	24	0.36	37.97	0.000	sp|P32794|AFG2_YEAST:128
YAKIHFESEAK-K3	sp|Q06338|BC	-	monolink	monolink	246	n/a	-	F1905_sailerc	1476.763	493.262	3.000	0.9	1	16	0.72	37.94	0.000	sp|Q06338|BCP1_YEAST:246
KVSSASAAAASESD	sp|P26785|RL1	-	monolink	monolink	176	n/a	-	F1905_sailerc	1661.849	554.958	3.000	1.2	2	18	0.66	37.85	0.000	sp|P26785|RL16B_YEAST:176
LATRPKCGDCGSA	sp|P87262|RL3	-	monolink	monolink	43,43	n/a	-	F1905_sailerc	2669.379	668.353	4.000	1.9	5	20	0.45	37.84	0.000	sp|P40525|RL34B_YEAST:43:|:sp|P87262|RL34A_YEAST:43
AEDEAALAKLVSTI	sp|P17076|RL8	-	monolink	monolink	213,213	n/a	-	F1905_sailerc	2981.481	746.378	4.000	1.9	9	22	0.41	37.79	0.000	sp|P17076|RL8A_YEAST:213:|:sp|P29453|RL8B_YEAST:213
KSIACVLTVINEQC	sp|P0CX84|RL3	-	monolink	monolink	49	n/a	-	F1905_sailerc	1914.031	639.018	3.000	3.4	3	24	0.59	37.77	0.000	sp|P0CX84|RL35A_YEAST:49
TGTKPAAVFTETLI	sp|P10664|RL4	-	monolink	monolink	350	n/a	-	F1905_sailerc	1869.990	624.338	3.000	3.2	3	18	0.60	37.71	0.000	sp|P10664|RL4A_YEAST:350
SIHILKVAEIEK-K6	sp|P0CX23|RL2	-	monolink	monolink	125	n/a	-	F1905_sailerc	1533.917	512.313	3.000	2.4	2	19	0.34	37.61	0.000	sp|P0CX23|RL20A_YEAST:125
QATKDAGTIAGLN	sp|P10591|HSI	-	monolink	monolink	157	n/a	-	F1905_sailerc	1782.986	595.336	3.000	1.1	2	22	0.60	37.60	0.000	sp|P10591|HSP71_YEAST:157
TLLICGYPNVGKSS	sp|Q02892|NC	-	monolink	monolink	180	n/a	-	F1905_sailerc	2079.123	694.049	3.000	1.9	2	23	0.56	37.60	0.000	sp|Q02892|NOG1_YEAST:180
ANMLENMQKSQ	sp|P38344|REI	-	monolink	monolink	105	n/a	-	F1905_sailerc	2390.118	797.714	3.000	3.7	5	25	0.37	37.55	0.000	sp|P38344|REI1_YEAST:105
VEKIPEIPLVVSTDI	sp|P10664|RL4	-	monolink	monolink	144,144	n/a	-	F1905_sailerc	2391.356	798.127	3.000	2.1	4	20	0.49	37.54	0.000	sp|P10664|RL4A_YEAST:144:|:sp|P49626|RL4B_YEAST:144
GATYGKPTNQGV	sp|P05748|RL1	-	monolink	monolink	93,93	n/a	-	F1905_sailerc	2279.162	760.729	3.000	3.4	2	23	0.57	37.42	0.000	sp|P05748|RL15A_YEAST:93:|:sp|P54780|RL15B_YEAST:93
KVLIDGPKAGVPR	sp|P36105|RL1	-	monolink	monolink	50,50	n/a	-	F1905_sailerc	1503.914	502.313	3.000	0.5	3	15	0.54	37.40	0.000	sp|P36105|RL14A_YEAST:50:|:sp|P38754|RL14B_YEAST:50
VFGALKGASDGGI	sp|P26321|RL5	-	monolink	monolink	164	n/a	-	F1905_sailerc	2328.193	777.072	3.000	2.9	5	33	0.48	37.37	0.000	sp|P26321|RL5_YEAST:164
ASGNYVIIIGHNPI	sp|P0CX45|RL2	-	monolink	monolink	145	n/a	-	F1905_sailerc	2253.144	752.056	3.000	2.0	6	24	0.46	37.36	0.000	sp|P0CX45|RL2A_YEAST:145
AVFKVKPVTR-K4-	sp|P25382|NLE	-	monolink	monolink	128	n/a	-	F1905_sailerc	1299.793	434.272	3.000	1.4	3	14	0.00	37.34	0.000	sp|P25382|NLE1_YEAST:128
LTGGSKTCQPSWI	sp|Q03862|AR	-	monolink	monolink	530	n/a	-	F1905_sailerc	4245.162	1062.298	4.000	2.1	5	23	0.45	37.31	0.000	sp|Q03862|ARX1_YEAST:530
VIQKLLDDSGVIM	sp|P32794|AF(	-	monolink	monolink	150	n/a	-	F1905_sailerc	2613.431	872.152	3.000	1.5	10	29	0.41	37.27	0.000	sp|P32794|AFG2_YEAST:150
LLGPQLSKAGKFP'	sp|P0CX43|RL1	-	monolink	monolink	130	n/a	-	F1905_sailerc	2837.504	946.843	3.000	2.8	4	22	0.32	37.23	0.000	sp|P0CX43|RL1A_YEAST:130
KSSASSNSADAKA	sp|P32794|AF(	-	monolink	monolink	24	n/a	-	F1905_sailerc	1593.786	532.270	3.000	1.0	1	19	0.24	37.17	0.000	sp|P32794|AFG2_YEAST:24
WAAAAVCEKWA.	sp|P36105|RL1	-	monolink	monolink	93	n/a	-	F1905_sailerc	2046.996	683.340	3.000	-1.1	2	21	0.61	37.14	0.000	sp|P36105|RL14A_YEAST:93
HAKQGESDRHNA	sp|Q02892|NC	-	monolink	monolink	618	n/a	-	F1905_sailerc	2019.016	505.762	4.000	1.5	5	21	0.54	37.13	0.000	sp|Q02892|NOG1_YEAST:618
SDYSRPNTKAPLTI	sp|P33201|MR	-	monolink	monolink	130	n/a	-	F1905_sailerc	2867.472	956.832	3.000	0.6	9	29	0.34	37.10	0.000	sp|P33201|MRT4_YEAST:130
VKILSIDQLEELFLS	sp|P53145|LSC	-	monolink	monolink	306	n/a	-	F1905_sailerc	2030.157	677.727	3.000	0.7	2	20	0.55	37.09	0.000	sp|P53145|LSG1_YEAST:306
NKFEIPTKIK-K8-1	sp|P33201|MR	-	monolink	monolink	174	n/a	-	F1905_sailerc	1372.797	458.607	3.000	0.7	2	14	0.43	36.99	0.000	sp|P33201|MRT4_YEAST:174
SKAQQKTAEQVA	sp|P05737|RL7	-	monolink	monolink	17	n/a	-	F1905_sailerc	1799.941	600.988	3.000	1.9	1	20	0.55	36.99	0.000	sp|P05737|RL7A_YEAST:17
KASGEIVSINQINE	sp|P0CX23|RL2	-	monolink	monolink	53	n/a	-	F1905_sailerc	2191.144	731.389	3.000	-1.9	3	24	0.48	36.98	0.000	sp|P0CX23|RL20A_YEAST:53
GIAIPTTIDIDQISG	sp|Q03862|AR	-	monolink	monolink	119	n/a	-	F1905_sailerc	3937.955	1313.660	3.000	3.7	10	29	0.23	36.92	0.000	sp|Q03862|ARX1_YEAST:119
LSHLSSNFPFVHEI	sp|Q03862|AR	-	monolink	monolink	413	n/a	-	F1905_sailerc	2866.456	956.493	3.000	2.2	6	26	0.35	36.91	0.000	sp|Q03862|ARX1_YEAST:413
APKWYPSEDVAA	sp|Q02326|RL(	-	monolink	monolink	8	n/a	-	F1905_sailerc	1857.991	620.338	3.000	1.7	4	18	0.48	36.83	0.000	sp|Q02326|RL6A_YEAST:8
LIGDAAKNQAAM	sp|P10591|HSI	-	monolink	monolink	54	n/a	-	F1905_sailerc	2431.207	811.410	3.000	0.6	2	25	0.40	36.79	0.000	sp|P10591|HSP71_YEAST:54
LSRPSLPKIK-K8-1	sp|P0CX84|RL3	-	monolink	monolink	43	n/a	-	F1905_sailerc	1292.820	431.948	3.000	1.2	6	15	0.44	36.73	0.000	sp|P0CX84|RL35A_YEAST:43
GVLLYGPPGCSKT	sp|P32794|AF(	-	monolink	monolink	568	n/a	-	F1905_sailerc	3331.816	833.962	4.000	1.9	7	20	0.67	36.55	0.000	sp|P32794|AFG2_YEAST:568
YGLNHVVSLIENKI	sp|P29453|RL8	-	monolink	monolink	146	n/a	-	F1905_sailerc	1767.991	590.338	3.000	1.3	3	23	0.48	36.49	0.000	sp|P29453|RL8B_YEAST:146
SLVDNMITGVTK(	sp|P05738|RL9	-	monolink	monolink	84,84	n/a	-	F1905_sailerc	1780.931	594.651	3.000	1.6	2	18	0.58	36.47	0.000	sp|P05738|RL9A_YEAST:84:|:sp|P51401|RL9B_YEAST:84
KFLDGIYVSHK-K1	sp|P05738|RL9	-	monolink	monolink	174,174	n/a	-	F1905_sailerc	1461.787	488.270	3.000	-0.0	2	18	0.60	36.46	0.000	sp|P05738|RL9A_YEAST:174:|:sp|P51401|RL9B_YEAST:174
DLNQLTFSAHTG(	sp|P53145|LSC	-	monolink	monolink	570	n/a	-	F1905_sailerc	2315.144	772.723	3.000	2.1	1	24	0.49	36.38	0.000	sp|P53145|LSG1_YEAST:570
GIAIPTTIDIDQISG	sp|Q03862|AR	-	monolink	monolink	121	n/a	-	F1905_sailerc	3936.963	985.249	4.000	1.6	2	24	0.28	36.31	0.000	sp|Q03862|ARX1_YEAST:121
DHGKETCTAYIHP	sp|P32794|AF(	-	monolink	monolink	46	n/a	-	F1905_sailerc	3586.710	897.685	4.000	0.8	16	26	0.35	36.28	0.000	sp|P32794|AFG2_YEAST:46
LGKGGIPNLASAG	sp|P40010|NU	-	monolink	monolink	427	n/a	-	F1905_sailerc	2293.247	765.424	3.000	1.9	2	21	0.60	36.25	0.000	sp|P40010|NUG1_YEAST:427
SKEQLASQLVDLK	sp|P0CX84|RL3	-	monolink	monolink	25	n/a	-	F1905_sailerc	1741.003	581.342	3.000	2.6	1	22	0.47	36.24	0.000	sp|P0CX84|RL35A_YEAST:25
IREGDTKDGNLR-I	sp|P53742|NO	-	monolink	monolink	18	n/a	-	F1905_sailerc	1528.786	510.603	3.000	0.9	2	16	0.55	36.23	0.000	sp|P53742|NOG2_YEAST:18
LGISAPKGVLLYGF	sp|P32794|AF(	-	monolink	monolink	551	n/a	-	F1905_sailerc	2069.125	690.716	3.000	0.9	3	23	0.53	36.14	0.000	sp|P32794|AFG2_YEAST:551
TKDSNKDVVVEGI	sp|P41805|RL1	-	monolink	monolink	145	n/a	-	F1905_sailerc	1713.929	572.318	3.000	1.9	2	20	0.74	36.10	0.000	sp|P41805|RL10_YEAST:145
CTKKFNTEESGVD	sp|P32794|AF(	-	monolink	monolink	698	n/a	-	F1905_sailerc	2504.189	835.738	3.000	1.6	1	20	0.34	36.04	0.000	sp|P32794|AFG2_YEAST:698
MASLEHCTKKGL\	sp|Q03862|AR	-	monolink	monolink	331	n/a	-	F1905_sailerc	2823.410	706.860	4.000	2.5	1	20	0.46	35.97	0.000	sp|Q03862|ARX1_YEAST:331
RGVFQKAVDAR-I	sp|P38202|YB(	-	monolink	monolink	24	n/a	-	F1905_sailerc	1401.772	468.265	3.000	-0.7	1	18	0.46	35.93	0.000	sp|P38202|YBC8_YEAST:24
AKSCGVDAMSVD	sp|P0CX43|RL1	-	monolink	monolink	78	n/a	-	F1905_sailerc	1878.909	627.311	3.000	1.3	3	21	0.28	35.91	0.000	sp|P0CX43|RL1A_YEAST:78
EIESLKSAIEIPLHQ	sp|P32794|AF(	-	monolink	monolink	258	n/a	-	F1905_sailerc	3233.742	809.443	4.000	2.6	6	21	0.49	35.72	0.000	sp|P32794|AFG2_YEAST:258
DFAQTHILKLK-K1	sp|Q03862|AR	-	monolink	monolink	373	n/a	-	F1905_sailerc	1467.848	490.290	3.000	1.8	1	15	0.33	35.72	0.000	sp|Q03862|ARX1_YEAST:373
DLKFPLPHR-K3-1	sp|P0CX23|RL2	-	monolink	monolink	149	n/a	-	F1905_sailerc	1277.714	426.912	3.000	0.2	1	13	0.00	35.70	0.000	sp|P0CX23|RL20A_YEAST:149
LPAASLGDMVMA	sp|P0CX41|RL2	-	monolink	monolink	64	n/a	-	F1905_sailerc	2467.357	617.847	4.000	1.3	1	25	0.88	35.69	0.000	sp|P0CX41|RL23A_YEAST:64
GVAMNPVDHPH	sp|P0CX45|RL2	-	monolink	monolink	221	n/a	-	F1905_sailerc	2929.437	733.367	4.000	0.1	3	20	0.58	35.68	0.000	sp|P0CX45|RL2A_YEAST:221
GILLHGPPGTGKT	sp|P32794|AF(	-	monolink	monolink	292	n/a	-	F1905_sailerc	1916.094	639.706	3.000	1.2	5	23	0.62	35.64	0.000	sp|P32794|AFG2_YEAST:292
VNNQLIKVAVHN	sp|P05738|RL9	-	monolink	monolink	54	n/a	-	F1905_sailerc	1773.987	592.337	3.000	1.3	4	17	0.60	35.63	0.000	sp|P05738|RL9A_YEAST:54
SVALDLYFQNKKN	sp|P47019|ALE	-	monolink	monolink	62	n/a	-	F1905_sailerc	4026.017	1343.013	3.000	-0.3	3	27	0.67	35.62	0.000	sp|P47019|ALB1_YEAST:62
KKEEQGIDMDVD	sp|P38202|YB(	-	monolink	monolink	52	n/a	-	F1905_sailerc	1818.858	607.294	3.000	1.4	2	20	0.40	35.61	0.000	sp|P38202|YBC8_YEAST:52
ASGNYVIIIGHNPI	sp|P0CX45|RL2	-	monolink	monolink	145	n/a	-	F1905_sailerc	2252.157	751.727	3.000	1.1	3	26	0.40	35.46	0.000	sp|P0CX45|RL2A_YEAST:145
GPEIFNKYVGESEI	sp|P32794|AF(	-	monolink	monolink	589	n/a	-	F1905_sailerc	1779.870	594.298	3.000	1.4	3	18	0.42	35.38	0.000	sp|P32794|AFG2_YEAST:589
SIVVGVIGYPNVG	sp|P40010|NU	-	monolink	monolink	293	n/a	-	F1905_sailerc	2580.504	861.176	3.000	1.4	1	22	0.47	35.30	0.000	sp|P40010|NUG1_YEAST:293
SDYSRPNTKAPLTI	sp|P33201|MR	-	monolink	monolink	130	n/a	-	F1905_sailerc	2866.488	956.504	3.000	0.5	2	26	0.35	35.30	0.000	sp|P33201|MRT4_YEAST:130
VFGALKGASDGGI	sp|P26321|RL5	-	monolink	monolink	164	n/a	-	F1905_sailerc	2329.178	777.400	3.000	3.3	2	29	0.51	35.26	0.000	sp|P26321|RL5_YEAST:164
VLEQLSGQTPVQS	sp|P0C0W9|RL	-	monolink	monolink	49,49	n/a	-	F1905_sailerc	1896.035	633.019	3.000	1.5	1	19	0.61	35.26	0.000	sp|P0C0W9|RL11A_YEAST:49:|:sp|Q3E757|RL11B_YEAST:49

IPPKPRKVLQLLR-I	sp|P05737|RL7	-	monolink	monolink	98,98	n/a	-	F1905_sailerc	1713.105	429.284	4.000	1.1	1	15	0.91	35.19	0.000	sp|P05737|RL7A_YEAST:98:|:sp|Q12213|RL7B_YEAST:98
SLMNSTKESISLPV	sp|Q03862|AR	-	monolink	monolink	493	n/a	-	F1905_sailerc	1888.000	630.341	3.000	1.2	6	26	0.58	35.19	0.000	sp|Q03862|ARX1_YEAST:493
KFNTEESGVDLHE	sp|P32794|AF	-	monolink	monolink	699	n/a	-	F1905_sailerc	2115.018	706.014	3.000	2.7	7	24	0.39	35.18	0.000	sp|P32794|AFG2_YEAST:699
SIVVGVIGYPNVGI	sp|P40010|NU	-	monolink	monolink	293	n/a	-	F1905_sailerc	2581.485	861.503	3.000	0.3	4	22	0.48	35.18	0.000	sp|P40010|NUG1_YEAST:293
GSTHITFSKETQAI	sp|P32794|AF	-	monolink	monolink	228	n/a	-	F1905_sailerc	1831.909	611.644	3.000	1.5	4	19	0.62	35.15	0.000	sp|P32794|AFG2_YEAST:228
AQRPITGASLDLIK	sp|P04449|RL2	-	monolink	monolink	91,91	n/a	-	F1905_sailerc	1922.099	641.708	3.000	2.2	2	19	0.51	35.11	0.000	sp|P04449|RL24A_YEAST:91:|:sp|P24000|RL24B_YEAST:91
LPAASLGDMVMA	sp|P0CX41|RL2	-	monolink	monolink	63	n/a	-	F1905_sailerc	1785.977	596.333	3.000	2.0	1	17	0.38	35.06	0.000	sp|P0CX41|RL23A_YEAST:63
AGKITIDSPYLVCT	sp|P33201|MR	-	monolink	monolink	179	n/a	-	F1905_sailerc	2136.069	713.031	3.000	1.5	3	20	0.38	35.00	0.000	sp|P33201|MRT4_YEAST:179
LAPELNQAIWKR-	sp|P0C2H8|RL	-	monolink	monolink	61,61	n/a	-	F1905_sailerc	1592.905	531.976	3.000	0.7	1	16	0.50	34.92	0.000	sp|P0C2H8|RL31A_YEAST:61:|:sp|P0C2H9|RL31B_YEAST:61
LATRPKCGDCGSA	sp|P87262|RL3	-	monolink	monolink	43,43	n/a	-	F1905_sailerc	2668.393	668.106	4.000	1.2	2	20	0.50	34.90	0.000	sp|P40525|RL34B_YEAST:43:|:sp|P87262|RL34A_YEAST:43
EAVAALKAVGAHS	sp|P10664|RL4	-	monolink	monolink	170,170	n/a	-	F1905_sailerc	1847.055	616.693	3.000	1.8	4	24	0.57	34.89	0.000	sp|P10664|RL4A_YEAST:170:|:sp|P49626|RL4B_YEAST:170
GFTLAEVKAAGLT	sp|Q12690|RL	-	monolink	monolink	81,81	n/a	-	F1905_sailerc	1964.074	655.699	3.000	0.5	3	17	0.49	34.84	0.000	sp|P40212|RL13B_YEAST:81:|:sp|Q12690|RL13A_YEAST:81
KGSTHITFSK-K1-1	sp|P32794|AF	-	monolink	monolink	219	n/a	-	F1905_sailerc	1260.671	421.231	3.000	-0.6	4	16	0.26	34.71	0.000	sp|P32794|AFG2_YEAST:219
AMQVHKPILLEGS	sp|Q12019|MD	-	monolink	monolink	1741	n/a	-	F1905_sailerc	2016.111	673.045	3.000	1.8	1	21	0.67	34.70	0.000	sp|Q12019|MDN1_YEAST:1741
SKAQQKTAEQVA	sp|P05737|RL7	-	monolink	monolink	21	n/a	-	F1905_sailerc	1799.940	600.988	3.000	1.4	1	20	0.66	34.63	0.000	sp|P05737|RL7A_YEAST:21
YVYAHFPINVNIVI	sp|P05738|RL9	-	monolink	monolink	106,106	n/a	-	F1905_sailerc	2331.231	778.085	3.000	1.8	2	19	0.43	34.59	0.000	sp|P05738|RL9A_YEAST:106:|:sp|P51401|RL9B_YEAST:106
LPAEFITRPHPSKD	sp|P32794|AF	-	monolink	monolink	42	n/a	-	F1905_sailerc	2084.119	695.714	3.000	1.1	4	23	0.47	34.50	0.000	sp|P32794|AFG2_YEAST:42
ISDKLKEDLLK-K6-	sp|P38202|YB	-	monolink	monolink	38	n/a	-	F1905_sailerc	1456.840	486.621	3.000	1.0	4	17	0.63	34.49	0.000	sp|P38202|YBC8_YEAST:38
LNRLPAASLGDM	sp|P0CX41|RL2	-	monolink	monolink	63	n/a	-	F1905_sailerc	2170.189	724.404	3.000	1.4	2	18	0.51	34.45	0.000	sp|P0CX41|RL23A_YEAST:63
KNESQNSTAVTLC	sp|P47019|AL	-	monolink	monolink	63	n/a	-	F1905_sailerc	2747.361	916.795	3.000	1.3	21	23	0.36	34.42	0.000	sp|P47019|ALB1_YEAST:63
VDQFPLQSTALPN	sp|Q02256|PV	-	monolink	monolink	361	n/a	-	F1905_sailerc	2918.453	973.825	3.000	1.5	5	24	0.37	34.40	0.000	sp|Q02256|PVH1_YEAST:361
TSFFQALGVPTKI	sp|P05317|RL	-	monolink	monolink	144	n/a	-	F1905_sailerc	1790.996	598.007	3.000	1.9	2	17	0.49	34.39	0.000	sp|P05317|RLA0_YEAST:144
KYQPSIIFIDEIDSI	sp|P32794|AF	-	monolink	monolink	336	n/a	-	F1905_sailerc	2374.243	792.422	3.000	0.3	8	23	0.48	34.35	0.000	sp|P32794|AFG2_YEAST:336
ALQYHPDKNPDN	sp|P53863|JJJ1	-	monolink	monolink	35	n/a	-	F1905_sailerc	2352.126	785.050	3.000	1.2	1	17	0.50	34.29	0.000	sp|P53863|JJJ1_YEAST:35
VAQLPPISFETFDS	sp|P38344|REI	-	monolink	monolink	53	n/a	-	F1905_sailerc	2706.417	903.147	3.000	1.9	1	21	0.54	34.26	0.000	sp|P38344|REI1_YEAST:53
GVLLYGPPGCSKT	sp|P32794|AF	-	monolink	monolink	563	n/a	-	F1905_sailerc	3331.822	1111.615	3.000	3.6	9	24	0.43	34.26	0.000	sp|P32794|AFG2_YEAST:563
NLKHIDVTFTK-K3	sp|P05738|RL9	-	monolink	monolink	39,39	n/a	-	F1905_sailerc	1469.825	490.950	3.000	0.9	2	15	0.37	34.24	0.000	sp|P05738|RL9A_YEAST:39:|:sp|P51401|RL9B_YEAST:39
LSHLSSNFPFVHEI	sp|Q03862|AR	-	monolink	monolink	414	n/a	-	F1905_sailerc	2867.436	717.867	4.000	0.6	1	17	0.39	34.22	0.000	sp|Q03862|ARX1_YEAST:414
QSDALEPEVKDLS	sp|P39715|EC	-	monolink	monolink	47	n/a	-	F1905_sailerc	2081.057	694.693	3.000	1.8	1	18	0.65	34.22	0.000	sp|P39715|ECM1_YEAST:47
VVANTSNAHVLTI	sp|P32794|AF	-	monolink	monolink	318	n/a	-	F1905_sailerc	3378.820	845.713	4.000	1.4	8	18	0.58	34.22	0.000	sp|P32794|AFG2_YEAST:318
DLPNVSIKFQALD	sp|P25382|NL	-	monolink	monolink	35	n/a	-	F1905_sailerc	2555.331	852.785	3.000	3.3	3	22	0.43	34.19	0.000	sp|P25382|NLE1_YEAST:35
RLPTESVPEPKLFR	sp|P0CX23|RL2	-	monolink	monolink	23	n/a	-	F1905_sailerc	1824.020	609.014	3.000	2.8	6	16	0.48	34.13	0.000	sp|P0CX23|RL20A_YEAST:23
YGLNHVVSLIENKI	sp|P29453|RL8	-	monolink	monolink	147	n/a	-	F1905_sailerc	1767.990	590.338	3.000	0.8	1	18	0.45	34.09	0.000	sp|P29453|RL8B_YEAST:147
AQKYLDQVLDHQ	sp|P46990|RL1	-	monolink	monolink	46	n/a	-	F1905_sailerc	1768.914	590.646	3.000	1.9	2	18	0.61	34.09	0.000	sp|P46990|RL17B_YEAST:46
RLPTESVPEPKLFR	sp|P0CX23|RL2	-	monolink	monolink	23	n/a	-	F1905_sailerc	1823.034	608.686	3.000	1.8	1	18	0.53	34.05	0.000	sp|P0CX23|RL20A_YEAST:23
SLTTVWAEHLSDE	sp|P14126|RL3	-	monolink	monolink	115	n/a	-	F1905_sailerc	2026.045	676.356	3.000	3.9	3	18	0.56	33.88	0.000	sp|P14126|RL3_YEAST:115
SVVSTETFEQPSQ	sp|P0C2H6|RL	-	monolink	monolink	112,112	n/a	-	F1905_sailerc	2334.170	779.065	3.000	-0.1	1	18	0.51	33.86	0.000	sp|P0C2H6|RL27A_YEAST:112:|:sp|P0C2H7|RL27B_YEAST:112
VYWSDIGGQEEL	sp|P32794|AF	-	monolink	monolink	527	n/a	-	F1905_sailerc	2167.089	723.371	3.000	0.8	3	20	0.73	33.81	0.000	sp|P32794|AFG2_YEAST:527
NPLTHSTPKNFGI	sp|P17076|RL8	-	monolink	monolink	32,32	n/a	-	F1905_sailerc	2289.212	764.079	3.000	0.3	1	20	0.52	33.76	0.000	sp|P17076|RL8A_YEAST:32:|:sp|P29453|RL8B_YEAST:32
GSTHITFSKETQAI	sp|P32794|AF	-	monolink	monolink	228	n/a	-	F1905_sailerc	1830.924	611.316	3.000	0.7	1	17	0.50	33.76	0.000	sp|P32794|AFG2_YEAST:228
TSFFQALGVPTKI	sp|P05317|RL	-	monolink	monolink	144	n/a	-	F1905_sailerc	1790.011	597.678	3.000	0.9	2	21	0.51	33.75	0.000	sp|P05317|RLA0_YEAST:144
YNLPEPLSYAAVG	sp|P32794|AF	-	monolink	monolink	252	n/a	-	F1905_sailerc	2660.408	887.810	3.000	4.8	3	19	0.57	33.70	0.000	sp|P32794|AFG2_YEAST:252
LLDDSGVIMPGM	sp|P32794|AF	-	monolink	monolink	170	n/a	-	F1905_sailerc	2374.270	792.431	3.000	2.6	1	19	0.39	33.66	0.000	sp|P32794|AFG2_YEAST:170
INNVLNKIHVAQP	sp|Q02892|NC	-	monolink	monolink	360	n/a	-	F1905_sailerc	1969.128	657.384	3.000	2.8	2	21	0.79	33.63	0.000	sp|Q02892|NOG1_YEAST:360
DFAQTHILKLK-K9	sp|Q03862|AR	-	monolink	monolink	371	n/a	-	F1905_sailerc	1468.831	490.618	3.000	1.5	2	16	0.59	33.57	0.000	sp|Q03862|ARX1_YEAST:371
KGLVTLETVDSYT	sp|Q03862|AR	-	monolink	monolink	332	n/a	-	F1905_sailerc	1764.954	589.326	3.000	1.5	1	18	0.64	33.57	0.000	sp|Q03862|ARX1_YEAST:332
KHEEDEEESAKPA	sp|Q06205|FKI	-	monolink	monolink	244	n/a	-	F1905_sailerc	1910.877	637.967	3.000	1.2	2	14	0.50	33.35	0.000	sp|Q06205|FKBP4_YEAST:244
KSIACVLTVINEQC	sp|P0CX84|RL3	-	monolink	monolink	49	n/a	-	F1905_sailerc	1913.044	638.689	3.000	2.0	1	19	0.53	33.33	0.000	sp|P0CX84|RL35A_YEAST:49
YMETAIALKTHLK-	sp|Q08004|BU	-	monolink	monolink	65	n/a	-	F1905_sailerc	1673.909	558.978	3.000	1.7	4	17	0.68	33.32	0.000	sp|Q08004|BUD20_YEAST:65
SASEGNAEKEDEE	sp|P39715|EC	-	monolink	monolink	197	n/a	-	F1905_sailerc	3904.804	1302.609	3.000	1.6	6	25	0.22	33.30	0.000	sp|P39715|ECM1_YEAST:197
TISDKNKAQLLIED	sp|Q12019|MD	-	monolink	monolink	1980	n/a	-	F1905_sailerc	2188.198	730.407	3.000	-0.6	1	19	0.52	33.30	0.000	sp|Q12019|MDN1_YEAST:1980
GVVWTESHQEAD	sp|Q03862|AR	-	monolink	monolink	285	n/a	-	F1905_sailerc	3152.568	1051.864	3.000	1.5	3	21	0.44	33.28	0.000	sp|Q03862|ARX1_YEAST:285
GFLSDLPDFEKLLP	sp|P05317|RL	-	monolink	monolink	75	n/a	-	F1905_sailerc	2119.168	707.397	3.000	3.5	1	18	0.62	33.22	0.000	sp|P05317|RLA0_YEAST:75
VAEIEKTADVKR-K	sp|P0CX23|RL2	-	monolink	monolink	131	n/a	-	F1905_sailerc	1512.853	505.292	3.000	1.3	2	17	0.56	33.11	0.000	sp|P0CX23|RL20A_YEAST:131
LFNKYRPETAAEKI	sp|P17076|RL8	-	monolink	monolink	96,96	n/a	-	F1905_sailerc	1849.996	463.507	4.000	1.0	3	18	0.68	33.10	0.000	sp|P17076|RL8A_YEAST:96:|:sp|P29453|RL8B_YEAST:96
FAKLHMGTDDVR	sp|P0C2H8|RL	-	monolink	monolink	41	n/a	-	F1905_sailerc	1544.769	515.931	3.000	1.9	1	18	0.57	33.08	0.000	sp|P0C2H8|RL31A_YEAST:41
SGTHNMYKEIR-K	sp|P0CX23|RL2	-	monolink	monolink	92	n/a	-	F1905_sailerc	1490.721	497.915	3.000	1.3	2	15	0.27	32.94	0.000	sp|P0CX23|RL20A_YEAST:92
KSEELISQDTHTG	sp|P38861|NM	-	monolink	monolink	224	n/a	-	F1905_sailerc	2150.042	717.689	3.000	2.0	3	22	0.46	32.87	0.000	sp|P38861|NMD3_YEAST:224
KAEKTGTKPAAVF	sp|P49626|RL4	-	monolink	monolink	346	n/a	-	F1905_sailerc	2045.139	512.293	4.000	-0.7	1	14	0.79	32.83	0.000	sp|P49626|RL4B_YEAST:346
INNVLNKIHVAQP	sp|Q02892|NC	-	monolink	monolink	360	n/a	-	F1905_sailerc	1970.109	657.711	3.000	1.3	3	18	0.67	32.83	0.000	sp|Q02892|NOG1_YEAST:360
SVGNLILGDRLEL	sp|P32794|AF	-	monolink	monolink	117	n/a	-	F1905_sailerc	1810.060	604.361	3.000	2.0	3	21	0.72	32.78	0.000	sp|P32794|AFG2_YEAST:117
LPAEFITRPHPSKD	sp|P32794|AF	-	monolink	monolink	42	n/a	-	F1905_sailerc	2085.104	696.043	3.000	1.6	3	20	0.80	32.64	0.000	sp|P32794|AFG2_YEAST:42
EIESLKSAIEIPLHQ	sp|P32794|AF	-	monolink	monolink	258	n/a	-	F1905_sailerc	3234.720	647.952	5.000	0.7	15	21	0.52	32.57	0.000	sp|P32794|AFG2_YEAST:258
KVWLDPNETSEIA	sp|P0CX82|RL1	-	monolink	monolink	21	n/a	-	F1905_sailerc	2212.107	738.377	3.000	-2.4	2	18	0.69	32.54	0.000	sp|P0CX82|RL19A_YEAST:21
GITEEVAKKR-K8-	sp|P04449|RL2	-	monolink	monolink	69,69	n/a	-	F1905_sailerc	1285.724	429.583	3.000	0.2	2	12	0.62	32.46	0.000	sp|P04449|RL24A_YEAST:69:|:sp|P24000|RL24B_YEAST:69
TKDSNKDVVVEGI	sp|P41805|RL1	-	monolink	monolink	145	n/a	-	F1905_sailerc	1714.912	572.645	3.000	1.1	3	14	0.74	32.37	0.000	sp|P41805|RL10_YEAST:145
AVNTGMEPGKTS	sp|P05317|RL	-	monolink	monolink	132	n/a	-	F1905_sailerc	2435.241	812.755	3.000	0.2	4	21	0.63	32.32	0.000	sp|P05317|RLA0_YEAST:132
AREKAEAEAEK-K	sp|Q12690|RL	-	monolink	monolink	190,190	n/a	-	F1905_sailerc	1386.701	463.241	3.000	1.1	2	18	0.66	32.19	0.000	sp|P40212|RL13B_YEAST:190:|:sp|Q12690|RL13A_YEAST:190
FTSKPQDENNEE	sp|P38779|CIC	-	monolink	monolink	63	n/a	-	F1905_sailerc	2191.007	731.344	3.000	2.0	2	16	0.37	32.18	0.000	sp|P38779|CIC1_YEAST:63
GHRVEKIPEIPLVV	sp|P10664|RL4	-	monolink	monolink	144,144	n/a	-	F1905_sailerc	2741.530	914.851	3.000	-0.9	4	17	0.42	32.17	0.000	sp|P10664|RL4A_YEAST:144:|:sp|P49626|RL4B_YEAST:144
VVYLKHLEDNTLL	sp|P05739|RL6	-	monolink	monolink	56	n/a	-	F1905_sailerc	2341.293	781.439	3.000	-0.3	4	17	0.85	32.01	0.000	sp|P05739|RL6B_YEAST:56

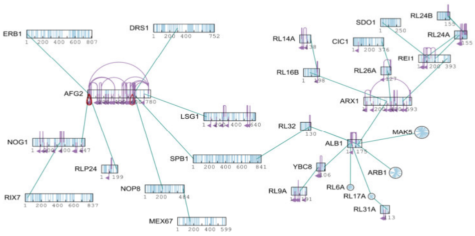

Experiment 1: Crosslinking with DSS

ID > 32

inter-protein crosslinks

Crosslinked pept	Protein1	Protein2	Type	XLType	Position 1	Position 2	deltaAA	Annotation	Mr	Mz	z	Error_rel[ppn	nseen	mions	deltaS	ld-Score	FDR	uxID
IFEFPKTTVAALR-I	sp|P0CX49|RL1	sp|P02406|RL28_YEAST	xlink	inter-protein xl	98	92	6	F1905_sailerc	2668.451	668.120	4.000	1.1	8	8	0.68	44.77	0.000	sp|P02406|RL28_YEAST:92:x:sp|P0CX49|RL18A_YEAST:98
SSVNSKSGEIK-VT	sp|P47019|AL	sp|P38061|RL32_YEAST	xlink	inter-protein xl	46	123	77	F1905_sailerc	2043.098	511.782	4.000	0.9	3	9	0.79	44.23	0.000	sp|P38061|RL32_YEAST:123:x:sp|P47019|ALB1_YEAST:46
ALKQEGAANK-AL	sp|P0CX49|RL1	sp|P05747|RL29_YEAST	xlink	inter-protein xl	72	58	14	F1905_sailerc	1838.063	613.695	3.000	0.1	8	10	0.92	43.55	0.000	sp|P05747|RL29_YEAST:58:x:sp|P0CX49|RL18A_YEAST:72
LGVSAIKLK-KGVC	sp|Q03862|AR	sp|P38344|REI1_YEAST	xlink	inter-protein xl	484	354	130	F1905_sailerc	2009.198	503.307	4.000	-0.6	8	10	0.71	42.83	0.000	sp|P38344|REI1_YEAST:354:x:sp|Q03862|ARX1_YEAST:484
AGLLKPAR-AKAL	sp|P47019|AL	sp|P38061|RL32_YEAST	xlink	inter-protein xl	37	113	76	F1905_sailerc	1662.056	416.522	4.000	0.1	2	9	0.81	42.25	0.167	sp|P38061|RL32_YEAST:113:x:sp|P47019|ALB1_YEAST:37
KLLVDASAK-AKAI	sp|P47019|AL	sp|P38061|RL32_YEAST	xlink	inter-protein xl	108	113	5	F1905_sailerc	1781.104	446.284	4.000	0.5	6	8	0.92	41.71	0.167	sp|P38061|RL32_YEAST:113:x:sp|P47019|ALB1_YEAST:108
TVKAQRPITGASLI	sp|P04449|RL2	sp|P38344|REI1_YEAST	xlink	inter-protein xl	77,77	152	75	F1905_sailerc	2850.635	571.135	5.000	-0.6	3	7	0.86	40.27	0.222	sp|P04449|RL24A_YEAST:77:|:sp|P24000|RL24B_YEAST:77:x:sp|P38344|REI1_YEAST:152
NLKYATQR-AKAL	sp|P47019|AL	sp|P38061|RL32_YEAST	xlink	inter-protein xl	102	113	11	F1905_sailerc	1830.075	458.527	4.000	1.5	4	6	0.71	40.15	0.222	sp|P38061|RL32_YEAST:113:x:sp|P47019|ALB1_YEAST:102
KVSSASAAASESD	sp|P26785|RL1	sp|P36105|RL14A_YEAST,s	xlink	inter-protein xl	176	137,137	39	F1905_sailerc	2245.218	562.312	4.000	0.7	5	3	0.62	39.35	0.222	sp|P26785|RL16B_YEAST:176:x:sp|P36105|RL14A_YEAST:137:|:sp|P38754|RL14B_YEAST:137
SLKPEVR-VMQEK	sp|P04449|RL2	sp|P38344|REI1_YEAST	xlink	inter-protein xl	97,97	152	55	F1905_sailerc	1868.057	468.022	4.000	0.7	1	6	0.95	39.07	0.222	sp|P04449|RL24A_YEAST:97:|:sp|P24000|RL24B_YEAST:97:x:sp|P38344|REI1_YEAST:152
SSVNSKSGEIK-IA	sp|P47019|AL	sp|P05740|RL17A_YEAST,s	xlink	inter-protein xl	46	180,180	134	F1905_sailerc	1958.078	490.527	4.000	-0.7	5	5	0.90	38.51	0.222	sp|P05740|RL17A_YEAST:180:|:sp|P46990|RL17B_YEAST:180:x:sp|P47019|ALB1_YEAST:46
KVSSASAAASESD	sp|P26785|RL1	sp|P36105|RL14A_YEAST,s	xlink	inter-protein xl	191	101,101	90	F1905_sailerc	3444.727	862.189	4.000	1.8	1	7	0.90	38.07	0.222	sp|P26785|RL16B_YEAST:191:x:sp|P36105|RL14A_YEAST:101:|:sp|P38754|RL14B_YEAST:101
ASKVLEQLSGQTP	sp|P0C0W9|RL	sp|P0CX43|RL1A_YEAST	xlink	inter-protein xl	35,35	78	43	F1905_sailerc	3659.881	915.978	4.000	1.7	7	8	0.49	38.04	0.222	sp|P0C0W9|RL11A_YEAST:35:|:sp|Q3E757|RL11B_YEAST:35:x:sp|P0CX43|RL1A_YEAST:78
KVSVSSTPGK-GS	sp|P53145|LSG	sp|P0CX45|RL2A_YEAST	xlink	inter-protein xl	361	251	110	F1905_sailerc	1888.976	630.667	3.000	1.1	2	11	0.80	37.66	0.222	sp|P0CX45|RL2A_YEAST:251:x:sp|P53145|LSG1_YEAST:361
IVKAGGECITLDQI	sp|P0CX49|RL1	sp|P02406|RL28_YEAST	xlink	inter-protein xl	116	92	24	F1905_sailerc	3018.607	1007.210	3.000	0.4	6	5	0.50	37.62	0.222	sp|P02406|RL28_YEAST:92:x:sp|P0CX49|RL18A_YEAST:116
SSASSNSADAKAS	sp|P32794|AF	sp|P05740|RL17A_YEAST,s	xlink	inter-protein xl	24	180,180	156	F1905_sailerc	2133.102	534.283	4.000	-0.4	2	6	0.93	37.46	0.222	sp|P05740|RL17A_YEAST:180:|:sp|P46990|RL17B_YEAST:180:x:sp|P32794|AFG2_YEAST:24
LVKALNFTNR-LPS	sp|P39715|EC	sp|P0CX45|RL2A_YEAST	xlink	inter-protein xl	115	155	40	F1905_sailerc	2012.177	671.734	3.000	-0.5	2	5	0.59	37.32	0.222	sp|P0CX45|RL2A_YEAST:155:x:sp|P39715|ECM1_YEAST:115
AARVAAKIAALAG	sp|P26321|RL5	sp|Q04660|ERB1_YEAST	xlink	inter-protein xl	289	570	281	F1905_sailerc	3016.705	755.184	4.000	3.8	1	8	0.83	36.45	0.222	sp|P26321|RL5_YEAST:289:x:sp|Q04660|ERB1_YEAST:570
KGLVTLETVDSYT	sp|Q03862|AR	sp|P47019|ALB1_YEAST	xlink	inter-protein xl	332	102	230	F1905_sailerc	2740.468	914.497	3.000	1.3	2	4	0.42	36.24	0.222	sp|P47019|ALB1_YEAST:102:x:sp|Q03862|ARX1_YEAST:332
KLLVDASAK-VTNI	sp|P47019|AL	sp|P38061|RL32_YEAST	xlink	inter-protein xl	108	123	15	F1905_sailerc	1852.079	464.028	4.000	0.7	2	8	0.81	35.95	0.222	sp|P38061|RL32_YEAST:123:x:sp|P47019|ALB1_YEAST:108
AEAKAQGVAVQL	sp|Q02753|RL	sp|P53188|CGR1_YEAST	xlink	inter-protein xl	120,120	26	94	F1905_sailerc	2462.423	616.613	4.000	0.2	2	9	0.74	35.92	0.222	sp|P53188|CGR1_YEAST:26:x:sp|Q02753|RL21A_YEAST:120:|:sp|Q12672|RL21B_YEAST:120
YGATSTNPAKSAS	sp|P05740|RL1	sp|P32794|AFG2_YEAST	xlink	inter-protein xl	13,13	13	0	F1905_sailerc	2770.339	693.593	4.000	0.9	4	16	0.71	35.65	0.222	sp|P05740|RL17A_YEAST:13:|:sp|P46990|RL17B_YEAST:13:x:sp|P32794|AFG2_YEAST:13
YGATSTNPAKSAS	sp|P05740|RL1	sp|P47019|ALB1_YEAST	xlink	inter-protein xl	13,13	37	24	F1905_sailerc	2443.319	611.837	4.000	0.1	6	8	0.63	35.60	0.222	sp|P05740|RL17A_YEAST:13:|:sp|P46990|RL17B_YEAST:13:x:sp|P47019|ALB1_YEAST:37
AGLLKPAR-VTNPI	sp|P47019|AL	sp|P38061|RL32_YEAST	xlink	inter-protein xl	37	123	86	F1905_sailerc	1733.031	434.266	4.000	-0.2	2	6	0.73	35.44	0.222	sp|P38061|RL32_YEAST:123:x:sp|P47019|ALB1_YEAST:37
KLEIQQHAR-GSQ	sp|P0CX25|RL4	sp|P0CX45|RL2A_YEAST	xlink	inter-protein xl	28	251	223	F1905_sailerc	2022.050	675.024	3.000	0.1	4	7	0.73	35.28	0.222	sp|P0CX25|RL43A_YEAST:28:x:sp|P0CX45|RL2A_YEAST:251
ASKVLEQLSGQTP	sp|P0C0W9|RL	sp|P0CX43|RL1A_YEAST	xlink	inter-protein xl	35,35	78	43	F1905_sailerc	3531.784	883.954	4.000	1.0	8	12	0.65	35.26	0.222	sp|P0C0W9|RL11A_YEAST:35:|:sp|Q3E757|RL11B_YEAST:35:x:sp|P0CX43|RL1A_YEAST:78
FAVQVDKVTK-LM	sp|P05743|RL2	sp|P25582|SPB1_YEAST	xlink	inter-protein xl	84,84	782	698	F1905_sailerc	2146.263	716.429	3.000	3.8	3	6	0.95	35.24	0.222	sp|P05743|RL26A_YEAST:84:|:sp|P53221|RL26B_YEAST:84:x:sp|P25582|SPB1_YEAST:782
KVAPAPFGAK-KA	sp|P17076|RL8	sp|P0C2H6|RL27A_YEAST,	xlink	inter-protein xl	6,6	116,116	110	F1905_sailerc	1901.040	476.268	4.000	-0.6	2	8	0.88	35.22	0.222	sp|P0C2H6|RL27A_YEAST:116:|:sp|P0C2H7|RL27B_YEAST:116:x:sp|P17076|RL8A_YEAST:6:|:sp|P29453|RL8B_YEAST:6
AKYIQNTR-GSQK	sp|P39715|EC	sp|P0CX45|RL2A_YEAST	xlink	inter-protein xl	135	251	116	F1905_sailerc	1892.960	631.995	3.000	0.6	4	6	0.71	35.01	0.222	sp|P0CX45|RL2A_YEAST:251:x:sp|P39715|ECM1_YEAST:135
NGFQTGSASKAS	sp|P49166|RL3	sp|P0CX84|RL35A_YEAST	xlink	inter-protein xl	85	71	14	F1905_sailerc	2098.071	700.365	3.000	0.7	1	3	0.89	35.00	0.222	sp|P0CX84|RL35A_YEAST:71:x:sp|P49166|RL37A_YEAST:85
KEANLFPEQQNK-	sp|Q02326|RL	sp|P47019|ALB1_YEAST	xlink	inter-protein xl	117	37	80	F1905_sailerc	2407.324	602.839	4.000	0.9	4	7	0.76	34.89	0.222	sp|P47019|ALB1_YEAST:37:x:sp|Q02326|RL6A_YEAST:117
KSSASSNSADAK-	sp|P32794|AF	sp|Q07915|RLP24_YEAST	xlink	inter-protein xl	13	184	171	F1905_sailerc	2031.121	678.048	3.000	-0.3	4	6	0.65	34.82	0.222	sp|P32794|AFG2_YEAST:13:x:sp|Q07915|RLP24_YEAST:184
KVVKEQTEAAK-S	sp|P87262|RL3	sp|P17076|RL8A_YEAST,sp	xlink	inter-protein xl	106,106	18,18	88	F1905_sailerc	2031.122	508.788	4.000	0.5	1	7	0.88	34.79	0.222	sp|P17076|RL8A_YEAST:18:|:sp|P29453|RL8B_YEAST:18:x:sp|P40525|RL34B_YEAST:106:|:sp|P87262|RL34A_YEAST:106
KGLVTLETVDSYT	sp|Q03862|AR	sp|P05743|RL26A_YEAST,s	xlink	inter-protein xl	332	84,84	248	F1905_sailerc	2881.571	721.401	4.000	0.8	4	6	0.69	34.69	0.222	sp|P05743|RL26A_YEAST:84:|:sp|P53221|RL26B_YEAST:84:x:sp|Q03862|ARX1_YEAST:332
GVVWTESHQEAD	sp|Q03862|AR	sp|P47019|ALB1_YEAST	xlink	inter-protein xl	285	137	148	F1905_sailerc	4010.093	1003.531	4.000	1.9	2	3	0.46	34.64	0.222	sp|P47019|ALB1_YEAST:137:x:sp|Q03862|ARX1_YEAST:285
TLTAKALATESGIN	sp|P32794|AF	sp|P20447|DBP3_YEAST	xlink	inter-protein xl	568	370	198	F1905_sailerc	3606.085	722.225	5.000	1.3	2	9	0.62	34.50	0.222	sp|P20447|DBP3_YEAST:370:x:sp|P32794|AFG2_YEAST:568
EEPKKEEPEQLTEE	sp|P38344|REI	sp|Q07953|SDO1_YEAST	xlink	inter-protein xl	132	25	107	F1905_sailerc	3909.840	978.468	4.000	0.1	1	2	0.44	34.35	0.231	sp|P38344|REI1_YEAST:132:x:sp|Q07953|SDO1_YEAST:25
GAVSGQKAGLIAA	sp|P0CX45|RL2	sp|P0CX41|RL23A_YEAST	xlink	inter-protein xl	234	66	168	F1905_sailerc	2390.415	598.612	4.000	1.3	1	9	0.86	34.21	0.231	sp|P0CX41|RL23A_YEAST:66:x:sp|P0CX45|RL2A_YEAST:234
KLRDPLAYLEQVR	sp|Q02892|NC	sp|P53261|PESC_YEAST	xlink	inter-protein xl	142	420	278	F1905_sailerc	2381.354	794.792	3.000	-0.3	1	3	0.62	34.17	0.231	sp|P53261|PESC_YEAST:420:x:sp|Q02892|NOG1_YEAST:142
VVKTPGGILR-ATA	sp|P87262|RL3	sp|P04456|RL25_YEAST	xlink	inter-protein xl	24,24	11	13	F1905_sailerc	1765.084	589.369	3.000	0.9	1	5	0.90	34.01	0.246	sp|P04456|RL25_YEAST:11:x:sp|P40525|RL34B_YEAST:24:|:sp|P87262|RL34A_YEAST:24
NLKYATQR-IAAQ	sp|P47019|AL	sp|P05740|RL17A_YEAST,s	xlink	inter-protein xl	102	180,180	78	F1905_sailerc	1816.034	606.353	3.000	1.4	2	5	0.76	33.87	0.246	sp|P05740|RL17A_YEAST:180:|:sp|P46990|RL17B_YEAST:180:x:sp|P47019|ALB1_YEAST:102
MKEMIQLPLEASE	sp|P32794|AF	sp|P38344|REI1_YEAST	xlink	inter-protein xl	529	200	329	F1905_sailerc	3277.756	820.447	4.000	-4.4	1	6	0.90	33.85	0.246	sp|P32794|AFG2_YEAST:529:x:sp|P38344|REI1_YEAST:200
QDKLEEKK-KPTK	sp|P43586|LO	sp|Q08208|NOP12_YEAST	xlink	inter-protein xl	154	360	206	F1905_sailerc	1982.142	661.722	3.000	-5.0	1	6	0.95	33.76	0.246	sp|P43586|LOC1_YEAST:154:x:sp|Q08208|NOP12_YEAST:360
AEAKAQGVAVQL	sp|Q02753|RL	sp|P0CX41|RL23A_YEAST	xlink	inter-protein xl	120,120	72	48	F1905_sailerc	2461.434	616.366	4.000	-4.8	1	5	0.68	33.74	0.246	sp|P0CX41|RL23A_YEAST:72:x:sp|Q02753|RL21A_YEAST:120:|:sp|Q12672|RL21B_YEAST:120
FNAWKELQK-NG	sp|P36146|LAS	sp|P38961|RRP8_YEAST	xlink	inter-protein xl	330	38	292	F1905_sailerc	1988.093	663.705	3.000	4.2	1	8	0.90	33.74	0.246	sp|P36146|LAS1_YEAST:330:x:sp|P38961|RRP8_YEAST:38
IINSSEIQSAIRPAG	sp|P10664|RL4	sp|P05737|RL7A_YEAST	xlink	inter-protein xl	308,308	17	291	F1905_sailerc	3093.691	619.746	5.000	0.6	1	3	0.70	33.52	0.246	sp|P05737|RL7A_YEAST:17:x:sp|P10664|RL4A_YEAST:308:|:sp|P49626|RL4B_YEAST:308
KVASANATAAESE	sp|P26784|RL1	sp|P36105|RL14A_YEAST,s	xlink	inter-protein xl	177	137,137	40	F1905_sailerc	2270.250	757.758	3.000	0.8	1	2	0.95	33.48	0.246	sp|P26784|RL16A_YEAST:177:x:sp|P36105|RL14A_YEAST:137:|:sp|P38754|RL14B_YEAST:137
KQLAPIQR-GTNG	sp|Q12389|DB	sp|P04456|RL25_YEAST	xlink	inter-protein xl	708	21	687	F1905_sailerc	1693.984	424.504	4.000	0.0	1	4	0.87	33.47	0.246	sp|P04456|RL25_YEAST:21:x:sp|Q12389|DBP10_YEAST:708
YVYAHFPINVNIVI	sp|P05738|RL9	sp|P38202|YBC8_YEAST	xlink	inter-protein xl	106,106	24	82	F1905_sailerc	3403.795	851.957	4.000	0.7	3	9	0.73	33.37	0.246	sp|P05738|RL9A_YEAST:106:|:sp|P51401|RL9B_YEAST:106:x:sp|P38202|YBC8_YEAST:24
GATYGKPTNQGV	sp|P05748|RL1	sp|P0CX45|RL2A_YEAST	xlink	inter-protein xl	83,83	234	151	F1905_sailerc	3111.672	778.926	4.000	1.3	4	12	0.73	33.25	0.246	sp|P05748|RL15A_YEAST:83:|:sp|P54780|RL15B_YEAST:83:x:sp|P0CX45|RL2A_YEAST:234
VVIVKPHDEGSK-I	sp|P0C2H6|RL	sp|P0CX82|RL19A_YEAST	xlink	inter-protein xl	27,27	186	159	F1905_sailerc	2318.238	580.567	4.000	0.7	1	8	0.92	33.21	0.246	sp|P0C2H6|RL27A_YEAST:27:|:sp|P0C2H7|RL27B_YEAST:27:x:sp|P0CX82|RL19A_YEAST:186
IINSSEIQSAIRPAG	sp|P10664|RL4	sp|P05737|RL7A_YEAST	xlink	inter-protein xl	308,308	21	287	F1905_sailerc	3834.039	767.816	5.000	1.2	4	14	0.56	33.19	0.246	sp|P05737|RL7A_YEAST:21:x:sp|P10664|RL4A_YEAST:308:|:sp|P49626|RL4B_YEAST:308
SSASSNSADAKAS	sp|P32794|AF	sp|Q02892|NOG1_YEAST	xlink	inter-protein xl	24	607	583	F1905_sailerc	2212.043	554.018	4.000	-1.7	2	6	0.83	33.08	0.246	sp|P32794|AFG2_YEAST:24:x:sp|Q02892|NOG1_YEAST:607
AQQKTAEQVAAE	sp|P05737|RL7	sp|P0CX49|RL18A_YEAST	xlink	inter-protein xl	21	133	112	F1905_sailerc	2776.522	695.138	4.000	0.9	2	6	0.66	32.91	0.246	sp|P05737|RL7A_YEAST:21:x:sp|P0CX49|RL18A_YEAST:133
KEANLFPEQQNK-	sp|Q02326|RL	sp|P47019|ALB1_YEAST	xlink	inter-protein xl	117	46	71	F1905_sailerc	2717.390	680.355	4.000	1.2	2	6	0.74	32.76	0.246	sp|P47019|ALB1_YEAST:46:x:sp|Q02326|RL6A_YEAST:117
RWIDEGKPEYQF	sp|Q12389|DB	sp|P38344|REI1_YEAST	xlink	inter-protein xl	577	133	444	F1905_sailerc	4001.887	801.385	5.000	-3.1	1	6	0.60	32.75	0.246	sp|P38344|REI1_YEAST:133:x:sp|Q12389|DBP10_YEAST:577
STKTFSYK-VVLIK	sp|P0CX23|RL2	sp|P36105|RL14A_YEAST,s	xlink	inter-protein xl	161	24,24	137	F1905_sailerc	1797.066	450.274	4.000	0.3	3	7	0.71	32.74	0.246	sp|P0CX23|RL20A_YEAST:161:x:sp|P36105|RL14A_YEAST:24:|:sp|P38754|RL14B_YEAST:24
KASGEIVSINQINE	sp|P0CX23|RL2	sp|P05737|RL7A_YEAST,sp	xlink	inter-protein xl	53	121,121	68	F1905_sailerc	3287.838	658.575	5.000	1.2	6	12	0.72	32.69	0.246	sp|P05737|RL7A_YEAST:121:|:sp|Q12213|RL7B_YEAST:121:x:sp|P0CX23|RL20A_YEAST:53
TLTAKALATESGIN	sp|P32794|AF	sp|Q06709|REH1_YEAST	xlink	inter-protein xl	568	196	372	F1905_sailerc	3314.805	829.709	4.000	-1.0	1	5	0.61	32.67	0.246	sp|P32794|AFG2_YEAST:568:x:sp|Q06709|REH1_YEAST:196
MKEMIQLPLEASE	sp|P32794|AF	sp|P47047|MTR4_YEAST	xlink	inter-protein xl	529	823	294	F1905_sailerc	3883.955	777.799	5.000	1.6	10	5	0.66	32.62	0.246	sp|P32794|AFG2_YEAST:529:x:sp|P47047|MTR4_YEAST:823
IGLVKYMSEK-KGI	sp|P38344|REI	sp|P04449|RL24A_YEAST,s	xlink	inter-protein xl	205	61,61	144	F1905_sailerc	2278.258	570.572	4.000	3.7	2	11	0.81	32.60	0.246	sp|P04449|RL24A_YEAST:61:|:sp|P24000|RL24B_YEAST:61:x:sp|P38344|REI1_YEAST:205
APKGQNTLILR-IL	sp|P0CX49|RL1	sp|P05737|RL7A_YEAST,sp	xlink	inter-protein xl	133	14,14	119	F1905_sailerc	2503.479	835.501	3.000	2.3	2	10	0.68	32.48	0.254	sp|P05737|RL7A_YEAST:14:|:sp|Q12213|RL7B_YEAST:14:x:sp|P0CX49|RL18A_YEAST:133
NEEEDAKNPLFSY	sp|P0C2H8|RL	sp|P05740|RL17A_YEAST,s	xlink	inter-protein xl	86,86	89,89	3	F1905_sailerc	4644.404	1162.109	4.000	1.6	2	11	0.60	32.43	0.254	sp|P05740|RL17A_YEAST:89:|:sp|P46990|RL17B_YEAST:89:x:sp|P0C2H8|RL31A_YEAST:86:|:sp|P0C2H9|RL31B_YEAST:86
VTNPKGR-VTKKP	sp|P38061|RL3	sp|P25582|SPB1_YEAST	xlink	inter-protein xl	123	786	663	F1905_sailerc	1607.973	536.999	3.000	0.1	2	7	0.93	32.37	0.256	sp|P25582|SPB1_YEAST:786:x:sp|P38061|RL32_YEAST:123
VVKEQTEAAKK-LI	sp|P87262|RL3	sp|Q06205|FKBP4_YEAST	xlink	inter-protein xl	113,113	229	116	F1905_sailerc	2583.425	517.693	5.000	-4.8	1	8	0.91	32.34	0.256	sp|P40525|RL34B_YEAST:113:|:sp|P87262|RL34A_YEAST:113:x:sp|Q06205|FKBP4_YEAST:229
KKSSASSNSADAK	sp|P32794|AF	sp|P53145|LSG1_YEAST	xlink	inter-protein xl	13	626	613	F1905_sailerc	2206.157	736.393	3.000	0.6	2	4	0.91	32.03	0.256	sp|P32794|AFG2_YEAST:13:x:sp|P53145|LSG1_YEAST:626
KGKPELRK-GSQK	sp|P0CX41|RL2	sp|P0CX45|RL2A_YEAST	xlink	inter-protein xl	66	251	185	F1905_sailerc	1855.017	464.762	4.000	0.4	1	7	0.82	32.00	0.256	sp|P0CX41|RL23A_YEAST:66:x:sp|P0CX45|RL2A_YEAST:251

intra-protein crosslinks

AEAKAQGVAVQL	sp|Q02753|RL	sp|Q02753|RL21A_YEAST,	xlink	intra/inter xl	120,120	115,115	5	F1905_sailerc	2079.182	520.803	4.000	1.1	6	6	0.72	43.88	0.000	sp|Q02753|RL21A_YEAST:115:|:sp|Q12672|RL21B_YEAST:115:x:sp|Q02753|RL21A_YEAST:120:|:sp|Q12672|RL21B_YEAST:120
VLIDGPKAGVPR-	sp|P36105|RL1	sp|P36105|RL14A_YEAST,s	xlink	intra/inter xl	50,50	83,83	33	F1905_sailerc	2092.224	524.064	4.000	-0.6	5	8	0.74	41.77	0.000	sp|P36105|RL14A_YEAST:50:|:sp|P38754|RL14B_YEAST:50:x:sp|P36105|RL14A_YEAST:83:|:sp|P38754|RL14B_YEAST:83
GAFQKVAATSR-F	sp|P04449|RL2	sp|P04449|RL24A_YEAST	xlink	intra/inter xl	149,149	140	9	F1905_sailerc	2108.139	703.721	3.000	0.7	5	8	0.85	40.88	0.000	sp|P04449|RL24A_YEAST:140:x:sp|P04449|RL24A_YEAST:149:|:sp|P24000|RL24B_YEAST:149
ASKVLEQLSGQTP	sp|P0C0W9|RL	sp|P0C0W9|RL11A_YEAST	xlink	intra/inter xl	35,35	75,75	40	F1905_sailerc	3077.665	770.424	4.000	1.3	3	8	0.53	39.31	0.000	sp|P0C0W9|RL11A_YEAST:35:|:sp|Q3E757|RL11B_YEAST:35:x:sp|P0C0W9|RL11A_YEAST:75:|:sp|Q3E757|RL11B_YEAST:75
TVKAQRPITGASLI	sp|P04449|RL2	sp|P04449|RL24A_YEAST,s	xlink	intra/inter xl	77,77	97,97	20	F1905_sailerc	2775.620	556.132	5.000	-0.7	1	10	0.74	38.64	0.000	sp|P04449|RL24A_YEAST:77:|:sp|P24000|RL24B_YEAST:77:x:sp|P04449|RL24A_YEAST:97:|:sp|P24000|RL24B_YEAST:97
TGIAIGLNKGK-KV	sp|P05745|RL3	sp|O14455|RL36B_YEAST	xlink	intra/inter xl	13,13	16	3	F1905_sailerc	2308.321	578.088	4.000	0.9	3	8	0.79	38.24	0.000	sp|O14455|RL36B_YEAST:13:|:sp|P05745|RL36A_YEAST:13:x:sp|O14455|RL36B_YEAST:16
GAFQKVAATSR-A	sp|P04449|RL2	sp|P24000|RL24B_YEAST	xlink	intra/inter xl	149,149	129	20	F1905_sailerc	2204.191	735.738	3.000	-0.1	4	9	0.88	38.23	0.000	sp|P04449|RL24A_YEAST:149:|:sp|P24000|RL24B_YEAST:149:x:sp|P24000|RL24B_YEAST:129
AKSAGTQSSK-QC	sp|P04449|RL2	sp|P04449|RL24A_YEAST,s	xlink	intra/inter xl	129	144,144	15	F1905_sailerc	2106.106	527.534	4.000	-0.3	4	9	0.86	37.97	0.000	sp|P04449|RL24A_YEAST:129:x:sp|P04449|RL24A_YEAST:144:|:sp|P24000|RL24B_YEAST:144
YGATSTNPAKSAS	sp|P05740|RL1	sp|P05740|RL17A_YEAST,s	xlink	intra/inter xl	13,13	180,180	167	F1905_sailerc	2304.219	577.062	4.000	0.0	1	5	0.84	37.91	0.000	sp|P05740|RL17A_YEAST:13:|:sp|P46990|RL17B_YEAST:13:x:sp|P05740|RL17A_YEAST:180:|:sp|P46990|RL17B_YEAST:180
SKQDASPKPYAVK	sp|P17076|RL8	sp|P17076|RL8A_YEAST	xlink	intra/inter xl	122,122	111	11	F1905_sailerc	2742.486	686.629	4.000	2.2	2	10	0.93	37.80	0.000	sp|P17076|RL8A_YEAST:111:x:sp|P17076|RL8A_YEAST:122:|:sp|P29453|RL8B_YEAST:122
GAFQKVAATSR-S	sp|P04449|RL2	sp|P04449|RL24A_YEAST	xlink	intra/inter xl	149,149	137	12	F1905_sailerc	2399.247	800.757	3.000	1.0	1	13	0.66	37.05	0.000	sp|P04449|RL24A_YEAST:137:x:sp|P04449|RL24A_YEAST:149:|:sp|P24000|RL24B_YEAST:149
GAFQKVAATSR-A	sp|P04449|RL2	sp|P04449|RL24A_YEAST	xlink	intra/inter xl	149,149	129	20	F1905_sailerc	2236.181	746.401	3.000	-0.1	3	9	0.90	36.72	0.000	sp|P04449|RL24A_YEAST:129:x:sp|P04449|RL24A_YEAST:149:|:sp|P24000|RL24B_YEAST:149
NAAYQKEYETAEF	sp|P05737|RL7	sp|P05737|RL7A_YEAST,sp	xlink	intra/inter xl	53,53	66,66	13	F1905_sailerc	2551.306	851.443	3.000	1.2	1	7	0.85	36.71	0.000	sp|P05737|RL7A_YEAST:53:|:sp|Q12213|RL7B_YEAST:53:x:sp|P05737|RL7A_YEAST:66:|:sp|Q12213|RL7B_YEAST:66
GAFQKVAATSR-A	sp|P04449|RL2	sp|P04449|RL24A_YEAST,s	xlink	intra/inter xl	149,149	120,120	29	F1905_sailerc	1917.050	640.024	3.000	3.3	2	6	0.90	36.34	0.000	sp|P04449|RL24A_YEAST:120:|:sp|P24000|RL24B_YEAST:120:x:sp|P04449|RL24A_YEAST:149:|:sp|P24000|RL24B_YEAST:149
VFAAEKLGSK-NK	sp|P10664|RL4	sp|P10664|RL4A_YEAST,sp	xlink	intra/inter xl	338,338	321,321	17	F1905_sailerc	2056.206	686.410	3.000	0.7	9	8	0.78	36.01	0.000	sp|P10664|RL4A_YEAST:321:|:sp|P49626|RL4B_YEAST:321:x:sp|P10664|RL4A_YEAST:338:|:sp|P49626|RL4B_YEAST:338
SKQDASPKPYAVK	sp|P17076|RL8	sp|P29453|RL8B_YEAST	xlink	intra/inter xl	122,122	111	11	F1905_sailerc	2756.495	690.132	4.000	-0.3	3	7	0.91	35.75	0.000	sp|P17076|RL8A_YEAST:122:|:sp|P29453|RL8B_YEAST:122:x:sp|P29453|RL8B_YEAST:111
VLLSAPLSKELR-K	sp|P05743|RL2	sp|P05743|RL26A_YEAST,s	xlink	intra/inter xl	37,37	64,64	27	F1905_sailerc	2108.225	528.064	4.000	1.9	2	5	0.86	34.50	0.013	sp|P05743|RL26A_YEAST:37:|:sp|P53221|RL26B_YEAST:37:x:sp|P05743|RL26A_YEAST:64:|:sp|P53221|RL26B_YEAST:64
VEDQKVVDK-EIK	sp|Q02326|RL	sp|P05739|RL6B_YEAST	xlink	intra/inter xl	139,139	131	8	F1905_sailerc	1971.053	493.771	4.000	0.5	2	8	0.82	34.21	0.013	sp|P05739|RL6B_YEAST:131:x:sp|P05739|RL6B_YEAST:139:|:sp|Q02326|RL6A_YEAST:139
VLIDGPKAGVPR-I	sp|P36105|RL1	sp|P36105|RL14A_YEAST	xlink	intra/inter xl	50,50	84	34	F1905_sailerc	2491.365	623.849	4.000	1.0	1	7	0.75	33.90	0.024	sp|P36105|RL14A_YEAST:50:|:sp|P38754|RL14B_YEAST:50:x:sp|P36105|RL14A_YEAST:84
LNPYAKVFAAEK-I	sp|P10664|RL4	sp|P10664|RL4A_YEAST,sp	xlink	intra/inter xl	332,332	321,321	11	F1905_sailerc	2357.348	590.345	4.000	0.2	8	9	0.79	32.90	0.028	sp|P10664|RL4A_YEAST:321:|:sp|P49626|RL4B_YEAST:321:x:sp|P10664|RL4A_YEAST:332:|:sp|P49626|RL4B_YEAST:332
KALIQR-KGGKLE-	sp|P05743|RL2	sp|P05743|RL26A_YEAST,s	xlink	intra/inter xl	116,116	125,125	9	F1905_sailerc	1495.912	499.645	3.000	2.1	2	9	0.94	32.68	0.028	sp|P05743|RL26A_YEAST:116:|:sp|P53221|RL26B_YEAST:116:x:sp|P05743|RL26A_YEAST:125:|:sp|P53221|RL26B_YEAST:125
FLKAGKVAVVVR-	sp|P0C2H6|RL	-	intralink	intralink	6,6	9,9	3	F1905_sailerc	1423.892	475.639	3.000	0.6	4	17	0.47	48.30	0.000	sp|P0C2H6|RL27A_YEAST:6:|:sp|P0C2H7|RL27B_YEAST:6:x:sp|P0C2H6|RL27A_YEAST:9:|:sp|P0C2H7|RL27B_YEAST:9
TKMKEMIQLPLEA	sp|P32794|AF	-	intralink	intralink	527	529	2	F1905_sailerc	2360.214	787.746	3.000	1.0	4	23	0.35	47.33	0.000	sp|P32794|AFG2_YEAST:527:x:sp|P32794|AFG2_YEAST:529
LALVIKSGKYTLGY	sp|P14120|RL3	-	intralink	intralink	19	22	3	F1905_sailerc	1791.055	598.026	3.000	0.3	3	20	0.49	46.65	0.000	sp|P14120|RL30_YEAST:19:x:sp|P14120|RL30_YEAST:22
SSVNSKSGEIKSVA	sp|P47019|AL	-	intralink	intralink	46	51	5	F1905_sailerc	2551.323	851.449	3.000	2.5	4	27	0.47	46.18	0.000	sp|P47019|ALB1_YEAST:46:x:sp|P47019|ALB1_YEAST:51
KLAKSMGNISQFV	sp|P38861|NM	-	intralink	intralink	262	265	3	F1905_sailerc	2048.082	683.702	3.000	1.3	2	24	0.41	46.06	0.000	sp|P38861|NMD3_YEAST:262:x:sp|P38861|NMD3_YEAST:265
KAVKELYEVDVLK	sp|P04456|RL2	-	intralink	intralink	97	100	3	F1905_sailerc	1670.949	557.991	3.000	-0.2	1	16	0.52	45.40	0.000	sp|P04456|RL25_YEAST:100:x:sp|P04456|RL25_YEAST:97
YGLNHVVALIENK	sp|P17076|RL8	-	intralink	intralink	146	147	1	F1905_sailerc	1934.100	645.708	3.000	0.6	3	22	0.40	44.87	0.000	sp|P17076|RL8A_YEAST:146:x:sp|P17076|RL8A_YEAST:147
DLMDTLYEKNHYI	sp|Q02892|NC	-	intralink	intralink	88	92	4	F1905_sailerc	2618.343	655.594	4.000	0.5	6	27	0.63	44.09	0.000	sp|Q02892|NOG1_YEAST:88:x:sp|Q02892|NOG1_YEAST:92
ETQPMKQKSVET	sp|Q03862|AR	-	intralink	intralink	576	578	2	F1905_sailerc	2575.218	859.414	3.000	1.3	7	25	0.32	43.50	0.000	sp|Q03862|ARX1_YEAST:576:x:sp|Q03862|ARX1_YEAST:578
AKIVKAGGECITLD	sp|P0CX49|RL1	-	intralink	intralink	113	116	3	F1905_sailerc	2179.208	727.410	3.000	2.0	2	23	0.46	43.31	0.000	sp|P0CX49|RL18A_YEAST:113:x:sp|P0CX49|RL18A_YEAST:116
SKAQQKTAEQVA	sp|P05737|RL7	-	intralink	intralink	17	21	4	F1905_sailerc	1781.928	594.984	3.000	0.7	3	19	0.50	43.10	0.000	sp|P05737|RL7A_YEAST:17:x:sp|P05737|RL7A_YEAST:21
ALATESGINFLAVK	sp|P32794|AF	-	intralink	intralink	582	589	7	F1905_sailerc	3176.640	1059.888	3.000	0.3	10	27	0.40	42.97	0.000	sp|P32794|AFG2_YEAST:582:x:sp|P32794|AFG2_YEAST:589
IKAGKITIDSPYLV	sp|P33201|MR	-	intralink	intralink	176	179	3	F1905_sailerc	2359.236	787.420	3.000	0.8	2	22	0.43	42.47	0.000	sp|P33201|MRT4_YEAST:176:x:sp|P33201|MRT4_YEAST:179
EHVKELLKYSNETI	sp|P0CX43|RL1	-	intralink	intralink	14	18	4	F1905_sailerc	1854.973	619.332	3.000	0.2	4	18	0.67	42.15	0.000	sp|P0CX43|RL1A_YEAST:14:x:sp|P0CX43|RL1A_YEAST:18
HFEKAFKGIAR-K4	sp|P32794|AF	-	intralink	intralink	752	755	3	F1905_sailerc	1440.788	481.271	3.000	0.3	433	14	0.49	42.10	0.000	sp|P32794|AFG2_YEAST:752:x:sp|P32794|AFG2_YEAST:755
LKEYQSKIIVFPR-K	sp|Q12690|RL	-	intralink	intralink	117,117	122,122	5	F1905_sailerc	1758.009	587.011	3.000	0.3	4	16	0.58	41.94	0.000	sp|P40212|RL13B_YEAST:117:|:sp|Q12690|RL13A_YEAST:117:x:sp|P40212|RL13B_YEAST:122:|:sp|Q12690|RL13A_YEAST:122
DFAQTHILKLKTSI	sp|Q03862|AR	-	intralink	intralink	371	373	2	F1905_sailerc	1795.002	599.342	3.000	1.7	4	21	0.67	41.93	0.000	sp|Q03862|ARX1_YEAST:371:x:sp|Q03862|ARX1_YEAST:373
GMPHKFYQGKTG	sp|Q02753|RL	-	intralink	intralink	55,55	60,60	5	F1905_sailerc	2291.179	764.734	3.000	0.8	2	19	0.41	41.60	0.000	sp|Q02753|RL21A_YEAST:55:|:sp|Q12672|RL21B_YEAST:55:x:sp|Q02753|RL21A_YEAST:60:|:sp|Q12672|RL21B_YEAST:60
HVLDSEAIKYIASK	sp|P32794|AF	-	intralink	intralink	443	448	5	F1905_sailerc	3225.657	807.422	4.000	2.3	50	24	0.55	41.49	0.000	sp|P32794|AFG2_YEAST:443:x:sp|P32794|AFG2_YEAST:448
MASLEHCTKKGL	sp|Q03862|AR	-	intralink	intralink	331	332	1	F1905_sailerc	2805.397	936.140	3.000	1.5	2	23	0.35	41.43	0.000	sp|Q03862|ARX1_YEAST:331:x:sp|Q03862|ARX1_YEAST:332
ANVTHPELGVTV	sp|P40078|NS	-	intralink	intralink	201	202	1	F1905_sailerc	4041.246	1011.319	4.000	1.9	2	26	0.39	41.31	0.000	sp|P40078|NSA2_YEAST:201:x:sp|P40078|NSA2_YEAST:202
KRPVPKGATYGKI	sp|P05748|RL1	-	intralink	intralink	72,72	77,77	5	F1905_sailerc	2519.385	630.854	4.000	-0.6	9	21	0.50	41.15	0.000	sp|P05748|RL15A_YEAST:72:|:sp|P54780|RL15B_YEAST:72:x:sp|P05748|RL15A_YEAST:77:|:sp|P54780|RL15B_YEAST:77
KAEKTGTKPAAVF	sp|P10664|RL4	-	intralink	intralink	343	350	7	F1905_sailerc	2057.140	686.721	3.000	-0.5	3	21	0.41	41.14	0.000	sp|P10664|RL4A_YEAST:343:x:sp|P10664|RL4A_YEAST:350
MAKEHKDIDASL	sp|P38861|NM	-	intralink	intralink	419	422	3	F1905_sailerc	2130.006	711.010	3.000	0.5	8	25	0.28	40.96	0.000	sp|P38861|NMD3_YEAST:419:x:sp|P38861|NMD3_YEAST:422
LPAASLGDMVMA	sp|P0CX41|RL2	-	intralink	intralink	63	64	1	F1905_sailerc	2449.352	817.459	3.000	3.5	5	28	0.72	40.66	0.000	sp|P0CX41|RL23A_YEAST:63:x:sp|P0CX41|RL23A_YEAST:64
VYWSDIGGQEEL	sp|P32794|AF	-	intralink	intralink	525	527	2	F1905_sailerc	2179.059	727.367	3.000	-0.1	4	22	0.40	40.48	0.000	sp|P32794|AFG2_YEAST:525:x:sp|P32794|AFG2_YEAST:527
DKKLVESNPELL	sp|Q07915|RL	-	intralink	intralink	124	125	1	F1905_sailerc	1708.920	570.647	3.000	-0.4	4	19	0.41	40.35	0.000	sp|Q07915|RLP24_YEAST:124:x:sp|Q07915|RLP24_YEAST:125
NTAAETFKLFNKY	sp|P17076|RL8	-	intralink	intralink	92,92	96,96	4	F1905_sailerc	2566.306	642.584	4.000	-0.4	4	17	0.52	40.34	0.000	sp|P17076|RL8A_YEAST:92:|:sp|P29453|RL8B_YEAST:92:x:sp|P17076|RL8A_YEAST:96:|:sp|P29453|RL8B_YEAST:96
KKATVDEFPLCV	sp|P41805|RL1	-	intralink	intralink	39	40	1	F1905_sailerc	3620.847	906.220	4.000	2.1	5	20	0.53	40.29	0.000	sp|P41805|RL10_YEAST:39:x:sp|P41805|RL10_YEAST:40
AFTKKVSASAASA	sp|P26785|RL1	-	intralink	intralink	175	176	1	F1905_sailerc	2092.071	698.365	3.000	1.1	2	19	0.57	40.23	0.000	sp|P26785|RL16B_YEAST:175:x:sp|P26785|RL16B_YEAST:176
AFTKKVSASAATA	sp|P26784|RL1	-	intralink	intralink	176	177	1	F1905_sailerc	2117.100	706.708	3.000	-0.1	3	19	0.57	40.21	0.000	sp|P26784|RL16A_YEAST:176:x:sp|P26784|RL16A_YEAST:177
QLLTKIDKQGVYP	sp|Q03862|AR	-	intralink	intralink	381	384	3	F1905_sailerc	1915.083	639.369	3.000	0.4	6	17	0.49	39.81	0.000	sp|Q03862|ARX1_YEAST:381:x:sp|Q03862|ARX1_YEAST:384
KSQSAGKAAIVE	sp|P36105|RL1	-	intralink	intralink	25,25	31,31	6	F1905_sailerc	2006.139	669.721	3.000	-1.1	5	21	0.46	39.29	0.000	sp|P36105|RL14A_YEAST:25:|:sp|P38754|RL14B_YEAST:25:x:sp|P36105|RL14A_YEAST:31:|:sp|P38754|RL14B_YEAST:31
AFPKDDRSKPVAL	sp|P14126|RL3	-	intralink	intralink	34	39	5	F1905_sailerc	2377.269	793.431	3.000	0.5	11	21	0.45	38.92	0.000	sp|P14126|RL3_YEAST:34:x:sp|P14126|RL3_YEAST:39
GLGTDANIDKFSL	sp|P32794|AF	-	intralink	intralink	481	485	4	F1905_sailerc	2057.144	686.722	3.000	1.3	8	20	0.72	38.82	0.000	sp|P32794|AFG2_YEAST:481:x:sp|P32794|AFG2_YEAST:485

IPKVQKLVTDYFN	sp|P10591|HS	-	intralink	intralink	342	345	3	F1905_sailerc	1887.052	630.025	3.000	0.9	2	18	0.56	38.80	0.000	sp|P10591|HSP71_YEAST:342:x:sp|P10591|HSP71_YEAST:345
APKYASKAVPHYN	sp|P04456|RL2	-	intralink	intralink	45	49	4	F1905_sailerc	1738.916	580.646	3.000	0.3	2	18	0.49	38.59	0.000	sp|P04456|RL25_YEAST:45:x:sp|P04456|RL25_YEAST:49
QGKSKLIIIAANTP	sp|P14120|RL3	-	intralink	intralink	38	40	2	F1905_sailerc	1959.191	654.071	3.000	1.4	4	21	0.55	38.44	0.000	sp|P14120|RL30_YEAST:38:x:sp|P14120|RL30_YEAST:40
SLVDNMITGVTKG	sp|P05738|RL9	-	intralink	intralink	84,84	87,87	3	F1905_sailerc	2054.078	685.700	3.000	1.0	4	21	0.43	38.29	0.000	sp|P05738|RL9A_YEAST:84:|:sp|P51401|RL9B_YEAST:84:x:sp|P05738|RL9A_YEAST:87:|:sp|P51401|RL9B_YEAST:87
YGLNHVVALIENK	sp|P17076|RL8	-	intralink	intralink	147	149	2	F1905_sailerc	1934.102	645.709	3.000	1.8	1	18	0.51	37.97	0.000	sp|P17076|RL8A_YEAST:147:x:sp|P17076|RL8A_YEAST:149
KRPVPKGATYGKF	sp|P05748|RL1	-	intralink	intralink	77,77	83,83	6	F1905_sailerc	2519.387	630.855	4.000	0.4	8	21	0.54	37.81	0.000	sp|P05748|RL15A_YEAST:77:|:sp|P54780|RL15B_YEAST:77:x:sp|P05748|RL15A_YEAST:83:|:sp|P54780|RL15B_YEAST:83
AQHVKKLATRPK-	sp|P87262|RL3	-	intralink	intralink	36,36	37,37	1	F1905_sailerc	1513.911	505.645	3.000	1.0	1	17	0.62	37.67	0.000	sp|P40525|RL34B_YEAST:36:|:sp|P87262|RL34A_YEAST:36:x:sp|P40525|RL34B_YEAST:37:|:sp|P87262|RL34A_YEAST:37
EQLASQLVDLKKE	sp|P0CX84|RL3	-	intralink	intralink	25	26	1	F1905_sailerc	2192.233	731.752	3.000	1.2	2	22	0.74	37.62	0.000	sp|P0CX84|RL35A_YEAST:25:x:sp|P0CX84|RL35A_YEAST:26
TPFIPESVKNLKK-	sp|Q02892|NO	-	intralink	intralink	383	387	4	F1905_sailerc	1637.942	546.988	3.000	1.4	3	17	0.56	37.55	0.000	sp|Q02892|NOG1_YEAST:383:x:sp|Q02892|NOG1_YEAST:387
KTVKAQRPITGAS	sp|P04449|RL2	-	intralink	intralink	74,74	77,77	3	F1905_sailerc	2076.234	693.086	3.000	1.5	5	18	0.66	37.22	0.000	sp|P04449|RL24A_YEAST:74:|:sp|P24000|RL24B_YEAST:74:x:sp|P04449|RL24A_YEAST:77:|:sp|P24000|RL24B_YEAST:77
KYTTLGKLSTSVG	sp|P26784|RL1	-	intralink	intralink	134,133	140,139	6	F1905_sailerc	1805.996	603.006	3.000	1.8	3	17	0.64	37.10	0.000	sp|P26784|RL16A_YEAST:134:|:sp|P26785|RL16B_YEAST:133:x:sp|P26784|RL16A_YEAST:140:|:sp|P26785|RL16B_YEAST:139
GVLLYGPPGCSKT	sp|P32794|AF	-	intralink	intralink	563	568	5	F1905_sailerc	3313.810	829.460	4.000	3.1	9	20	0.76	37.01	0.000	sp|P32794|AFG2_YEAST:563:x:sp|P32794|AFG2_YEAST:568
LGVSAIKLKSLMN	sp|Q03862|AR	-	intralink	intralink	484	486	2	F1905_sailerc	1827.055	610.026	3.000	0.4	3	20	0.64	36.92	0.000	sp|Q03862|ARX1_YEAST:484:x:sp|Q03862|ARX1_YEAST:486
VVLIKKGQSAGK-	sp|P36105|RL1	-	intralink	intralink	24,24	25,25	1	F1905_sailerc	1364.839	455.954	3.000	-0.5	2	17	0.29	36.84	0.000	sp|P36105|RL14A_YEAST:24:|:sp|P38754|RL14B_YEAST:24:x:sp|P36105|RL14A_YEAST:25:|:sp|P38754|RL14B_YEAST:25
SSASSNSADAKAS	sp|P32794|AF	-	intralink	intralink	24	27	3	F1905_sailerc	1722.841	575.288	3.000	-0.9	3	21	0.51	36.74	0.000	sp|P32794|AFG2_YEAST:24:x:sp|P32794|AFG2_YEAST:27
AFLIEEQKIVKK-K	sp|P87262|RL3	-	intralink	intralink	99,99	102,102	3	F1905_sailerc	1582.935	528.653	3.000	0.9	2	14	0.84	36.69	0.000	sp|P40525|RL34B_YEAST:102:|:sp|P87262|RL34A_YEAST:102:x:sp|P40525|RL34B_YEAST:99:|:sp|P87262|RL34A_YEAST:99
VVIVKPHDEGSKS	sp|P0C2H6|RL2	-	intralink	intralink	27,27	34,34	7	F1905_sailerc	2916.568	973.197	3.000	2.3	6	20	0.41	36.59	0.000	sp|P0C2H6|RL27A_YEAST:27:|:sp|P0C2H7|RL27B_YEAST:27:x:sp|P0C2H6|RL27A_YEAST:34:|:sp|P0C2H7|RL27B_YEAST:34
YGLNHVVSLIENK	sp|P29453|RL8	-	intralink	intralink	146	147	1	F1905_sailerc	1950.096	651.040	3.000	1.2	4	18	0.50	36.35	0.000	sp|P29453|RL8B_YEAST:146:x:sp|P29453|RL8B_YEAST:147
ELAELKVQKLSRP	sp|P0CX84|RL3	-	intralink	intralink	32	35	3	F1905_sailerc	2073.220	692.081	3.000	0.3	4	16	0.69	36.32	0.000	sp|P0CX84|RL35A_YEAST:32:x:sp|P0CX84|RL35A_YEAST:35
LSHLSSNFPFVHE	sp|Q03862|AR	-	intralink	intralink	413	414	1	F1905_sailerc	3205.636	1069.553	3.000	2.0	5	22	0.36	36.27	0.000	sp|Q03862|ARX1_YEAST:413:x:sp|Q03862|ARX1_YEAST:414
IGPLGLSPKKVGE	sp|P0CX53|RL1	-	intralink	intralink	40	41	1	F1905_sailerc	1859.079	620.701	3.000	1.2	3	22	0.62	36.26	0.000	sp|P0CX53|RL12A_YEAST:40:x:sp|P0CX53|RL12A_YEAST:41
AAGANVDNVWA	sp|P10622|RL	-	intralink	intralink	46	51	5	F1905_sailerc	2655.352	886.125	3.000	-0.8	3	18	0.55	36.16	0.000	sp|P10622|RLA3_YEAST:46:x:sp|P10622|RLA3_YEAST:51
SAGVQGSKVSKQ	sp|P24000|RL2	-	intralink	intralink	137	140	3	F1905_sailerc	1639.890	547.638	3.000	0.2	2	15	0.71	35.98	0.000	sp|P24000|RL24B_YEAST:137:x:sp|P24000|RL24B_YEAST:140
KPKTYKYPSLK-K3	sp|P05747|RL2	-	intralink	intralink	25	28	3	F1905_sailerc	1489.855	497.626	3.000	0.5	1	14	0.67	35.97	0.000	sp|P05747|RL29_YEAST:25:x:sp|P05747|RL29_YEAST:28
QVKKLEIQQHAR-	sp|P0CX25|RL4	-	intralink	intralink	27	28	1	F1905_sailerc	1614.918	539.314	3.000	-1.9	4	18	0.64	35.80	0.000	sp|P0CX25|RL43A_YEAST:27:x:sp|P0CX25|RL43A_YEAST:28
DKNVKHSGNIQLD	sp|P0CX53|RL1	-	intralink	intralink	96	99	3	F1905_sailerc	2429.291	810.772	3.000	-0.0	2	15	0.62	35.65	0.000	sp|P0CX53|RL12A_YEAST:96:x:sp|P0CX53|RL12A_YEAST:99
GVIGVIAGGGRVD	sp|P0CX45|RL2	-	intralink	intralink	177	181	4	F1905_sailerc	2170.295	724.440	3.000	0.0	2	22	0.48	35.64	0.000	sp|P0CX45|RL2A_YEAST:177:x:sp|P0CX45|RL2A_YEAST:181
SNKAKNPLTHSTP	sp|P29453|RL8	-	intralink	intralink	21	23	2	F1905_sailerc	1659.894	554.306	3.000	0.0	2	20	0.71	35.64	0.000	sp|P29453|RL8B_YEAST:21:x:sp|P29453|RL8B_YEAST:23
EAAAIAEGKSKQD	sp|P29453|RL8	-	intralink	intralink	120	133	13	F1905_sailerc	2396.262	799.762	3.000	1.3	4	13	0.00	35.30	0.000	sp|P29453|RL8B_YEAST:120:x:sp|P29453|RL8B_YEAST:133
KAEKTGTKPAAVF	sp|P10664|RL4	-	intralink	intralink	343	346	3	F1905_sailerc	2057.143	686.722	3.000	1.2	4	18	0.43	35.23	0.000	sp|P10664|RL4A_YEAST:343:x:sp|P10664|RL4A_YEAST:346
HKKSEELISQDTH	sp|P38861|NM	-	intralink	intralink	223	224	1	F1905_sailerc	2397.181	800.068	3.000	-0.1	2	19	0.44	35.18	0.000	sp|P38861|NMD3_YEAST:223:x:sp|P38861|NMD3_YEAST:224
SVALDLYFQNKKN	sp|P47019|AL	-	intralink	intralink	62	63	1	F1905_sailerc	4008.007	1337.010	3.000	-0.3	5	28	0.66	35.16	0.000	sp|P47019|ALB1_YEAST:62:x:sp|P47019|ALB1_YEAST:63
SAGTQSSKFSKQC	sp|P04449|RL2	-	intralink	intralink	137	140	3	F1905_sailerc	1719.882	574.302	3.000	1.5	1	14	0.80	34.90	0.000	sp|P04449|RL24A_YEAST:137:x:sp|P04449|RL24A_YEAST:140
KAEKTGTKPAAVF	sp|P10664|RL4	-	intralink	intralink	346	350	4	F1905_sailerc	2057.143	686.722	3.000	1.2	3	17	0.49	34.85	0.000	sp|P10664|RL4A_YEAST:346:x:sp|P10664|RL4A_YEAST:350
FGQSLFQCKQLKR	sp|Q02892|NO	-	intralink	intralink	126	129	3	F1905_sailerc	1776.940	593.321	3.000	3.0	1	16	0.62	34.35	0.000	sp|Q02892|NOG1_YEAST:126:x:sp|Q02892|NOG1_YEAST:129
LPAASLGDMVMA	sp|P0CX41|RL2	-	intralink	intralink	64	66	2	F1905_sailerc	2449.346	817.456	3.000	0.8	1	21	0.52	34.15	0.000	sp|P0CX41|RL23A_YEAST:64:x:sp|P0CX41|RL23A_YEAST:66
LIDVDGKPQIQVE	sp|P10591|HS	-	intralink	intralink	98	110	12	F1905_sailerc	3812.997	954.257	4.000	1.4	1	18	0.48	34.00	0.000	sp|P10591|HSP71_YEAST:110:x:sp|P10591|HSP71_YEAST:98
DQYLKSASKETAP	sp|P02406|RL2	-	intralink	intralink	92	96	4	F1905_sailerc	2663.368	888.797	3.000	-0.6	6	21	0.33	33.97	0.000	sp|P02406|RL28_YEAST:92:x:sp|P02406|RL28_YEAST:96
VAPAPFGAKSTKS	sp|P17076|RL8	-	intralink	intralink	15,15	18,18	3	F1905_sailerc	1639.896	547.640	3.000	1.3	3	16	0.63	33.62	0.000	sp|P17076|RL8A_YEAST:15:|:sp|P29453|RL8B_YEAST:15:x:sp|P17076|RL8A_YEAST:18:|:sp|P29453|RL8B_YEAST:18
TPFIPESVKNLKK-	sp|Q02892|NO	-	intralink	intralink	383	386	3	F1905_sailerc	1637.940	546.988	3.000	0.6	1	14	0.76	33.61	0.000	sp|Q02892|NOG1_YEAST:383:x:sp|Q02892|NOG1_YEAST:386
VDSVAHPAPHDK	sp|Q08208|NO	-	intralink	intralink	277	278	1	F1905_sailerc	1693.890	565.638	3.000	-0.0	4	16	0.64	33.59	0.000	sp|Q08208|NOP12_YEAST:277:x:sp|Q08208|NOP12_YEAST:278
AKNPLTHSTPKNF	sp|P29453|RL8	-	intralink	intralink	23	32	9	F1905_sailerc	2470.336	824.453	3.000	1.3	4	17	0.48	33.51	0.000	sp|P29453|RL8B_YEAST:23:x:sp|P29453|RL8B_YEAST:32
CTKKFNTEESGVD	sp|P32794|AF	-	intralink	intralink	698	699	1	F1905_sailerc	2486.177	829.734	3.000	0.9	6	21	0.33	33.36	0.000	sp|P32794|AFG2_YEAST:698:x:sp|P32794|AFG2_YEAST:699
MIDFLNAVVPIKH	sp|P38861|NM	-	intralink	intralink	221	224	3	F1905_sailerc	1890.081	631.035	3.000	0.5	2	18	0.71	33.23	0.000	sp|P38861|NMD3_YEAST:221:x:sp|P38861|NMD3_YEAST:224
LEQYYKNKVNER-	sp|Q03862|AR	-	intralink	intralink	81	83	2	F1905_sailerc	1720.879	574.634	3.000	0.3	1	13	0.68	33.14	0.000	sp|Q03862|ARX1_YEAST:81:x:sp|Q03862|ARX1_YEAST:83
QQAKGAFQKVA	sp|P04449|RL2	-	intralink	intralink	144,144	149,149	5	F1905_sailerc	1727.930	576.985	3.000	-1.1	2	13	0.83	32.96	0.000	sp|P04449|RL24A_YEAST:144:|:sp|P24000|RL24B_YEAST:144:x:sp|P04449|RL24A_YEAST:149:|:sp|P24000|RL24B_YEAST:149
KILAHLDDPDSNK	sp|P34232|MT	-	intralink	intralink	25	37	12	F1905_sailerc	3304.669	1102.564	3.000	1.0	4	16	0.40	32.90	0.000	sp|P34232|MTR2_YEAST:25:x:sp|P34232|MTR2_YEAST:37
WPAKSVKFVQGL	sp|P05740|RL1	-	intralink	intralink	86,86	89,89	3	F1905_sailerc	2578.399	860.474	3.000	3.1	6	18	0.57	32.79	0.000	sp|P05740|RL17A_YEAST:86:|:sp|P46990|RL17B_YEAST:86:x:sp|P05740|RL17A_YEAST:89:|:sp|P46990|RL17B_YEAST:89
TKDNNLLGKFELS	sp|P10591|HS	-	intralink	intralink	448	455	7	F1905_sailerc	2304.248	769.091	3.000	0.3	12	18	0.60	32.78	0.000	sp|P10591|HSP71_YEAST:448:x:sp|P10591|HSP71_YEAST:455
LPSVPECFTIEKKS	sp|Q12019|MD	-	intralink	intralink	248	249	1	F1905_sailerc	3293.680	1098.901	3.000	0.4	3	22	0.34	32.64	0.000	sp|Q12019|MDN1_YEAST:248:x:sp|Q12019|MDN1_YEAST:249
KFAKLHMGTEDV	sp|P0C2H9|RL	-	intralink	intralink	38	41	3	F1905_sailerc	1668.868	557.297	3.000	1.1	2	14	0.59	32.07	0.000	sp|P0C2H9|RL31B_YEAST:38:x:sp|P0C2H9|RL31B_YEAST:41
ASKFKLPAEFITRP	sp|P32794|AF	-	intralink	intralink	27	29	2	F1905_sailerc	2191.219	548.812	4.000	1.5	6	17	0.56	32.06	0.000	sp|P32794|AFG2_YEAST:27:x:sp|P32794|AFG2_YEAST:29
AGLLKPAR-NLKY	sp|P47019|AL	sp|P47019|ALB1_YEAST	xlink	intra-protein xl	37	102	65	F1905_sailerc	1955.131	489.791	4.000	-0.5	1	9	0.70	43.97	0.000	sp|P47019|ALB1_YEAST:102:x:sp|P47019|ALB1_YEAST:37
KVSSASAAAASESD	sp|P26785|RL1	sp|P26785|RL16B_YEAST	xlink	intra-protein xl	176	169	7	F1905_sailerc	2603.298	868.774	3.000	0.4	24	9	0.45	43.32	0.000	sp|P26785|RL16B_YEAST:169:x:sp|P26785|RL16B_YEAST:176
FDILTKQFSR-AFK	sp|P32794|AF	sp|P32794|AFG2_YEAST	xlink	intra-protein xl	425	755	330	F1905_sailerc	2153.200	539.308	4.000	-0.0	2	7	0.88	42.78	0.000	sp|P32794|AFG2_YEAST:425:x:sp|P32794|AFG2_YEAST:755
KVMPAIVVR-GKP	sp|P0CX41|RL2	sp|P0CX41|RL23A_YEAST	xlink	intra-protein xl	72	66	6	F1905_sailerc	1848.104	463.034	4.000	1.0	7	7	0.57	42.74	0.000	sp|P0CX41|RL23A_YEAST:66:x:sp|P0CX41|RL23A_YEAST:72
IVKAGGECITLDQ	sp|P0CX49|RL1	sp|P0CX49|RL18A_YEAST	xlink	intra-protein xl	116	98	18	F1905_sailerc	3471.923	868.989	4.000	1.9	6	17	0.56	42.69	0.000	sp|P0CX49|RL18A_YEAST:116:x:sp|P0CX49|RL18A_YEAST:98
TPFIPESVKNLK-K	sp|Q02892|NO	sp|Q02892|NOG1_YEAST	xlink	intra-protein xl	383	387	4	F1905_sailerc	2642.364	661.599	4.000	1.7	4	9	0.75	42.65	0.000	sp|Q02892|NOG1_YEAST:383:x:sp|Q02892|NOG1_YEAST:387
KLLVDASAK-NLK	sp|P47019|AL	sp|P47019|ALB1_YEAST	xlink	intra-protein xl	108	102	6	F1905_sailerc	2074.181	519.553	4.000	1.1	3	12	0.72	42.12	0.000	sp|P47019|ALB1_YEAST:102:x:sp|P47019|ALB1_YEAST:108
AKSAGTQSSK-FS	sp|P04449|RL2	sp|P04449|RL24A_YEAST	xlink	intra-protein xl	129	140	11	F1905_sailerc	1937.021	485.263	4.000	-0.2	7	7	0.87	41.56	0.000	sp|P04449|RL24A_YEAST:129:x:sp|P04449|RL24A_YEAST:140
GSQKTQD-LPSGA	sp|P0CX45|RL2	sp|P0CX45|RL2A_YEAST	xlink	intra-protein xl	251	155	96	F1905_sailerc	1599.847	534.290	3.000	0.4	5	9	0.86	41.51	0.000	sp|P0CX45|RL2A_YEAST:155:x:sp|P0CX45|RL2A_YEAST:251
AEKTDLTNILIR-T	sp|P39715|EC	sp|P39715|ECM1_YEAST	xlink	intra-protein xl	57	70	13	F1905_sailerc	2781.552	696.396	4.000	1.1	4	12	0.85	41.40	0.000	sp|P39715|ECM1_YEAST:57:x:sp|P39715|ECM1_YEAST:70
VAAKIAALAGQQ-	sp|P26321|RL5	sp|P26321|RL5_YEAST	xlink	intra-protein xl	289	8	281	F1905_sailerc	2348.236	783.753	3.000	0.9	3	8	0.65	41.35	0.000	sp|P26321|RL5_YEAST:289:x:sp|P26321|RL5_YEAST:8
LWDPKSGQCLGD	sp|P25382|NL	sp|P25382|NLE1_YEAST	xlink	intra-protein xl	216	345	129	F1905_sailerc	2966.495	742.631	4.000	4.7	1	10	0.76	40.89	0.000	sp|P25382|NLE1_YEAST:216:x:sp|P25382|NLE1_YEAST:345
KLLEIAR-VMQEK	sp|P38344|RE	sp|P38344|REI1_YEAST	xlink	intra-protein xl	90	152	62	F1905_sailerc	1882.106	471.534	4.000	-0.8	2	10	0.86	40.74	0.000	sp|P38344|REI1_YEAST:152:x:sp|P38344|REI1_YEAST:90
INNVLNKIHVAQP	sp|Q02892|NO	sp|Q02892|NOG1_YEAST	xlink	intra-protein xl	360	349	11	F1905_sailerc	2669.509	534.910	5.000	0.1	4	6	0.90	40.44	0.000	sp|Q02892|NOG1_YEAST:349:x:sp|Q02892|NOG1_YEAST:360
LVKALNFTNR-AK	sp|P39715|EC	sp|P39715|ECM1_YEAST	xlink	intra-protein xl	115	135	20	F1905_sailerc	2305.290	577.330	4.000	-0.2	5	9	0.75	40.26	0.000	sp|P39715|ECM1_YEAST:115:x:sp|P39715|ECM1_YEAST:135
SSVNSKSGEIK-AG	sp|P47019|AL	sp|P47019|ALB1_YEAST	xlink	intra-protein xl	46	37	9	F1905_sailerc	2097.182	525.303	4.000	1.4	9	10	0.77	40.25	0.000	sp|P47019|ALB1_YEAST:37:x:sp|P47019|ALB1_YEAST:46
SSASSNSADAKAS	sp|P32794|AF	sp|P32794|AFG2_YEAST	xlink	intra-protein xl	24	24	0	F1905_sailerc	2757.291	690.331	4.000	0.5	6	19	0.65	40.21	0.000	sp|P32794|AFG2_YEAST:24:x:sp|P32794|AFG2_YEAST:24
IGPLGLSPKK-ATK	sp|P0CX53|RL1	sp|P0CX53|RL12A_YEAST	xlink	intra-protein xl	40	51	11	F1905_sailerc	1869.098	468.282	4.000	-0.0	5	7	0.72	39.82	0.000	sp|P0CX53|RL12A_YEAST:40:x:sp|P0CX53|RL12A_YEAST:51
GLGTDANIDKFSL	sp|P32794|AF	sp|P32794|AFG2_YEAST	xlink	intra-protein xl	481	425	56	F1905_sailerc	2869.527	718.389	4.000	1.4	4	13	0.62	39.77	0.000	sp|P32794|AFG2_YEAST:425:x:sp|P32794|AFG2_YEAST:481
YGLNHVVSLIENK	sp|P29453|RL8	sp|P29453|RL8B_YEAST	xlink	intra-protein xl	146	172	26	F1905_sailerc	2869.615	574.931	5.000	0.3	3	15	0.71	38.72	0.000	sp|P29453|RL8B_YEAST:146:x:sp|P29453|RL8B_YEAST:172
KELAFLNGGLSVQ	sp|P39715|EC	sp|P39715|ECM1_YEAST	xlink	intra-protein xl	154	135	19	F1905_sailerc	2704.497	677.132	4.000	2.0	1	7	0.67	38.51	0.000	sp|P39715|ECM1_YEAST:135:x:sp|P39715|ECM1_YEAST:154
AKALGIK-VTNPK	sp|P38061|RL3	sp|P38061|RL32_YEAST	xlink	intra-protein xl	113	123	10	F1905_sailerc	1607.972	403.001	4.000	-0.3	6	9	0.86	38.44	0.000	sp|P38061|RL32_YEAST:113:x:sp|P38061|RL32_YEAST:123
GAVSGQKAGLIA	sp|P0CX45|RL2	sp|P0CX45|RL2A_YEAST	xlink	intra-protein xl	234	251	17	F1905_sailerc	2198.171	733.732	3.000	2.5	3	7	0.88	37.93	0.000	sp|P0CX45|RL2A_YEAST:234:x:sp|P0CX45|RL2A_YEAST:251
MKEMIQLPLEASE	sp|P32794|AF	sp|P32794|AFG2_YEAST	xlink	intra-protein xl	529	525	4	F1905_sailerc	3882.944	777.597	5.000	0.3	26	10	0.84	37.87	0.000	sp|P32794|AFG2_YEAST:525:x:sp|P32794|AFG2_YEAST:529
ICANKYMTTVSGF	sp|P41805|RL1	sp|P41805|RL10_YEAST	xlink	intra-protein xl	74	158	84	F1905_sailerc	2632.315	659.087	4.000	0.5	4	8	0.76	37.86	0.000	sp|P41805|RL10_YEAST:158:x:sp|P41805|RL10_YEAST:74
VGKGDDEANGAT	sp|P14126|RL3	sp|P14126|RL3_YEAST	xlink	intra-protein xl	287	168	119	F1905_sailerc	2560.278	854.434	3.000	0.2	6	7	0.43	37.71	0.000	sp|P14126|RL3_YEAST:168:x:sp|P14126|RL3_YEAST:287
MKEMIQLPLEASE	sp|P32794|AF	sp|P32794|AFG2_YEAST	xlink	intra-protein xl	529	755	226	F1905_sailerc	2892.529	724.140	4.000	1.7	2	7	0.54	37.59	0.000	sp|P32794|AFG2_YEAST:529:x:sp|P32794|AFG2_YEAST:755
GSSSLYTLVINDAC	sp|P49167|RL3	sp|P49167|RL38_YEAST	xlink	intra-protein xl	61	9	52	F1905_sailerc	3465.865	867.474	4.000	1.3	6	9	0.50	37.59	0.000	sp|P49167|RL38_YEAST:61:x:sp|P49167|RL38_YEAST:9
GSSSLYTLVINDAC	sp|P49167|RL3	sp|P49167|RL38_YEAST	xlink	intra-protein xl	61	64	3	F1905_sailerc	3097.771	775.451	4.000	2.3	43	12	0.67	37.47	0.000	sp|P49167|RL38_YEAST:61:x:sp|P49167|RL38_YEAST:64
SSASSNSADAKAS	sp|P32794|AF	sp|P32794|AFG2_YEAST	xlink	intra-protein xl	24	13	11	F1905_sailerc	2727.316	682.837	4.000	-0.1	1	13	0.71	36.86	0.000	sp|P32794|AFG2_YEAST:13:x:sp|P32794|AFG2_YEAST:24
EKALNEEAEAR-D	sp|P0CX82|RL1	sp|P0CX82|RL19A_YEAST	xlink	intra-protein xl	153	186	33	F1905_sailerc	2270.135	757.719	3.000	3.1	4	8	0.59	36.82	0.000	sp|P0CX82|RL19A_YEAST:153:x:sp|P0CX82|RL19A_YEAST:186
TLASVTKEILGTAC	sp|P0CX53|RL1	sp|P0CX53|RL12A_YEAST	xlink	intra-protein xl	130	119	11	F1905_sailerc	2836.470	710.125	4.000	-1.8	1	5	0.90	36.66	0.000	sp|P0CX53|RL12A_YEAST:119:x:sp|P0CX53|RL12A_YEAST:130
KLLVDASAK-AGL	sp|P47019|AL	sp|P47019|ALB1_YEAST	xlink	intra-protein xl	108	37	71	F1905_sailerc	1906.160	477.548	4.000	-0.9	3	11	0.80	36.63	0.000	sp|P47019|ALB1_YEAST:108:x:sp|P47019|ALB1_YEAST:37
KSSASSNSADAK-	sp|P32794|AF	sp|P32794|AFG2_YEAST	xlink	intra-protein xl	13	755	742	F1905_sailerc	2051.064	513.774	4.000	-0.4	1	9	0.89	36.60	0.000	sp|P32794|AFG2_YEAST:13:x:sp|P32794|AFG2_YEAST:755
KVWLDPNETSEIA	sp|P0CX82|RL1	sp|P0CX82|RL19A_YEAST	xlink	intra-protein xl	21	19	2	F1905_sailerc	3250.738	813.692	4.000	2.0	5	6	0.85	36.57	0.000	sp|P0CX82|RL19A_YEAST:19:x:sp|P0CX82|RL19A_YEAST:21
GLGTDANIDKFSL	sp|P32794|AF	sp|P32794|AFG2_YEAST	xlink	intra-protein xl	481	467	14	F1905_sailerc	2706.430	677.615	4.000	1.4	5	10	0.73	36.50	0.000	sp|P32794|AFG2_YEAST:467:x:sp|P32794|AFG2_YEAST:481
TLTAKALATESGIN	sp|P32794|AF	sp|P32794|AFG2_YEAST	xlink	intra-protein xl	568	589	21	F1905_sailerc	3708.969	928.250	4.000	2.1	5	7	0.87	36.12	0.000	sp|P32794|AFG2_YEAST:568:x:sp|P32794|AFG2_YEAST:589
GPEIFNKYVGESE	sp|P32794|AF	sp|P32794|AFG2_YEAST	xlink	intra-protein xl	589	589	0	F1905_sailerc	3385.649	1129.557	3.000	0.6	4	16	0.71	35.78	0.000	sp|P32794|AFG2_YEAST:589:x:sp|P32794|AFG2_YEAST:589
AVVKGTNGK-AT	sp|P04456|RL2	sp|P04456|RL25_YEAST	xlink	intra-protein xl	16	11	5	F1905_sailerc	1598.935	533.986	3.000	-0.1	3	5	0.93	35.74	0.000	sp|P04456|RL25_YEAST:11:x:sp|P04456|RL25_YEAST:16
GLGTDANIDKFSL	sp|P32794|AF	sp|P32794|AFG2_YEAST	xlink	intra-protein xl	481	13	468	F1905_sailerc	2767.400	692.858	4.000	4.4	2	11	0.73	35.69	0.000	sp|P32794|AFG2_YEAST:13:x:sp|P32794|AFG2_YEAST:481
QSVNLYKNR-AQ	sp|P38861|NM	sp|P38861|NMD3_YEAST	xlink	intra-protein xl	467	436	31	F1905_sailerc	2277.152	570.296	4.000	-0.7	4	9	0.81	35.64	0.000	sp|P38861|NMD3_YEAST:436:x:sp|P38861|NMD3_YEAST:467
KVASANATAAES	sp|P26784|RL1	sp|P26784|RL16A_YEAST	xlink	intra-protein xl	177	170	7	F1905_sailerc	2814.429	939.151	3.000	0.2	6	6	0.46	35.45	0.000	sp|P26784|RL16A_YEAST:170:x:sp|P26784|RL16A_YEAST:177
IENKLK-NKACEK-	sp|Q02892|NO	sp|Q02892|NOG1_YEAST	xlink	intra-protein xl	347	334	13	F1905_sailerc	1629.875	408.477	4.000	-0.6	1	8	0.93	35.23	0.013	sp|Q02892|NOG1_YEAST:334:x:sp|Q02892|NOG1_YEAST:347
IFEFPKTTVAALR-	sp|P0CX49|RL1	sp|P0CX49|RL18A_YEAST	xlink	intra-protein xl	98	72	26	F1905_sailerc	2658.484	665.629	4.000	3.6	2	9	0.78	35.22	0.013	sp|P0CX49|RL18A_YEAST:72:x:sp|P0CX49|RL18A_YEAST:98
SSASSNSADAKAS	sp|P32794|AF	sp|P32794|AFG2_YEAST	xlink	intra-protein xl	24	12	12	F1905_sailerc	2727.316	546.471	5.000	-0.1	2	12	0.74	35.09	0.013	sp|P32794|AFG2_YEAST:12:x:sp|P32794|AFG2_YEAST:24
IGLVKYMSEK-VM	sp|P38344|RE	sp|P38344|REI1_YEAST	xlink	intra-protein xl	205	152	53	F1905_sailerc	2207.206	552.809	4.000	0.3	6	10	0.82	35.05	0.013	sp|P38344|REI1_YEAST:152:x:sp|P38344|REI1_YEAST:205
QKSVETSNGGVE	sp|Q03862|AR	sp|Q03862|ARX1_YEAST	xlink	intra-protein xl	578	83	495	F1905_sailerc	2619.282	655.828	4.000	0.3	7	5	0.83	34.77	0.013	sp|Q03862|ARX1_YEAST:578:x:sp|Q03862|ARX1_YEAST:83
ANQLLEKQK-KEQ	sp|P53145|LS	sp|P53145|LSG1_YEAST	xlink	intra-protein xl	274	534	260	F1905_sailerc	2223.225	742.083	3.000	1.3	2	9	0.81	34.69	0.013	sp|P53145|LSG1_YEAST:274:x:sp|P53145|LSG1_YEAST:534
NILQESVLNKYR-K	sp|Q03862|AR	sp|Q03862|ARX1_YEAST	xlink	intra-protein xl	27	83	56	F1905_sailerc	2372.283	791.769	3.000	0.9	1	5	0.85	34.66	0.013	sp|Q03862|ARX1_YEAST:27:x:sp|Q03862|ARX1_YEAST:83
AKSAGVQGSK-VS	sp|P24000|RL2	sp|P24000|RL24B_YEAST	xlink	intra-protein xl	129	140	11	F1905_sailerc	1857.034	620.019	3.000	1.3	3	9	0.82	34.66	0.013	sp|P24000|RL24B_YEAST:129:x:sp|P24000|RL24B_YEAST:140
SKPVALTSFLGYK-	sp|P14126|RL3	sp|P14126|RL3_YEAST	xlink	intra-protein xl	39	349	310	F1905_sailerc	2563.454	641.871	4.000	0.9	2	5	0.92	34.65	0.013	sp|P14126|RL3_YEAST:349:x:sp|P14126|RL3_YEAST:39
YIASKTHGYVGAD	sp|P32794|AF	sp|P32794|AFG2_YEAST	xlink	intra-protein xl	448	529	81	F1905_sailerc	4226.119	846.232	5.000	-0.5	5	7	0.84	34.58	0.013	sp|P32794|AFG2_YEAST:448:x:sp|P32794|AFG2_YEAST:529
VGEDIAKATK-EF	sp|P0CX53|RL1	sp|P0CX53|RL12A_YEAST	xlink	intra-protein xl	48	54	6	F1905_sailerc	1889.052	473.271	4.000	0.4	4	7	0.86	34.54	0.013	sp|P0CX53|RL12A_YEAST:48:x:sp|P0CX53|RL12A_YEAST:54
LGISAPKGVLLYG	sp|P32794|AF	sp|P32794|AFG2_YEAST	xlink	intra-protein xl	551	589	38	F1905_sailerc	3674.908	919.735	4.000	1.7	2	10	0.64	34.37	0.013	sp|P32794|AFG2_YEAST:551:x:sp|P32794|AFG2_YEAST:589
QSDALEPEVKDLS	sp|P39715|EC	sp|P39715|ECM1_YEAST	xlink	intra-protein xl	47	57	10	F1905_sailerc	3448.833	863.216	4.000	0.9	5	7	0.83	34.33	0.013	sp|P39715|ECM1_YEAST:47:x:sp|P39715|ECM1_YEAST:57
KYNLPEPLSYAAV	sp|P32794|AF	sp|P32794|AFG2_YEAST	xlink	intra-protein xl	235	481	246	F1905_sailerc	3549.860	888.473	4.000	-0.2	4	4	0.54	34.23	0.013	sp|P32794|AFG2_YEAST:235:x:sp|P32794|AFG2_YEAST:481
SSASSNSADAKAS	sp|P32794|AF	sp|P32794|AFG2_YEAST	xlink	intra-protein xl	24	118	94	F1905_sailerc	2677.359	670.347	4.000	1.0	20	10	0.75	34.16	0.013	sp|P32794|AFG2_YEAST:118:x:sp|P32794|AFG2_YEAST:24
ANMLENMQKSQ	sp|P38344|RE	sp|P38344|REI1_YEAST	xlink	intra-protein xl	105	152	47	F1905_sailerc	3274.602	819.658	4.000	0.9	2	4	0.61	34.09	0.013	sp|P38344|REI1_YEAST:105:x:sp|P38344|REI1_YEAST:152
KLVESNPELLR-EK	sp|Q07915|RL	sp|Q07915|RLP24_YEAST	xlink	intra-protein xl	125	118	7	F1905_sailerc	2241.240	561.318	4.000	1.1	6	5	0.77	34.01	0.013	sp|Q07915|RLP24_YEAST:118:x:sp|Q07915|RLP24_YEAST:125
KNESQNSTAVTLC	sp|P47019|AL	sp|P47019|ALB1_YEAST	xlink	intra-protein xl	63	108	45	F1905_sailerc	3672.916	1225.313	3.000	-0.4	5	3	0.90	33.95	0.024	sp|P47019|ALB1_YEAST:108:x:sp|P47019|ALB1_YEAST:63
VGKGDDEANGAT	sp|P14126|RL3	sp|P14126|RL3_YEAST	xlink	intra-protein xl	287	168	119	F1905_sailerc	2688.375	673.102	4.000	1.1	5	8	0.63	33.84	0.024	sp|P14126|RL3_YEAST:168:x:sp|P14126|RL3_YEAST:287
AVVKGTNGKK-AT	sp|P04456|RL2	sp|P04456|RL25_YEAST	xlink	intra-protein xl	16	11	5	F1905_sailerc	1727.031	576.685	3.000	0.2	1	6	0.72	33.62	0.024	sp|P04456|RL25_YEAST:11:x:sp|P04456|RL25_YEAST:16
SASKETAPVIDTLA	sp|P02406|RL2	sp|P02406|RL28_YEAST	xlink	intra-protein xl	96	86	10	F1905_sailerc	3285.756	822.447	4.000	1.9	7	6	0.74	33.60	0.024	sp|P02406|RL28_YEAST:86:x:sp|P02406|RL28_YEAST:96
FDILTKQFSR-ESV	sp|P32794|AF	sp|P32794|AFG2_YEAST	xlink	intra-protein xl	425	467	42	F1905_sailerc	2482.329	621.590	4.000	1.4	2	9	0.70	33.45	0.024	sp|P32794|AFG2_YEAST:425:x:sp|P32794|AFG2_YEAST:467
KSSLTLVK-VKENE	sp|P47019|AL	sp|P47019|ALB1_YEAST	xlink	intra-protein xl	137	132	5	F1905_sailerc	1758.015	587.013	3.000	0.7	4	8	0.86	33.39	0.024	sp|P47019|ALB1_YEAST:132:x:sp|P47019|ALB1_YEAST:137
GVLLYGPPGCSKT	sp|P32794|AF	sp|P32794|AFG2_YEAST	xlink	intra-protein xl	563	467	96	F1905_sailerc	2989.599	748.407	4.000	0.2	2	6	0.74	33.28	0.024	sp|P32794|AFG2_YEAST:467:x:sp|P32794|AFG2_YEAST:563
VYWSDIGGQEEL	sp|P32794|AF	sp|P32794|AFG2_YEAST	xlink	intra-protein xl	525	755	230	F1905_sailerc	2910.535	583.115	5.000	1.3	1	7	0.73	33.16	0.028	sp|P32794|AFG2_YEAST:525:x:sp|P32794|AFG2_YEAST:755
NKLELGR-AIKYAR	sp|P53145|LS	sp|P53145|LSG1_YEAST	xlink	intra-protein xl	26	34	8	F1905_sailerc	1686.977	422.752	4.000	-0.5	1	4	0.84	33.12	0.028	sp|P53145|LSG1_YEAST:26:x:sp|P53145|LSG1_YEAST:34
VSAAAASTSKSAE	sp|P38344|RE	sp|P38344|REI1_YEAST	xlink	intra-protein xl	63	90	27	F1905_sailerc	2286.280	572.578	4.000	0.3	2	6	0.83	33.08	0.028	sp|P38344|REI1_YEAST:63:x:sp|P38344|REI1_YEAST:90
QATKDAGTIAGLN	sp|P10591|HS	sp|P10591|HSP71_YEAST	xlink	intra-protein xl	157	509	352	F1905_sailerc	2725.495	909.506	3.000	3.2	4	4	0.52	32.95	0.028	sp|P10591|HSP71_YEAST:157:x:sp|P10591|HSP71_YEAST:509
SSVNSKSGEIK-KL	sp|P47019|AL	sp|P47019|ALB1_YEAST	xlink	intra-protein xl	46	108	62	F1905_sailerc	2216.226	739.750	3.000	-0.1	1	9	0.87	32.87	0.028	sp|P47019|ALB1_YEAST:108:x:sp|P47019|ALB1_YEAST:46
EITDIKQFLELTR-K	sp|P49167|RL3	sp|P49167|RL38_YEAST	xlink	intra-protein xl	9	64	55	F1905_sailerc	2979.728	745.940	4.000	0.7	9	11	0.69	32.87	0.028	sp|P49167|RL38_YEAST:64:x:sp|P49167|RL38_YEAST:9
KSSASSNSADAK-	sp|P32794|AF	sp|P32794|AFG2_YEAST	xlink	intra-protein xl	13	467	454	F1905_sailerc	2380.194	596.056	4.000	1.6	2	8	0.76	32.82	0.028	sp|P32794|AFG2_YEAST:13:x:sp|P32794|AFG2_YEAST:467
IAWAKYFISK-ADL	sp|P53145|LS	sp|P53145|LSG1_YEAST	xlink	intra-protein xl	251	243	8	F1905_sailerc	2151.235	538.817	4.000	0.4	1	6	0.93	32.76	0.028	sp|P53145|LSG1_YEAST:243:x:sp|P53145|LSG1_YEAST:251
SSASSNSADAKAS	sp|P32794|AF	sp|P32794|AFG2_YEAST	xlink	intra-protein xl	24	13	11	F1905_sailerc	2599.219	867.414	3.000	-0.6	1	9	0.66	32.72	0.028	sp|P32794|AFG2_YEAST:13:x:sp|P32794|AFG2_YEAST:24
VRLPSGAKK-GSQ	sp|P0CX45|RL2	sp|P0CX45|RL2A_YEAST	xlink	intra-protein xl	155	251	96	F1905_sailerc	1855.017	619.347	3.000	0.3	3	7	0.94	32.72	0.028	sp|P0CX45|RL2A_YEAST:155:x:sp|P0CX45|RL2A_YEAST:251
ACEKLLASR-LKSQ	sp|Q02892|NO	sp|Q02892|NOG1_YEAST	xlink	intra-protein xl	338	349	11	F1905_sailerc	1902.029	635.018	3.000	-3.4	3	5	0.94	32.66	0.028	sp|Q02892|NOG1_YEAST:338:x:sp|Q02892|NOG1_YEAST:349
KNESQNSTAVTLC	sp|P47019|AL	sp|P47019|ALB1_YEAST	xlink	intra-protein xl	63	46	17	F1905_sailerc	3863.933	773.794	5.000	-0.5	10	8	0.49	32.65	0.028	sp|P47019|ALB1_YEAST:46:x:sp|P47019|ALB1_YEAST:63
ANQLLEKQK-ADL	sp|P53145|LS	sp|P53145|LSG1_YEAST	xlink	intra-protein xl	274	243	31	F1905_sailerc	1996.155	500.047	4.000	-0.9	2	9	0.89	32.41	0.028	sp|P53145|LSG1_YEAST:243:x:sp|P53145|LSG1_YEAST:274
ALVEHIIQAKADA	sp|P0CX82|RL1	sp|P0CX82|RL19A_YEAST	xlink	intra-protein xl	146	153	7	F1905_sailerc	3058.608	765.660	4.000	0.9	2	9	0.75	32.32	0.028	sp|P0CX82|RL19A_YEAST:146:x:sp|P0CX82|RL19A_YEAST:153
LGISAPKGVLLYG	sp|P32794|AF	sp|P32794|AFG2_YEAST	xlink	intra-protein xl	551	568	17	F1905_sailerc	3998.221	800.652	5.000	1.2	6	13	0.71	32.25	0.028	sp|P32794|AFG2_YEAST:551:x:sp|P32794|AFG2_YEAST:568
QYVKQFLTK-TAD	sp|P0CX23|RL2	sp|P0CX23|RL20A_YEAST	xlink	intra-protein xl	141	136	5	F1905_sailerc	1980.109	496.035	4.000	2.2	1	6	0.95	32.23	0.028	sp|P0CX23|RL20A_YEAST:136:x:sp|P0CX23|RL20A_YEAST:141
QKSVETSNGGVE	sp|Q03862|AR	sp|Q03862|ARX1_YEAST	xlink	intra-protein xl	578	570	8	F1905_sailerc	3264.671	817.176	4.000	2.7	2	5	0.51	32.09	0.028	sp|Q03862|ARX1_YEAST:570:x:sp|Q03862|ARX1_YEAST:578

monolinks

TLTAKALATESGIN	sp|P32794|AF	-	monolink	monolink	568	n/a	-	F1905_sailerc	2103.184	702.069	3.000	0.9	78	29	0.49	58.39	0.000	sp|P32794|AFG2_YEAST:568
AQVQPPYATKVT	sp|P32794|AF	-	monolink	monolink	128	n/a	-	F1905_sailerc	3308.632	1103.885	3.000	0.2	74	35	0.23	55.33	0.000	sp|P32794|AFG2_YEAST:128
TRDLDLIYNDLST	sp|Q08004|BU	-	monolink	monolink	28	n/a	-	F1905_sailerc	2393.234	798.753	3.000	0.8	7	30	0.45	54.90	0.000	sp|Q08004|BUD20_YEAST:28
VLLSAPLSKELR-K	sp|P05743|RL2	-	monolink	monolink	37,37	n/a	-	F1905_sailerc	1480.887	494.637	3.000	0.0	5	23	0.63	52.14	0.000	sp|P05743|RL26A_YEAST:37:|:sp|P53221|RL26B_YEAST:37
SSVGVIINKMVG	sp|Q02753|RL2	-	monolink	monolink	78,78	n/a	-	F1905_sailerc	1628.892	543.972	3.000	0.3	4	25	0.59	51.00	0.000	sp|Q02753|RL21A_YEAST:78:|:sp|Q12672|RL21B_YEAST:78
LVNHFIQEFKR-K	sp|P10591|HS	-	monolink	monolink	243	n/a	-	F1905_sailerc	1585.863	529.629	3.000	1.0	2	22	0.29	50.87	0.000	sp|P10591|HSP71_YEAST:243
GAVSGQKAGLIA	sp|P0CX45|RL2	-	monolink	monolink	234	n/a	-	F1905_sailerc	1453.826	485.617	3.000	0.7	2	28	0.36	50.77	0.000	sp|P0CX45|RL2A_YEAST:234
MEEHMSTLGHD	sp|Q02892|NO	-	monolink	monolink	548	n/a	-	F1905_sailerc	2613.164	872.063	3.000	0.8	8	31	0.00	50.73	0.000	sp|Q02892|NOG1_YEAST:548
VLEQLSGQTPVQ	sp|P0C0W9|RL	-	monolink	monolink	49,49	n/a	-	F1905_sailerc	1895.049	632.691	3.000	0.8	2	24	0.37	50.61	0.000	sp|P0C0W9|RL11A_YEAST:49:|:sp|Q3E757|RL11B_YEAST:49
RGGQSKACPVGN	sp|P40010|NU	-	monolink	monolink	309	n/a	-	F1905_sailerc	2299.174	767.399	3.000	1.6	3	27	0.32	50.28	0.000	sp|P40010|NUG1_YEAST:309
FDILTKQFSR-K6-	sp|P32794|AF	-	monolink	monolink	425	n/a	-	F1905_sailerc	1408.772	470.599	3.000	0.4	2	20	0.00	50.22	0.000	sp|P32794|AFG2_YEAST:425
RAEAKAQGVAVQ	sp|Q02753|RL2	-	monolink	monolink	120,120	n/a	-	F1905_sailerc	1623.932	542.318	3.000	0.7	2	29	0.52	50.22	0.000	sp|Q02753|RL21A_YEAST:120:|:sp|Q12672|RL21B_YEAST:120
TLTAKALATESGIN	sp|P32794|AF	-	monolink	monolink	568	n/a	-	F1905_sailerc	2102.200	701.741	3.000	0.9	28	25	0.57	50.16	0.000	sp|P32794|AFG2_YEAST:568
RAEAKAQGVAVQ	sp|Q02753|RL2	-	monolink	monolink	120,120	n/a	-	F1905_sailerc	1622.947	541.990	3.000	0.3	5	25	0.46	50.06	0.000	sp|Q02753|RL21A_YEAST:120:|:sp|Q12672|RL21B_YEAST:120
FDILTKQFSR-K6-	sp|P32794|AF	-	monolink	monolink	425	n/a	-	F1905_sailerc	1409.756	470.926	3.000	0.2	2	23	0.00	49.80	0.000	sp|P32794|AFG2_YEAST:425
LPAASLGDMVMA	sp|P0CX41|RL2	-	monolink	monolink	63	n/a	-	F1905_sailerc	1786.959	596.661	3.000	1.2	2	28	0.24	49.77	0.000	sp|P0CX41|RL23A_YEAST:63
SVKFVQGLLQNA	sp|P05740|RL1	-	monolink	monolink	89,89	n/a	-	F1905_sailerc	2113.156	705.393	3.000	1.5	8	29	0.55	49.60	0.000	sp|P05740|RL17A_YEAST:89:|:sp|P46990|RL17B_YEAST:89
GAVSGQKAGLIA	sp|P0CX45|RL2	-	monolink	monolink	234	n/a	-	F1905_sailerc	1452.841	485.288	3.000	-0.3	2	26	0.49	48.89	0.000	sp|P0CX45|RL2A_YEAST:234
GSLEEIIAEGQKK-	sp|P02400|RL	-	monolink	monolink	61	n/a	-	F1905_sailerc	1556.830	519.951	3.000	0.1	2	23	0.56	48.79	0.000	sp|P02400|RLA4_YEAST:61
MKEMIQLPLEASE	sp|P32794|AF	-	monolink	monolink	529	n/a	-	F1905_sailerc	2149.085	717.369	3.000	2.2	15	25	0.34	48.78	0.000	sp|P32794|AFG2_YEAST:529
SVKFVQGLLQNA	sp|P05740|RL1	-	monolink	monolink	89,89	n/a	-	F1905_sailerc	2114.140	705.721	3.000	1.3	9	29	0.47	48.40	0.000	sp|P05740|RL17A_YEAST:89:|:sp|P46990|RL17B_YEAST:89
QALILKQFGIAASE	sp|P33201|MR	-	monolink	monolink	204	n/a	-	F1905_sailerc	1919.077	640.700	3.000	-0.1	2	27	0.45	48.27	0.000	sp|P33201|MRT4_YEAST:204
GLDATKLYVSHIQ	sp|P05740|RL1	-	monolink	monolink	111,111	n/a	-	F1905_sailerc	2236.220	746.414	3.000	-0.8	5	27	0.52	48.20	0.000	sp|P05740|RL17A_YEAST:111:|:sp|P46990|RL17B_YEAST:111
LKVPPTIAQFQYTI	sp|P17076|RL8	-	monolink	monolink	70,70	n/a	-	F1905_sailerc	2045.120	682.715	3.000	0.4	1	25	0.50	48.04	0.000	sp|P17076|RL8A_YEAST:70:|:sp|P29453|RL8B_YEAST:70
EITDIKQFLELTR-	sp|P49167|RL3	-	monolink	monolink	9	n/a	-	F1905_sailerc	1759.975	587.666	3.000	1.5	4	24	0.77	47.94	0.000	sp|P49167|RL38_YEAST:9
SKEQLASQLVDLK	sp|P0CX84|RL3	-	monolink	monolink	14	n/a	-	F1905_sailerc	1740.994	581.339	3.000	-2.8	2	24	0.39	47.89	0.000	sp|P0CX84|RL35A_YEAST:14
IFASNEVIAKSR-K	sp|P0CX23|RL2	-	monolink	monolink	38	n/a	-	F1905_sailerc	1489.815	497.613	3.000	0.5	1	19	0.43	47.62	0.000	sp|P0CX23|RL20A_YEAST:38
KSSASSNSADAKA	sp|P32794|AF	-	monolink	monolink	24	n/a	-	F1905_sailerc	1593.784	532.269	3.000	-0.1	2	29	0.31	47.59	0.000	sp|P32794|AFG2_YEAST:24
MKEMIQLPLEASE	sp|P32794|AF	-	monolink	monolink	529	n/a	-	F1905_sailerc	2148.097	717.040	3.000	0.3	23	24	0.39	47.49	0.000	sp|P32794|AFG2_YEAST:529
GSSSLYTLVINDAC	sp|P49167|RL3	-	monolink	monolink	61	n/a	-	F1905_sailerc	1878.996	627.340	3.000	1.0	4	29	0.43	47.43	0.000	sp|P49167|RL38_YEAST:61
MIDFLNAVVPIKH	sp|P38861|NM	-	monolink	monolink	221	n/a	-	F1905_sailerc	1908.091	478.031	4.000	0.2	4	21	0.53	47.28	0.000	sp|P38861|NMD3_YEAST:221
KFNTEESGVDLHE	sp|P32794|AF	-	monolink	monolink	699	n/a	-	F1905_sailerc	2114.031	705.685	3.000	1.4	3	27	0.27	47.23	0.000	sp|P32794|AFG2_YEAST:699
VAAKIAALAGQQ-	sp|P26321|RL5	-	monolink	monolink	289	n/a	-	F1905_sailerc	1295.745	432.923	3.000	0.3	1	24	0.28	47.06	0.000	sp|P26321|RL5_YEAST:289
NILQESVLNKYR-K	sp|Q03862|AR	-	monolink	monolink	27	n/a	-	F1905_sailerc	1631.891	544.971	3.000	1.6	2	22	0.40	46.99	0.000	sp|Q03862|ARX1_YEAST:27
YIASKTHGYVGAD	sp|P32794|AF	-	monolink	monolink	448	n/a	-	F1905_sailerc	2250.151	751.058	3.000	1.9	17	29	0.48	46.95	0.000	sp|P32794|AFG2_YEAST:448
NSLPDSVKIQR-K8	sp|Q12522|IF6	-	monolink	monolink	93	n/a	-	F1905_sailerc	1410.783	471.269	3.000	-0.4	2	19	0.28	46.88	0.000	sp|Q12522|IF6_YEAST:93
KTVKAQRPITGAS	sp|P04449|RL2	-	monolink	monolink	77,77	n/a	-	F1905_sailerc	2094.242	524.568	4.000	0.5	4	28	0.69	46.76	0.000	sp|P04449|RL24A_YEAST:77:|:sp|P24000|RL24B_YEAST:77
IINEPTAAAIAYGL	sp|P10591|HS	-	monolink	monolink	185	n/a	-	F1905_sailerc	1943.061	648.695	3.000	-0.1	2	28	0.44	46.67	0.000	sp|P10591|HSP71_YEAST:185
VTLKDVESAMVD	sp|P32794|AF	-	monolink	monolink	489	n/a	-	F1905_sailerc	2273.180	569.303	4.000	1.8	15	25	0.46	46.50	0.000	sp|P32794|AFG2_YEAST:489
GHRVEKIPEIPLVV	sp|P10664|RL4	-	monolink	monolink	144,144	n/a	-	F1905_sailerc	2742.521	915.181	3.000	1.5	20	23	0.36	46.42	0.000	sp|P10664|RL4A_YEAST:144:|:sp|P49626|RL4B_YEAST:144
SFGKMEEHMSTL	sp|Q02892|NO	-	monolink	monolink	530	n/a	-	F1905_sailerc	2633.192	878.738	3.000	-0.3	4	30	0.28	46.36	0.000	sp|Q02892|NOG1_YEAST:530
HVLDSEAIKYIASK	sp|P32794|AF	-	monolink	monolink	443	n/a	-	F1905_sailerc	1728.929	577.317	3.000	-0.7	4	21	0.58	46.35	0.000	sp|P32794|AFG2_YEAST:443
YIASKTHGYVGAD	sp|P32794|AF	-	monolink	monolink	448	n/a	-	F1905_sailerc	2251.135	751.386	3.000	1.7	26	30	0.47	46.35	0.000	sp|P32794|AFG2_YEAST:448
KANLLLVNKADLL	sp|P53145|LS	-	monolink	monolink	237	n/a	-	F1905_sailerc	1809.099	604.041	3.000	0.6	4	24	0.50	46.17	0.000	sp|P53145|LSG1_YEAST:237
TKDSNKDVVVEGI	sp|P41805|RL1	-	monolink	monolink	141	n/a	-	F1905_sailerc	1714.911	572.645	3.000	0.5	2	24	0.59	46.00	0.000	sp|P41805|RL10_YEAST:141
SKEQLASQLVDLK	sp|P0CX84|RL3	-	monolink	monolink	14	n/a	-	F1905_sailerc	1741.983	581.669	3.000	0.2	4	23	0.53	45.95	0.000	sp|P0CX84|RL35A_YEAST:14
SALGETQKDTYQ	sp|P53742|NO	-	monolink	monolink	101	n/a	-	F1905_sailerc	1977.042	660.022	3.000	-0.2	3	24	0.56	45.53	0.000	sp|P53742|NOG2_YEAST:101
DKNILQESVLNKY	sp|Q03862|AR	-	monolink	monolink	27	n/a	-	F1905_sailerc	1875.012	626.012	3.000	0.8	3	26	0.47	45.47	0.000	sp|Q03862|ARX1_YEAST:27
KVLIDGPKAGVPR	sp|P36105|RL1	-	monolink	monolink	50,50	n/a	-	F1905_sailerc	1504.900	502.641	3.000	1.2	5	21	0.46	45.42	0.000	sp|P36105|RL14A_YEAST:50:|:sp|P38754|RL14B_YEAST:50
KYNLPEPLSYAAV	sp|P32794|AF	-	monolink	monolink	252	n/a	-	F1905_sailerc	2789.480	930.834	3.000	2.2	18	25	0.54	45.41	0.000	sp|P32794|AFG2_YEAST:252
GLGTDANIDKFSL	sp|P32794|AF	-	monolink	monolink	481	n/a	-	F1905_sailerc	1633.858	545.627	3.000	0.8	4	26	0.50	45.38	0.000	sp|P32794|AFG2_YEAST:481
KASGEIVSINQINE	sp|P0CX23|RL2	-	monolink	monolink	53	n/a	-	F1905_sailerc	2190.165	731.063	3.000	0.1	2	25	0.36	45.27	0.000	sp|P0CX23|RL20A_YEAST:53
KFLDGIYVSHK-K1	sp|P05738|RL9	-	monolink	monolink	174,174	n/a	-	F1905_sailerc	1460.804	487.943	3.000	1.2	2	19	0.55	45.21	0.000	sp|P05738|RL9A_YEAST:174:|:sp|P51401|RL9B_YEAST:174
AQVQPPYATKVT	sp|P32794|AF	-	monolink	monolink	128	n/a	-	F1905_sailerc	3307.655	1103.559	3.000	2.3	22	27	0.25	45.08	0.000	sp|P32794|AFG2_YEAST:128
NVKHSGNIQLDEI	sp|P0CX53|RL1	-	monolink	monolink	99	n/a	-	F1905_sailerc	2204.184	735.736	3.000	1.8	6	28	0.48	44.87	0.000	sp|P0CX53|RL12A_YEAST:99
EITDIKQFLELTR-K	sp|P49167|RL3	-	monolink	monolink	9	n/a	-	F1905_sailerc	1760.957	587.993	3.000	0.3	3	21	0.57	44.87	0.000	sp|P49167|RL38_YEAST:9
GRIPNVPVIVKAR-	sp|P02406|RL2	-	monolink	monolink	126	n/a	-	F1905_sailerc	1572.982	525.335	3.000	-0.4	2	21	0.47	44.78	0.000	sp|P02406|RL28_YEAST:126
YNLPEPLSYAAVG	sp|P32794|AF	-	monolink	monolink	252	n/a	-	F1905_sailerc	2661.384	888.136	3.000	2.0	13	25	0.43	44.72	0.000	sp|P32794|AFG2_YEAST:252
KAQVQPPYATKV	sp|P32794|AF	-	monolink	monolink	128	n/a	-	F1905_sailerc	3436.732	860.191	4.000	1.9	26	27	0.39	44.71	0.000	sp|P32794|AFG2_YEAST:128
TGTKPAAVFTETL	sp|P10664|RL4	-	monolink	monolink	350	n/a	-	F1905_sailerc	1869.985	624.336	3.000	1.0	4	20	0.41	44.61	0.000	sp|P10664|RL4A_YEAST:350
QAYAVSEKAGHQ	sp|P10664|RL4	-	monolink	monolink	55,55	n/a	-	F1905_sailerc	2475.175	826.066	3.000	-1.0	2	30	0.43	44.53	0.000	sp|P10664|RL4A_YEAST:55:|:sp|P49626|RL4B_YEAST:55
KGQEGKISSVYR-K	sp|P05743|RL2	-	monolink	monolink	64,64	n/a	-	F1905_sailerc	1505.820	502.948	3.000	-0.0	2	22	0.35	44.42	0.000	sp|P05743|RL26A_YEAST:64:|:sp|P53221|RL26B_YEAST:64
IVKAGGECITLDQ	sp|P0CX49|RL1	-	monolink	monolink	116	n/a	-	F1905_sailerc	1998.083	667.035	3.000	0.5	5	28	0.54	44.33	0.000	sp|P0CX49|RL18A_YEAST:116
ALVEHIIQAKADA	sp|P0CX82|RL1	-	monolink	monolink	146	n/a	-	F1905_sailerc	1817.019	606.681	3.000	1.6	2	23	0.46	44.33	0.000	sp|P0CX82|RL19A_YEAST:146
NHYKISLAAISR-K	sp|Q02892|NO	-	monolink	monolink	92	n/a	-	F1905_sailerc	1527.840	510.288	3.000	-0.8	2	22	0.52	44.29	0.000	sp|Q02892|NOG1_YEAST:92
GSTHITFSKETQA	sp|P32794|AF	-	monolink	monolink	228	n/a	-	F1905_sailerc	1830.922	611.315	3.000	-0.3	4	21	0.61	44.27	0.000	sp|P32794|AFG2_YEAST:228
IINSSEIQSAIRPAC	sp|P10664|RL4	-	monolink	monolink	308,308	n/a	-	F1905_sailerc	2422.325	808.450	3.000	-1.6	7	30	0.40	44.26	0.000	sp|P10664|RL4A_YEAST:308:|:sp|P49626|RL4B_YEAST:308
TKEAVAALKAVG	sp|P10664|RL4	-	monolink	monolink	170,170	n/a	-	F1905_sailerc	2077.177	520.302	4.000	-0.4	9	25	0.60	44.21	0.000	sp|P10664|RL4A_YEAST:170:|:sp|P49626|RL4B_YEAST:170
KVLIDGPKAGVPR	sp|P36105|RL1	-	monolink	monolink	50,50	n/a	-	F1905_sailerc	1503.915	502.313	3.000	0.9	3	19	0.59	44.14	0.000	sp|P36105|RL14A_YEAST:50:|:sp|P38754|RL14B_YEAST:50
KASGEIVSINQINE	sp|P0CX23|RL2	-	monolink	monolink	53	n/a	-	F1905_sailerc	2191.148	731.390	3.000	-0.3	4	27	0.38	44.13	0.000	sp|P0CX23|RL20A_YEAST:53
MEEHMSTLGHD	sp|Q02892|NO	-	monolink	monolink	548	n/a	-	F1905_sailerc	2612.180	871.734	3.000	0.5	3	30	0.13	44.11	0.000	sp|Q02892|NOG1_YEAST:548
RGGQSKACPVGN	sp|P40010|NU	-	monolink	monolink	309	n/a	-	F1905_sailerc	2300.157	767.727	3.000	1.4	3	27	0.35	44.09	0.000	sp|P40010|NUG1_YEAST:309
VNNQLIKVAVHN	sp|P05738|RL9	-	monolink	monolink	54	n/a	-	F1905_sailerc	1773.986	592.336	3.000	0.5	5	23	0.58	44.04	0.000	sp|P05738|RL9A_YEAST:54
CKGTVGNSHKTT	sp|P0C0W9|RL	-	monolink	monolink	153,153	n/a	-	F1905_sailerc	1572.793	525.272	3.000	-0.2	2	23	0.51	43.97	0.000	sp|P0C0W9|RL11A_YEAST:153:|:sp|Q3E757|RL11B_YEAST:153
MSSDRHVLDSEAI	sp|P32794|AF	-	monolink	monolink	443	n/a	-	F1905_sailerc	2304.177	577.052	4.000	-0.7	4	19	0.58	43.96	0.000	sp|P32794|AFG2_YEAST:443
TKDSNKDVVVEGI	sp|P41805|RL1	-	monolink	monolink	145	n/a	-	F1905_sailerc	1714.912	572.645	3.000	1.4	5	22	0.54	43.95	0.000	sp|P41805|RL10_YEAST:145
VLEQLSGQTPVQ	sp|P0C0W9|RL	-	monolink	monolink	49,49	n/a	-	F1905_sailerc	1896.032	633.018	3.000	0.2	2	22	0.41	43.90	0.000	sp|P0C0W9|RL11A_YEAST:49:|:sp|Q3E757|RL11B_YEAST:49
VLLSAPLSKELR-K	sp|P05743|RL2	-	monolink	monolink	37,37	n/a	-	F1905_sailerc	1479.903	494.309	3.000	0.5	4	22	0.62	43.90	0.000	sp|P05743|RL26A_YEAST:37:|:sp|P53221|RL26B_YEAST:37
MIDFLNAVVPIKH	sp|P38861|NM	-	monolink	monolink	223	n/a	-	F1905_sailerc	1908.090	478.030	4.000	-0.4	2	19	0.48	43.76	0.000	sp|P38861|NMD3_YEAST:223
GSTHITFSKETQA	sp|P32794|AF	-	monolink	monolink	228	n/a	-	F1905_sailerc	1831.908	611.644	3.000	0.9	7	22	0.67	43.76	0.000	sp|P32794|AFG2_YEAST:228
IINSSEIQSAIRPAC	sp|P10664|RL4	-	monolink	monolink	308,308	n/a	-	F1905_sailerc	2423.311	606.836	4.000	-0.8	9	26	0.53	43.76	0.000	sp|P10664|RL4A_YEAST:308:|:sp|P49626|RL4B_YEAST:308
LLGPQLSKAGKFP	sp|P0CX43|RL1	-	monolink	monolink	130	n/a	-	F1905_sailerc	2837.505	946.843	3.000	2.9	3	26	0.36	43.76	0.000	sp|P0CX43|RL1A_YEAST:130
VDKPLLKAGR-K7	sp|P0CX45|RL2	-	monolink	monolink	181	n/a	-	F1905_sailerc	1251.755	418.260	3.000	0.4	2	14	0.00	43.73	0.000	sp|P0CX45|RL2A_YEAST:181
GILLHGPPGTGKT	sp|P32794|AF	-	monolink	monolink	292	n/a	-	F1905_sailerc	1915.110	639.378	3.000	1.3	9	28	0.70	43.73	0.000	sp|P32794|AFG2_YEAST:292
TLASVTKEILGTAC	sp|P0CX53|RL1	-	monolink	monolink	130	n/a	-	F1905_sailerc	2145.150	716.058	3.000	1.8	3	26	0.55	43.68	0.000	sp|P0CX53|RL12A_YEAST:130
KLRDPLAYLEQVR	sp|Q02892|NO	-	monolink	monolink	142	n/a	-	F1905_sailerc	1755.007	586.010	3.000	1.7	4	24	0.59	43.65	0.000	sp|Q02892|NOG1_YEAST:142
LLDDSGVIMPGM	sp|P32794|AF	-	monolink	monolink	168	n/a	-	F1905_sailerc	2374.267	792.430	3.000	1.3	11	24	0.44	43.61	0.000	sp|P32794|AFG2_YEAST:168
KVACIGAWHPAH	sp|P14126|RL3	-	monolink	monolink	248	n/a	-	F1905_sailerc	2330.194	583.556	4.000	0.5	5	25	0.58	43.58	0.000	sp|P14126|RL3_YEAST:248
WAAAAVCEKWA	sp|P36105|RL1	-	monolink	monolink	93	n/a	-	F1905_sailerc	2047.989	683.671	3.000	3.0	2	24	0.37	43.53	0.000	sp|P36105|RL14A_YEAST:93
IPPKPRKVLQLLR-	sp|P05737|RL7	-	monolink	monolink	101,101	n/a	-	F1905_sailerc	1713.103	429.284	4.000	-0.0	2	19	0.92	43.43	0.000	sp|P05737|RL7A_YEAST:101:|:sp|Q12213|RL7B_YEAST:101
VTVGSLQGYNILE	sp|P32794|AF	-	monolink	monolink	146	n/a	-	F1905_sailerc	2693.364	674.349	4.000	-0.6	16	26	0.53	43.37	0.000	sp|P32794|AFG2_YEAST:146
GHRVEKIPEIPLVV	sp|P10664|RL4	-	monolink	monolink	144,144	n/a	-	F1905_sailerc	2741.537	686.392	4.000	1.7	7	20	0.53	43.30	0.000	sp|P10664|RL4A_YEAST:144:|:sp|P49626|RL4B_YEAST:144
TGTKPAAVFTETL	sp|P10664|RL4	-	monolink	monolink	360	n/a	-	F1905_sailerc	1870.969	624.664	3.000	0.8	2	22	0.44	43.27	0.000	sp|P10664|RL4A_YEAST:360
GFTLAEVKAAGLT	sp|Q12690|RL	-	monolink	monolink	81,81	n/a	-	F1905_sailerc	1965.058	656.027	3.000	0.5	4	27	0.54	43.25	0.000	sp|P40212|RL13B_YEAST:81:|:sp|Q12690|RL13A_YEAST:81
DLDLIYNDLSTKES	sp|Q08004|BU	-	monolink	monolink	28	n/a	-	F1905_sailerc	2136.085	713.036	3.000	0.7	7	26	0.52	43.18	0.000	sp|Q08004|BUD20_YEAST:28
VTLKDVESAMVD	sp|P32794|AF	-	monolink	monolink	489	n/a	-	F1905_sailerc	2272.192	569.056	4.000	0.2	10	21	0.46	43.16	0.000	sp|P32794|AFG2_YEAST:489
VYWSDIGGQEEL	sp|P32794|AF	-	monolink	monolink	525	n/a	-	F1905_sailerc	1906.969	636.664	3.000	0.9	2	20	0.50	43.13	0.000	sp|P32794|AFG2_YEAST:525
KKVMPAIVVR-K2	sp|P0CX41|RL2	-	monolink	monolink	72	n/a	-	F1905_sailerc	1295.801	432.941	3.000	0.6	4	18	0.00	43.08	0.000	sp|P0CX41|RL23A_YEAST:72
AKNPLTHSTPKNF	sp|P29453|RL8	-	monolink	monolink	23	n/a	-	F1905_sailerc	2488.345	623.094	4.000	0.6	1	20	0.64	42.91	0.000	sp|P29453|RL8B_YEAST:23
LLDDSGVIMPGM	sp|P32794|AF	-	monolink	monolink	168	n/a	-	F1905_sailerc	2375.256	792.760	3.000	3.5	11	22	0.39	42.91	0.000	sp|P32794|AFG2_YEAST:168
TKEAVAALKAVG	sp|P10664|RL4	-	monolink	monolink	170,170	n/a	-	F1905_sailerc	2076.195	520.057	4.000	0.5	7	23	0.58	42.79	0.000	sp|P10664|RL4A_YEAST:170:|:sp|P49626|RL4B_YEAST:170
IKVVKTPGGILR-K	sp|P87262|RL3	-	monolink	monolink	24,24	n/a	-	F1905_sailerc	1435.911	479.645	3.000	-1.1	2	18	0.30	42.77	0.000	sp|P40525|RL34B_YEAST:24:|:sp|P87262|RL34A_YEAST:24
FKLPAEFITRPHPS	sp|P32794|AF	-	monolink	monolink	29	n/a	-	F1905_sailerc	1922.078	481.527	4.000	-0.2	3	16	0.71	42.74	0.000	sp|P32794|AFG2_YEAST:29
NKFEIPTKIK-K8-1	sp|P33201|MR	-	monolink	monolink	174	n/a	-	F1905_sailerc	1372.797	458.607	3.000	0.4	2	16	0.53	42.73	0.000	sp|P33201|MRT4_YEAST:174
KLRDPLAYLEQVR	sp|Q02892|NO	-	monolink	monolink	142	n/a	-	F1905_sailerc	1755.990	586.338	3.000	0.8	3	22	0.58	42.66	0.000	sp|Q02892|NOG1_YEAST:142
TTKEDTVSWFKQ	sp|P0C0W9|RL	-	monolink	monolink	164,164	n/a	-	F1905_sailerc	1752.896	585.307	3.000	1.5	1	19	0.36	42.64	0.000	sp|P0C0W9|RL11A_YEAST:164:|:sp|Q3E757|RL11B_YEAST:164
GATYGKPTNQGV	sp|P05748|RL1	-	monolink	monolink	93,93	n/a	-	F1905_sailerc	2278.165	760.396	3.000	-2.3	3	28	0.42	42.63	0.000	sp|P05748|RL15A_YEAST:93:|:sp|P54780|RL15B_YEAST:93

ALVEHIIQAKADA	sp|P0CX82|RL1-	monolink	monolink	146	n/a	-	F1905_sailerc	1818.003	607.009	3.000	1.4	4	23	0.65	42.59	0.000	sp|P0CX82|RL19A_YEAST:146
SVALDLYFQNKKN	sp|P47019|ALE-	monolink	monolink	62	n/a	-	F1905_sailerc	4026.024	1343.016	3.000	1.6	7	34	0.39	42.58	0.000	sp|P47019|ALB1_YEAST:62
NVKHSGNIQLDEI	sp|P0CX53|RL1-	monolink	monolink	99	n/a	-	F1905_sailerc	2203.202	735.408	3.000	2.5	1	23	0.64	42.55	0.000	sp|P0CX53|RL12A_YEAST:99
GVVWTESHQEA	sp|Q03862|AR-	monolink	monolink	285	n/a	-	F1905_sailerc	3153.550	1052.191	3.000	1.1	11	26	0.31	42.48	0.000	sp|Q03862|ARX1_YEAST:285
LNPYAKVFAAEK	sp|P10664|RL4-	monolink	monolink	332,332	n/a	-	F1905_sailerc	1505.813	502.946	3.000	0.3	2	21	0.62	42.33	0.000	sp|P10664|RL4A_YEAST:332:|:sp|P49626|RL4B_YEAST:332
IFEFPKTTVAALR	sp|P0CX49|RL1-	monolink	monolink	98	n/a	-	F1905_sailerc	1646.941	549.988	3.000	0.8	3	24	0.51	42.30	0.000	sp|P0CX49|RL18A_YEAST:98
SFGKMEEHMSTL	sp|Q02892|NC-	monolink	monolink	530	n/a	-	F1905_sailerc	2634.179	879.067	3.000	1.0	12	26	0.25	42.29	0.000	sp|Q02892|NOG1_YEAST:530
HALHGTAKALAA	sp|P05747|RL2-	monolink	monolink	52	n/a	-	F1905_sailerc	1513.873	505.632	3.000	0.4	5	29	0.72	42.24	0.000	sp|P05747|RL29_YEAST:52
LCSGVTGLLFTDE	sp|P33201|MR-	monolink	monolink	113	n/a	-	F1905_sailerc	2790.378	931.134	3.000	4.0	7	26	0.26	42.21	0.000	sp|P33201|MRT4_YEAST:113
EIFLEMPKVYWSD	sp|P32794|AF-	monolink	monolink	512	n/a	-	F1905_sailerc	2666.319	889.781	3.000	-0.1	11	28	0.33	42.18	0.000	sp|P32794|AFG2_YEAST:512
GLDATKLYVSHIQ	sp|P05740|RL1-	monolink	monolink	111,111	n/a	-	F1905_sailerc	2237.204	746.742	3.000	-0.7	4	26	0.65	42.09	0.000	sp|P05740|RL17A_YEAST:111:|:sp|P46990|RL17B_YEAST:111
KVVKEQTEAAK-K	sp|P87262|RL3-	monolink	monolink	103,103	n/a	-	F1905_sailerc	1384.793	462.605	3.000	-0.1	2	25	0.59	41.97	0.000	sp|P40525|RL34B_YEAST:103:|:sp|P87262|RL34A_YEAST:103
AEKTDLTNILIR-K	sp|P39715|EC-	monolink	monolink	57	n/a	-	F1905_sailerc	1541.866	514.963	3.000	-0.2	3	22	0.63	41.89	0.000	sp|P39715|ECM1_YEAST:57
HVLDSEAIKYIASK	sp|P32794|AF-	monolink	monolink	443	n/a	-	F1905_sailerc	1727.948	576.991	3.000	1.5	3	18	0.77	41.86	0.000	sp|P32794|AFG2_YEAST:443
ELVATTLKAMAR-	sp|Q07915|RL-	monolink	monolink	91	n/a	-	F1905_sailerc	1458.813	487.279	3.000	0.7	3	21	0.38	41.78	0.000	sp|Q07915|RLP24_YEAST:91
RGVFQKAVDAR-	sp|P38202|YB-	monolink	monolink	24	n/a	-	F1905_sailerc	1401.772	468.265	3.000	-0.5	2	19	0.25	41.73	0.000	sp|P38202|YBC8_YEAST:24
SKEQLASQLVDLK	sp|P0CX84|RL3-	monolink	monolink	25	n/a	-	F1905_sailerc	1741.984	581.669	3.000	1.0	1	25	0.47	41.71	0.000	sp|P0CX84|RL35A_YEAST:25
AERVEDQKVVDK	sp|Q02326|RL-	monolink	monolink	139	n/a	-	F1905_sailerc	1570.822	524.615	3.000	1.3	2	20	0.53	41.60	0.000	sp|Q02326|RL6A_YEAST:139
SCGVDAMSVDDL	sp|P0CX43|RL1-	monolink	monolink	91	n/a	-	F1905_sailerc	1679.775	560.933	3.000	0.1	3	22	0.00	41.58	0.000	sp|P0CX43|RL1A_YEAST:91
RGPLVVYAEDNGI	sp|P10664|RL4-	monolink	monolink	217,217	n/a	-	F1905_sailerc	2125.191	709.405	3.000	0.7	2	26	0.57	41.52	0.000	sp|P10664|RL4A_YEAST:217:|:sp|P49626|RL4B_YEAST:217
IFEFPKTTVAALR	sp|P0CX49|RL1-	monolink	monolink	98	n/a	-	F1905_sailerc	1647.925	550.316	3.000	0.9	2	24	0.67	41.49	0.000	sp|P0CX49|RL18A_YEAST:98
KVVIVKPHDEGSK	sp|P0C2H6|RL-	monolink	monolink	27,27	n/a	-	F1905_sailerc	1590.899	531.307	3.000	0.2	7	22	0.65	41.46	0.000	sp|P0C2H6|RL27A_YEAST:27:|:sp|P0C2H7|RL27B_YEAST:27
LATRPKCGDCGS	sp|P87262|RL3-	monolink	monolink	43,43	n/a	-	F1905_sailerc	2668.392	668.106	4.000	0.7	5	20	0.53	41.40	0.000	sp|P40525|RL34B_YEAST:43:|:sp|P87262|RL34A_YEAST:43
EIESLKSAIEIPLHQ	sp|P32794|AF-	monolink	monolink	258	n/a	-	F1905_sailerc	3233.734	809.441	4.000	0.2	6	32	0.54	41.40	0.000	sp|P32794|AFG2_YEAST:258
LAPELNQAIWKR-	sp|P0C2H8|RL-	monolink	monolink	61,61	n/a	-	F1905_sailerc	1592.906	531.976	3.000	1.0	4	21	0.61	41.36	0.000	sp|P0C2H8|RL31A_YEAST:61:|:sp|P0C2H9|RL31B_YEAST:61
LGTLVNQKTSAV	sp|P17076|RL8-	monolink	monolink	193,193	n/a	-	F1905_sailerc	2126.197	709.740	3.000	1.3	1	23	0.59	41.29	0.000	sp|P17076|RL8A_YEAST:193:|:sp|P29453|RL8B_YEAST:193
LFHSIKPLFANK-K	sp|Q02892|NC-	monolink	monolink	275	n/a	-	F1905_sailerc	1568.908	523.977	3.000	-0.1	4	20	0.55	41.17	0.000	sp|Q02892|NOG1_YEAST:275
YAKIHFESEAK-K3	sp|Q06338|BC-	monolink	monolink	246	n/a	-	F1905_sailerc	1476.761	493.261	3.000	-0.3	2	17	0.49	40.99	0.000	sp|Q06338|BCP1_YEAST:246
TVKSLVDNMITG	sp|P05738|RL-	monolink	monolink	72,72	n/a	-	F1905_sailerc	1759.978	587.667	3.000	1.4	1	24	0.48	40.96	0.000	sp|P05738|RL9A_YEAST:72:|:sp|P51401|RL9B_YEAST:72
GIVKQIVHDSGR-	sp|P0CX45|RL-	monolink	monolink	46	n/a	-	F1905_sailerc	1462.827	488.617	3.000	0.9	2	24	0.50	40.96	0.000	sp|P0CX45|RL2A_YEAST:46
ETQPMKQKSVET	sp|Q03862|AR-	monolink	monolink	578	n/a	-	F1905_sailerc	2593.225	865.416	3.000	0.1	7	25	0.34	40.94	0.000	sp|Q03862|ARX1_YEAST:578
ALKQEGAANKTV	sp|P0CX49|RL1-	monolink	monolink	79	n/a	-	F1905_sailerc	2496.358	833.127	3.000	1.3	1	24	0.58	40.91	0.000	sp|P0CX49|RL18A_YEAST:79
KKVMPAIVVR-K1	sp|P0CX41|RL-	monolink	monolink	71	n/a	-	F1905_sailerc	1295.801	432.942	3.000	0.9	1	14	0.31	40.88	0.000	sp|P0CX41|RL23A_YEAST:71
AVGAHSDLLKVLK	sp|P10664|RL4-	monolink	monolink	180,180	n/a	-	F1905_sailerc	1504.900	502.641	3.000	1.2	2	20	0.50	40.86	0.000	sp|P10664|RL4A_YEAST:180:|:sp|P49626|RL4B_YEAST:180
TKDSNKDVVVEGI	sp|P41805|RL1-	monolink	monolink	145	n/a	-	F1905_sailerc	1713.928	572.317	3.000	1.4	1	23	0.65	40.84	0.000	sp|P41805|RL10_YEAST:145
AQKYLDQVLDHQ	sp|P46990|RL1-	monolink	monolink	46	n/a	-	F1905_sailerc	1768.911	590.645	3.000	0.1	3	22	0.55	40.83	0.000	sp|P46990|RL17B_YEAST:46
AKIVKAGGECITL	sp|P0CX49|RL1-	monolink	monolink	116	n/a	-	F1905_sailerc	2197.215	733.413	3.000	0.5	3	26	0.49	40.81	0.000	sp|P0CX49|RL18A_YEAST:116
VTVGSLQGYNILE	sp|P32794|AF-	monolink	monolink	146	n/a	-	F1905_sailerc	2692.384	674.104	4.000	0.9	5	22	0.54	40.80	0.000	sp|P32794|AFG2_YEAST:146
GATYGKPTNQGV	sp|P05748|RL1-	monolink	monolink	93,93	n/a	-	F1905_sailerc	2279.164	760.729	3.000	4.0	3	27	0.41	40.73	0.000	sp|P05748|RL15A_YEAST:93:|:sp|P54780|RL15B_YEAST:93
GQSAGKLAAIVEII	sp|P36105|RL1-	monolink	monolink	31,31	n/a	-	F1905_sailerc	1896.057	633.027	3.000	0.3	1	23	0.71	40.72	0.000	sp|P36105|RL14A_YEAST:31:|:sp|P38754|RL14B_YEAST:31
SKLVTLAQTDKK-	sp|P33201|MR-	monolink	monolink	8	n/a	-	F1905_sailerc	1485.878	496.300	3.000	0.8	4	18	0.59	40.72	0.000	sp|P33201|MRT4_YEAST:8
HALHGTAKALAA	sp|P05747|RL2-	monolink	monolink	52	n/a	-	F1905_sailerc	1514.857	505.960	3.000	-0.2	5	24	0.69	40.70	0.000	sp|P05747|RL29_YEAST:52
FKLPAEFITRPHPS	sp|P32794|AF-	monolink	monolink	29	n/a	-	F1905_sailerc	1923.062	481.773	4.000	0.1	4	20	0.45	40.65	0.000	sp|P32794|AFG2_YEAST:29
VVYLKHLEDNTLL	sp|P05739|RL6-	monolink	monolink	56	n/a	-	F1905_sailerc	2340.310	781.111	3.000	0.4	2	23	0.92	40.65	0.000	sp|P05739|RL6B_YEAST:56
ASGNYVIIIGHNP	sp|P0CX45|RL-	monolink	monolink	145	n/a	-	F1905_sailerc	2253.143	752.055	3.000	1.7	3	22	0.44	40.60	0.000	sp|P0CX45|RL2A_YEAST:145
LSRPSLPKIK-K8-1	sp|P0CX84|RL3-	monolink	monolink	43	n/a	-	F1905_sailerc	1293.803	432.275	3.000	0.4	3	18	0.48	40.56	0.000	sp|P0CX84|RL35A_YEAST:43
GFTLAEVKAAGLT	sp|Q12690|RL-	monolink	monolink	81,81	n/a	-	F1905_sailerc	1964.074	655.699	3.000	0.5	2	26	0.56	40.48	0.000	sp|P40212|RL13B_YEAST:81:|:sp|Q12690|RL13A_YEAST:81
KAQVQPPYATKV	sp|P32794|AF-	monolink	monolink	128	n/a	-	F1905_sailerc	3435.741	859.943	4.000	-0.1	12	25	0.35	40.43	0.000	sp|P32794|AFG2_YEAST:128
VVANTSNAHVLTI	sp|P32794|AF-	monolink	monolink	318	n/a	-	F1905_sailerc	3379.809	845.960	4.000	3.1	57	24	0.55	40.37	0.000	sp|P32794|AFG2_YEAST:318
AKNPLTHSTPK-K2	sp|P29453|RL8-	monolink	monolink	23	n/a	-	F1905_sailerc	1347.751	450.258	3.000	0.1	2	16	0.35	40.37	0.000	sp|P29453|RL8B_YEAST:23
ALATESGINFLAV	sp|P32794|AF-	monolink	monolink	582	n/a	-	F1905_sailerc	2374.281	792.435	3.000	0.9	8	32	0.44	40.30	0.000	sp|P32794|AFG2_YEAST:582
VFGALKGASDGGI	sp|P26321|RL5-	monolink	monolink	164	n/a	-	F1905_sailerc	2328.187	777.070	3.000	0.2	6	39	0.46	40.29	0.000	sp|P26321|RL5_YEAST:164
KNESQNSTAVTLC	sp|P47019|ALE-	monolink	monolink	63	n/a	-	F1905_sailerc	2747.363	916.796	3.000	2.1	15	25	0.28	40.13	0.000	sp|P47019|ALB1_YEAST:63
KVWLDPNETSEIA	sp|P0CX82|RL1-	monolink	monolink	21	n/a	-	F1905_sailerc	2212.113	738.379	3.000	0.2	3	20	0.48	40.01	0.000	sp|P0CX82|RL19A_YEAST:21
TYAAEIAHNISAK	sp|P38061|RL3-	monolink	monolink	103	n/a	-	F1905_sailerc	1813.931	605.652	3.000	-0.4	4	21	0.43	40.01	0.000	sp|P38061|RL32_YEAST:103
RVLLSAPLSKELR	sp|P05743|RL2-	monolink	monolink	37,37	n/a	-	F1905_sailerc	1636.988	546.671	3.000	0.4	2	19	0.71	39.97	0.000	sp|P05743|RL26A_YEAST:37:|:sp|P53221|RL26B_YEAST:37
KAHLAEIQLNGGS	sp|P14126|RL3-	monolink	monolink	175	n/a	-	F1905_sailerc	1949.060	650.694	3.000	0.7	2	23	0.45	39.94	0.000	sp|P14126|RL3_YEAST:175
TLLICGYPNVGKS	sp|Q02892|NC-	monolink	monolink	180	n/a	-	F1905_sailerc	2079.123	694.049	3.000	2.0	2	21	0.50	39.91	0.000	sp|Q02892|NOG1_YEAST:180
YGLNHVVSLIENK	sp|P29453|RL8-	monolink	monolink	147	n/a	-	F1905_sailerc	1767.990	590.338	3.000	0.8	1	21	0.38	39.90	0.000	sp|P29453|RL8B_YEAST:147
GSTHITFSKETQA	sp|P32794|AF-	monolink	monolink	228	n/a	-	F1905_sailerc	1960.004	654.342	3.000	1.2	11	21	0.54	39.87	0.000	sp|P32794|AFG2_YEAST:228
LGTLVNQKTSAV	sp|P17076|RL8-	monolink	monolink	193,193	n/a	-	F1905_sailerc	2125.214	709.413	3.000	1.6	2	20	0.64	39.85	0.000	sp|P17076|RL8A_YEAST:193:|:sp|P29453|RL8B_YEAST:193
KFNTEESGVDLHE	sp|P32794|AF-	monolink	monolink	699	n/a	-	F1905_sailerc	2115.014	706.012	3.000	0.8	4	26	0.36	39.79	0.000	sp|P32794|AFG2_YEAST:699
ASKVLEQLSGQTP	sp|P0C0W9|RL-	monolink	monolink	35,35	n/a	-	F1905_sailerc	1954.075	652.366	3.000	0.9	2	21	0.48	39.76	0.000	sp|P0C0W9|RL11A_YEAST:35:|:sp|Q3E757|RL11B_YEAST:35
GLGTDANIDKFSL	sp|P32794|AF-	monolink	monolink	481	n/a	-	F1905_sailerc	1632.875	545.299	3.000	1.7	5	21	0.56	39.70	0.000	sp|P32794|AFG2_YEAST:481
KVVIVKPHDEGSK	sp|P0C2H6|RL-	monolink	monolink	27,27	n/a	-	F1905_sailerc	1589.914	530.979	3.000	-0.1	3	21	0.61	39.68	0.000	sp|P0C2H6|RL27A_YEAST:27:|:sp|P0C2H7|RL27B_YEAST:27
AKSCGVDAMSVD	sp|P0CX43|RL1-	monolink	monolink	78	n/a	-	F1905_sailerc	1878.907	627.310	3.000	0.0	5	26	0.39	39.62	0.000	sp|P0CX43|RL1A_YEAST:78
LPAASLGDMVMA	sp|P0CX41|RL-	monolink	monolink	64	n/a	-	F1905_sailerc	1785.976	596.333	3.000	1.5	1	20	0.34	39.61	0.000	sp|P0CX41|RL23A_YEAST:64
FLSPSGHKTFLVA	sp|P38061|RL3-	monolink	monolink	72	n/a	-	F1905_sailerc	1900.049	634.357	3.000	1.5	2	25	0.82	39.59	0.000	sp|P38061|RL32_YEAST:72
KVSSASAAASESD	sp|P26785|RL1-	monolink	monolink	176	n/a	-	F1905_sailerc	1661.849	554.957	3.000	0.9	2	19	0.46	39.57	0.000	sp|P26785|RL16B_YEAST:176
MSSDRHVLDSEAI	sp|P32794|AF-	monolink	monolink	443	n/a	-	F1905_sailerc	2305.164	769.396	3.000	0.8	4	24	0.40	39.55	0.000	sp|P32794|AFG2_YEAST:443
ILLDAPCSGTGVIG	sp|P40991|NO-	monolink	monolink	432	n/a	-	F1905_sailerc	2213.171	738.731	3.000	4.1	2	24	0.41	39.36	0.000	sp|P40991|NOP2_YEAST:432
SKLVTLAQTDKK-	sp|P33201|MR-	monolink	monolink	8	n/a	-	F1905_sailerc	1486.861	496.628	3.000	0.1	4	18	0.60	39.35	0.000	sp|P33201|MRT4_YEAST:8
LFNKYRPETAAEK	sp|P17076|RL8-	monolink	monolink	96,96	n/a	-	F1905_sailerc	1849.994	463.506	4.000	0.2	4	19	0.70	39.35	0.000	sp|P17076|RL8A_YEAST:96:|:sp|P29453|RL8B_YEAST:96
IVKAGGECITLDQ	sp|P0CX49|RL1-	monolink	monolink	116	n/a	-	F1905_sailerc	1997.100	666.708	3.000	0.8	2	22	0.59	39.30	0.000	sp|P0CX49|RL18A_YEAST:116
ASKVLEQLSGQTP	sp|P0C0W9|RL-	monolink	monolink	35,35	n/a	-	F1905_sailerc	1955.059	652.694	3.000	0.7	4	19	0.47	39.29	0.000	sp|P0C0W9|RL11A_YEAST:35:|:sp|Q3E757|RL11B_YEAST:35
VEKIPEIPLVVSTD	sp|P10664|RL4-	monolink	monolink	144,144	n/a	-	F1905_sailerc	2392.341	798.455	3.000	2.3	6	22	0.46	39.26	0.000	sp|P10664|RL4A_YEAST:144:|:sp|P49626|RL4B_YEAST:144
YHPGYFGKVGMR	sp|P02406|RL2-	monolink	monolink	55	n/a	-	F1905_sailerc	1566.766	523.263	3.000	0.2	1	21	0.46	39.23	0.000	sp|P02406|RL28_YEAST:55
AEKTDLTNILIR-K	sp|P39715|EC-	monolink	monolink	57	n/a	-	F1905_sailerc	1540.882	514.635	3.000	-0.1	1	18	0.69	39.19	0.000	sp|P39715|ECM1_YEAST:57
GPEIFNKYVGESE	sp|P32794|AF-	monolink	monolink	589	n/a	-	F1905_sailerc	1779.867	594.297	3.000	-0.4	9	22	0.45	39.17	0.000	sp|P32794|AFG2_YEAST:589
AVFKVKPVTR-K4-	sp|P25382|NLE-	monolink	monolink	128	n/a	-	F1905_sailerc	1299.790	434.271	3.000	-1.1	2	16	0.27	39.10	0.000	sp|P25382|NLE1_YEAST:128
KSIACVLTVINEQC	sp|P0CX84|RL3-	monolink	monolink	49	n/a	-	F1905_sailerc	1914.028	639.017	3.000	1.8	2	27	0.66	39.10	0.000	sp|P0CX84|RL35A_YEAST:49
SVTQESALDEFLS	sp|P53145|LSG-	monolink	monolink	86	n/a	-	F1905_sailerc	2955.433	986.152	3.000	-0.5	7	25	0.31	39.01	0.000	sp|P53145|LSG1_YEAST:86
SDYSRPNTKAPLT	sp|P33201|MR-	monolink	monolink	130	n/a	-	F1905_sailerc	2867.472	956.832	3.000	0.4	11	35	0.34	39.01	0.000	sp|P33201|MRT4_YEAST:130
KTVAGGAYTVSTA	sp|P0CX25|RL4-	monolink	monolink	62	n/a	-	F1905_sailerc	1950.043	651.022	3.000	0.7	2	20	0.51	39.01	0.000	sp|P0CX25|RL43A_YEAST:62
YVYAHFPINVNIV	sp|P05738|RL-	monolink	monolink	106,106	n/a	-	F1905_sailerc	2332.210	778.411	3.000	-0.3	6	23	0.38	39.00	0.000	sp|P05738|RL9A_YEAST:106:|:sp|P51401|RL9B_YEAST:106
DLETLTMHTKTYA	sp|P38061|RL3-	monolink	monolink	90	n/a	-	F1905_sailerc	2713.370	905.464	3.000	2.3	3	22	0.35	38.90	0.000	sp|P38061|RL32_YEAST:90
SVTQESALDEFLS	sp|P53145|LSG-	monolink	monolink	86	n/a	-	F1905_sailerc	2956.427	986.483	3.000	2.6	15	24	0.42	38.87	0.000	sp|P53145|LSG1_YEAST:86
GVVWTESHQEA	sp|Q03862|AR-	monolink	monolink	285	n/a	-	F1905_sailerc	3152.568	1051.864	3.000	1.7	5	23	0.45	38.86	0.000	sp|Q03862|ARX1_YEAST:285
DHGKETCTAYIHP	sp|P32794|AF-	monolink	monolink	46	n/a	-	F1905_sailerc	3586.707	897.685	4.000	-0.1	19	26	0.37	38.78	0.000	sp|P32794|AFG2_YEAST:46
LPAASLGDMVMA	sp|P0CX41|RL-	monolink	monolink	63	n/a	-	F1905_sailerc	1785.975	596.333	3.000	1.0	2	20	0.36	38.74	0.000	sp|P0CX41|RL23A_YEAST:63
DFAQTHILKLK-K9	sp|Q03862|AR-	monolink	monolink	371	n/a	-	F1905_sailerc	1467.846	490.290	3.000	0.5	4	19	0.40	38.73	0.000	sp|Q03862|ARX1_YEAST:371
ETAPVIDTLAAGY	sp|P02406|RL2-	monolink	monolink	111	n/a	-	F1905_sailerc	2072.143	691.722	3.000	1.2	2	25	0.70	38.70	0.000	sp|P02406|RL28_YEAST:111
FAKLHMGTDDVR	sp|P0C2H8|RL-	monolink	monolink	41	n/a	-	F1905_sailerc	1544.768	515.930	3.000	1.5	2	19	0.69	38.70	0.000	sp|P0C2H8|RL31A_YEAST:41
FAKLHMGTDDVR	sp|P0C2H8|RL-	monolink	monolink	41	n/a	-	F1905_sailerc	1543.784	515.603	3.000	1.7	2	21	0.47	38.67	0.000	sp|P0C2H8|RL31A_YEAST:41
DLKFPLPHR-K3-1	sp|P0CX23|RL-	monolink	monolink	149	n/a	-	F1905_sailerc	1277.714	426.912	3.000	0.2	2	16	0.00	38.59	0.000	sp|P0CX23|RL20A_YEAST:149
AFPKDDRSKPVAL	sp|P14126|RL3-	monolink	monolink	34	n/a	-	F1905_sailerc	2395.280	599.828	4.000	0.5	2	21	0.93	38.46	0.000	sp|P14126|RL3_YEAST:34
QALILKQFGIAASE	sp|P33201|MR-	monolink	monolink	204	n/a	-	F1905_sailerc	1918.096	640.373	3.000	1.8	2	22	0.47	38.44	0.000	sp|P33201|MRT4_YEAST:204
APKWYPSEDVAA	sp|Q02326|RL-	monolink	monolink	8	n/a	-	F1905_sailerc	1857.007	620.010	3.000	2.1	2	20	0.57	38.42	0.000	sp|Q02326|RL6A_YEAST:8
YGLNHVVALIENK	sp|P17076|RL8-	monolink	monolink	146	n/a	-	F1905_sailerc	1752.978	585.334	3.000	0.2	1	16	0.49	38.40	0.000	sp|P17076|RL8A_YEAST:146
ETAPVIDTLAAGY	sp|P02406|RL2-	monolink	monolink	111	n/a	-	F1905_sailerc	2071.159	691.394	3.000	1.1	2	22	0.59	38.39	0.000	sp|P02406|RL28_YEAST:111
RGPLVVYAEDNGI	sp|P10664|RL4-	monolink	monolink	217,217	n/a	-	F1905_sailerc	2124.207	709.077	3.000	0.9	1	20	0.64	38.33	0.000	sp|P10664|RL4A_YEAST:217:|:sp|P49626|RL4B_YEAST:217
LGISAPKGVLLYGF	sp|P32794|AF-	monolink	monolink	551	n/a	-	F1905_sailerc	2068.141	690.388	3.000	1.1	4	22	0.61	38.32	0.000	sp|P32794|AFG2_YEAST:551
TRDLDLIYNDLSTK	sp|Q08004|BU-	monolink	monolink	28	n/a	-	F1905_sailerc	2392.250	798.425	3.000	0.7	1	18	0.66	38.31	0.000	sp|Q08004|BUD20_YEAST:28
MASLEHCTKKGL	sp|Q03862|AR-	monolink	monolink	331	n/a	-	F1905_sailerc	2823.408	706.860	4.000	1.8	3	22	0.44	38.29	0.000	sp|Q03862|ARX1_YEAST:331
SLVDNMITGVTK	sp|P05738|RL-	monolink	monolink	84,84	n/a	-	F1905_sailerc	1780.930	594.651	3.000	0.9	3	24	0.48	38.28	0.000	sp|P05738|RL9A_YEAST:84:|:sp|P51401|RL9B_YEAST:84
SIHILKVAEIEK-K6	sp|P0CX23|RL-	monolink	monolink	125	n/a	-	F1905_sailerc	1534.896	512.640	3.000	-0.5	2	17	0.47	38.28	0.000	sp|P0CX23|RL20A_YEAST:125
NTAAETFKLFNK-	sp|P17076|RL8-	monolink	monolink	92,92	n/a	-	F1905_sailerc	1538.800	513.941	3.000	0.9	2	20	0.58	38.22	0.000	sp|P17076|RL8A_YEAST:92:|:sp|P29453|RL8B_YEAST:92
ALATESGINFLAV	sp|P32794|AF-	monolink	monolink	589	n/a	-	F1905_sailerc	3194.662	1065.895	3.000	3.9	10	23	0.37	38.14	0.000	sp|P32794|AFG2_YEAST:589
KSIACVLTVINEQC	sp|P0CX84|RL3-	monolink	monolink	49	n/a	-	F1905_sailerc	1913.042	638.688	3.000	0.7	3	24	0.54	38.11	0.000	sp|P0CX84|RL35A_YEAST:49
LGISAPKGVLLYGF	sp|P32794|AF-	monolink	monolink	551	n/a	-	F1905_sailerc	2069.126	690.716	3.000	1.2	9	22	0.55	38.10	0.000	sp|P32794|AFG2_YEAST:551
GILLHGPPGTGKT	sp|P32794|AF-	monolink	monolink	292	n/a	-	F1905_sailerc	1916.092	639.705	3.000	0.4	5	25	0.59	38.01	0.000	sp|P32794|AFG2_YEAST:292
KKVMPAIVVR-K1	sp|P0CX41|RL-	monolink	monolink	71	n/a	-	F1905_sailerc	1294.816	432.613	3.000	-0.1	1	15	0.00	38.00	0.000	sp|P0CX41|RL23A_YEAST:71
AFPKDDRSKPVAL	sp|P14126|RL3-	monolink	monolink	39	n/a	-	F1905_sailerc	2395.285	599.829	4.000	2.5	3	18	0.94	37.96	0.000	sp|P14126|RL3_YEAST:39
AEAKAQGVAVQL	sp|Q02753|RL-	monolink	monolink	120,120	n/a	-	F1905_sailerc	1466.845	489.956	3.000	-0.2	1	17	0.40	37.78	0.000	sp|Q02753|RL21A_YEAST:120:|:sp|Q12672|RL21B_YEAST:120
EIFLEMPKVYWSD	sp|P32794|AF-	monolink	monolink	512	n/a	-	F1905_sailerc	2665.339	889.454	3.000	1.7	6	26	0.44	37.76	0.000	sp|P32794|AFG2_YEAST:512
KHEEDEEESAKPA	sp|Q06205|FK-	monolink	monolink	244	n/a	-	F1905_sailerc	1910.874	637.966	3.000	0.0	6	19	0.39	37.73	0.000	sp|Q06205|FKBP4_YEAST:244
LSHLSSNFPFVHE	sp|Q03862|AR-	monolink	monolink	413	n/a	-	F1905_sailerc	2867.438	956.820	3.000	1.2	8	21	0.40	37.69	0.000	sp|Q03862|ARX1_YEAST:413
TAGQIAQTALKYV	sp|Q03862|AR-	monolink	monolink	40	n/a	-	F1905_sailerc	2763.448	691.870	4.000	1.4	8	20	0.52	37.63	0.000	sp|Q03862|ARX1_YEAST:40
ALKQEGAANKTV	sp|P0CX49|RL1-	monolink	monolink	72	n/a	-	F1905_sailerc	2496.363	833.129	3.000	3.2	2	25	0.46	37.59	0.000	sp|P0CX49|RL18A_YEAST:72
QATKDAGTIAGL	sp|P10591|HS-	monolink	monolink	157	n/a	-	F1905_sailerc	1782.003	595.009	3.000	1.5	2	20	0.70	37.51	0.000	sp|P10591|HSP71_YEAST:157
IKPFIKVVNYNHLL	sp|P0C2H6|RL-	monolink	monolink	69,69	n/a	-	F1905_sailerc	2207.285	552.829	4.000	0.9	1	19	0.58	37.48	0.000	sp|P0C2H6|RL27A_YEAST:69:|:sp|P0C2H7|RL27B_YEAST:69
EIESLKSAIEIPLHQ	sp|P32794|AF-	monolink	monolink	258	n/a	-	F1905_sailerc	3234.728	809.690	4.000	3.2	19	18	0.46	37.47	0.000	sp|P32794|AFG2_YEAST:258
VYWSDIGGQEEL	sp|P32794|AF-	monolink	monolink	527	n/a	-	F1905_sailerc	2167.089	723.371	3.000	1.1	4	23	0.68	37.46	0.000	sp|P32794|AFG2_YEAST:527
LFHSIKPLFANK-K	sp|Q02892|NC-	monolink	monolink	275	n/a	-	F1905_sailerc	1569.890	524.305	3.000	-1.0	4	19	0.62	37.46	0.000	sp|Q02892|NOG1_YEAST:275
ANHLSDYVKYLQ	sp|Q06338|BC-	monolink	monolink	128	n/a	-	F1905_sailerc	1732.917	578.647	3.000	1.1	3	15	0.71	37.45	0.000	sp|Q06338|BCP1_YEAST:128
TKFLSPSGHKTFL	sp|P38061|RL3-	monolink	monolink	72	n/a	-	F1905_sailerc	2129.188	533.305	4.000	-0.0	1	18	0.74	37.38	0.000	sp|P38061|RL32_YEAST:72
GRIPNVPVIVKAR-	sp|P02406|RL2-	monolink	monolink	126	n/a	-	F1905_sailerc	1573.966	525.663	3.000	-0.5	1	20	0.44	37.38	0.000	sp|P02406|RL28_YEAST:126
LNRLPAASLGDM	sp|P0CX41|RL-	monolink	monolink	63	n/a	-	F1905_sailerc	2169.203	724.076	3.000	0.8	2	19	0.42	37.30	0.000	sp|P0CX41|RL23A_YEAST:63
LSRPSLPKIK-K8-1	sp|P0CX84|RL3-	monolink	monolink	43	n/a	-	F1905_sailerc	1292.820	431.948	3.000	1.7	6	15	0.39	37.22	0.000	sp|P0CX84|RL35A_YEAST:43
LEILKKCTK-K5-15	sp|P32794|AF-	monolink	monolink	694	n/a	-	F1905_sailerc	1287.748	430.257	3.000	0.9	2	12	0.55	37.18	0.000	sp|P32794|AFG2_YEAST:694
LLDDSGVIMPGM	sp|P32794|AF-	monolink	monolink	170	n/a	-	F1905_sailerc	2375.247	792.757	3.000	-0.7	4	18	0.47	37.09	0.000	sp|P32794|AFG2_YEAST:170
GQSAGKLAAIVEII	sp|P36105|RL1-	monolink	monolink	31,31	n/a	-	F1905_sailerc	1895.074	632.699	3.000	0.6	2	24	0.62	37.08	0.000	sp|P36105|RL14A_YEAST:31:|:sp|P38754|RL14B_YEAST:31
KGAGSIFTSHTR-K	sp|P0CX45|RL-	monolink	monolink	10	n/a	-	F1905_sailerc	1415.752	472.925	3.000	-0.1	2	20	0.34	37.06	0.000	sp|P0CX45|RL2A_YEAST:10
WAAAAVCEKWA	sp|P36105|RL1-	monolink	monolink	93	n/a	-	F1905_sailerc	2047.003	683.342	3.000	1.9	4	20	0.46	37.01	0.000	sp|P36105|RL14A_YEAST:93
ALATESGINFLAV	sp|P32794|AF-	monolink	monolink	582	n/a	-	F1905_sailerc	2373.292	792.105	3.000	-1.2	4	28	0.39	36.93	0.000	sp|P32794|AFG2_YEAST:582
FQLDRQEKEAFLE	sp|P53145|LSG-	monolink	monolink	156	n/a	-	F1905_sailerc	2150.083	717.702	3.000	1.7	2	18	0.60	36.92	0.000	sp|P53145|LSG1_YEAST:156
VFGALKGASDGGI	sp|P26321|RL5-	monolink	monolink	164	n/a	-	F1905_sailerc	2329.167	777.397	3.000	-1.5	12	25	0.42	36.91	0.000	sp|P26321|RL5_YEAST:164
QAYAVSEKAGHQ	sp|P10664|RL4-	monolink	monolink	55,55	n/a	-	F1905_sailerc	2476.165	826.396	3.000	1.4	4	22	0.35	36.90	0.000	sp|P10664|RL4A_YEAST:55:|:sp|P49626|RL4B_YEAST:55
DGKAPEAEQVLS	sp|P40212|RL1-	monolink	monolink	131	n/a	-	F1905_sailerc	3108.563	778.149	4.000	0.4	29	26	0.45	36.84	0.000	sp|P40212|RL13B_YEAST:131
SKEQLASQLVDLK	sp|P0CX84|RL3-	monolink	monolink	25	n/a	-	F1905_sailerc	1741.002	581.342	3.000	2.0	2	20	0.59	36.79	0.000	sp|P0CX84|RL35A_YEAST:25
LATRPKCGDCGS	sp|P87262|RL3-	monolink	monolink	43,43	n/a	-	F1905_sailerc	2669.387	668.355	4.000	4.8	7	19	0.51	36.76	0.000	sp|P40525|RL34B_YEAST:43:|:sp|P87262|RL34A_YEAST:43
ALTKFEASQVTEK	sp|P0CX84|RL3-	monolink	monolink	103	n/a	-	F1905_sailerc	1891.006	631.343	3.000	0.5	3	19	0.39	36.73	0.000	sp|P0CX84|RL35A_YEAST:103
KKVMPAIVVR-K2	sp|P0CX41|RL-	monolink	monolink	72	n/a	-	F1905_sailerc	1294.816	432.613	3.000	0.0	1	15	0.00	36.70	0.000	sp|P0CX41|RL23A_YEAST:72
SLMNSTKESISLP	sp|Q03862|AR-	monolink	monolink	493	n/a	-	F1905_sailerc	1887.999	630.341	3.000	0.8	6	25	0.52	36.69	0.000	sp|Q03862|ARX1_YEAST:493
RLPTESVPEPKLFR	sp|P0CX23|RL-	monolink	monolink	23	n/a	-	F1905_sailerc	1824.016	609.013	3.000	0.8	3	17	0.57	36.63	0.000	sp|P0CX23|RL20A_YEAST:23
DLKFPLPHR-K3-1	sp|P0CX23|RL-	monolink	monolink	149	n/a	-	F1905_sailerc	1276.729	426.584	3.000	0.1	2	16	0.00	36.60	0.000	sp|P0CX23|RL20A_YEAST:149
KYQPSIIFIDEIDSI	sp|P32794|AF-	monolink	monolink	336	n/a	-	F1905_sailerc	2374.246	792.423	3.000	1.7	8	22	0.59	36.57	0.000	sp|P32794|AFG2_YEAST:336
VIQKLLDDSGVIM	sp|P32794|AF-	monolink	monolink	150	n/a	-	F1905_sailerc	2613.429	872.151	3.000	0.5	3	26	0.39	36.54	0.000	sp|P32794|AFG2_YEAST:150
VAEIEKTADVKR-	sp|P0CX23|RL-	monolink	monolink	131	n/a	-	F1905_sailerc	1512.850	505.291	3.000	-0.9	1	17	0.56	36.53	0.000	sp|P0CX23|RL20A_YEAST:131
FQTPAEKHAFMG	sp|P14126|RL3-	monolink	monolink	376	n/a	-	F1905_sailerc	1988.056	498.022	4.000	0.4	2	17	0.71	36.50	0.000	sp|P14126|RL3_YEAST:376
YVYAHFPINVNIV	sp|P05738|RL-	monolink	monolink	106,106	n/a	-	F1905_sailerc	2331.224	778.083	3.000	-1.0	2	21	0.37	36.46	0.000	sp|P05738|RL9A_YEAST:106:|:sp|P51401|RL9B_YEAST:106
LKEYQSKIIVFPR-	sp|Q12690|RL-	monolink	monolink	122,122	n/a	-	F1905_sailerc	1776.020	593.014	3.000	0.8	2	18	0.49	36.44	0.000	sp|P40212|RL13B_YEAST:122:|:sp|Q12690|RL13A_YEAST:122
TSFFQALGVPTKI	sp|P05317|RL-	monolink	monolink	144	n/a	-	F1905_sailerc	1790.994	598.006	3.000	0.7	3	21	0.51	36.41	0.000	sp|P05317|RLA0_YEAST:144
YNAFIASEVLIKQV	sp|P0CX43|RL1-	monolink	monolink	118	n/a	-	F1905_sailerc	2003.110	668.711	3.000	0.5	2	21	0.53	36.37	0.000	sp|P0CX43|RL1A_YEAST:118
RVAQLPPISFETFD	sp|P38344|REI-	monolink	monolink	53	n/a	-	F1905_sailerc	2863.496	955.507	3.000	-0.2	4	24	0.48	36.37	0.000	sp|P38344|REI1_YEAST:53
LLPFVKGNVGFVF	sp|P05317|RL-	monolink	monolink	81	n/a	-	F1905_sailerc	2617.446	873.490	3.000	2.1	2	20	0.58	36.35	0.000	sp|P05317|RLA0_YEAST:81
SVTQESALDEFLS	sp|P53145|LSG-	monolink	monolink	86	n/a	-	F1905_sailerc	3521.712	881.436	4.000	-1.1	2	21	0.48	36.35	0.000	sp|P53145|LSG1_YEAST:86
KSELEYYAMLSKTI	sp|P14120|RL3-	monolink	monolink	64	n/a	-	F1905_sailerc	1845.944	616.322	3.000	0.1	2	17	0.42	36.34	0.000	sp|P14120|RL30_YEAST:64
APKWYPSEDVAA	sp|Q02326|RL-	monolink	monolink	8	n/a	-	F1905_sailerc	1857.986	620.336	3.000	-0.9	2	19	0.50	36.33	0.000	sp|Q02326|RL6A_YEAST:8
GIVKQIVHDSGR-	sp|P0CX45|RL-	monolink	monolink	46	n/a	-	F1905_sailerc	1463.809	488.944	3.000	-0.5	3	19	0.28	36.31	0.000	sp|P0CX45|RL2A_YEAST:46
KYNAFIASEVLIKQ	sp|P0CX43|RL1-	monolink	monolink	118	n/a	-	F1905_sailerc	2131.207	711.410	3.000	1.4	3	22	0.59	36.22	0.000	sp|P0CX43|RL1A_YEAST:118
ASGNYVIIIGHNP	sp|P0CX45|RL-	monolink	monolink	145	n/a	-	F1905_sailerc	2252.156	751.726	3.000	0.5	3	23	0.48	36.21	0.000	sp|P0CX45|RL2A_YEAST:145
SGTHNMYKEIR-K	sp|P0CX23|RL-	monolink	monolink	92	n/a	-	F1905_sailerc	1490.720	497.915	3.000	0.9	1	17	0.00	36.21	0.000	sp|P0CX23|RL20A_YEAST:92
GVLLYGPPGCSKT	sp|P32794|AF-	monolink	monolink	568	n/a	-	F1905_sailerc	3331.801	1111.608	3.000	-2.7	4	24	0.39	36.14	0.000	sp|P32794|AFG2_YEAST:568
TAGQIAQTALKYV	sp|Q03862|AR-	monolink	monolink	40	n/a	-	F1905_sailerc	2764.428	692.115	4.000	-0.1	17	19	0.54	36.07	0.000	sp|Q03862|ARX1_YEAST:40
LNRLPAASLGDM	sp|P0CX41|RL-	monolink	monolink	63	n/a	-	F1905_sailerc	2170.188	724.404	3.000	1.4	4	20	0.43	36.07	0.000	sp|P0CX41|RL23A_YEAST:63
SDYSRPNTKAPLT	sp|P33201|MR-	monolink	monolink	130	n/a	-	F1905_sailerc	2866.486	956.503	3.000	-0.0	4	28	0.42	36.06	0.000	sp|P33201|MRT4_YEAST:130
SAEKEKPVTKK-K6	sp|P38344|REI-	monolink	monolink	69	n/a	-	F1905_sailerc	1399.792	467.605	3.000	-0.4	1	13	0.44	36.06	0.000	sp|P38344|REI1_YEAST:69
LPAASLGDMVMA	sp|P0CX41|RL-	monolink	monolink	64	n/a	-	F1905_sailerc	1786.958	596.661	3.000	0.6	1	17	0.45	36.06	0.000	sp|P0CX41|RL23A_YEAST:64
VIQKLLDDSGVIM	sp|P32794|AF-	monolink	monolink	150	n/a	-	F1905_sailerc	2614.417	872.480	3.000	2.3	9	22	0.42	35.97	0.000	sp|P32794|AFG2_YEAST:150
QATKDAGTIAGL	sp|P10591|HS-	monolink	monolink	157	n/a	-	F1905_sailerc	1782.985	595.336	3.000	0.3	2	17	0.69	35.94	0.000	sp|P10591|HSP71_YEAST:157
YNLPEPLSYAAVG	sp|P32794|AF-	monolink	monolink	252	n/a	-	F1905_sailerc	2660.403	887.809	3.000	3.0	4	25	0.65	35.90	0.000	sp|P32794|AFG2_YEAST:252
LSTPFHKVQNSSA	sp|P53145|LSG-	monolink	monolink	611	n/a	-	F1905_sailerc	1912.020	638.348	3.000	1.5	4	22	0.74	35.83	0.000	sp|P53145|LSG1_YEAST:611
KVVIVKPHDEGSK	sp|P0C2H6|RL-	monolink	monolink	22,22	n/a	-	F1905_sailerc	1590.899	531.308	3.000	0.6	3	18	0.65	35.82	0.000	sp|P0C2H6|RL27A_YEAST:22:|:sp|P0C2H7|RL27B_YEAST:22
AGKFPTPVSHND	sp|P0CX43|RL1-	monolink	monolink	147	n/a	-	F1905_sailerc	2571.299	858.107	3.000	0.8	5	20	0.47	35.81	0.000	sp|P0CX43|RL1A_YEAST:147
KYQPSIIFIDEIDSI	sp|P32794|AF-	monolink	monolink	336	n/a	-	F1905_sailerc	3533.722	884.438	4.000	1.7	23	23	0.33	35.79	0.000	sp|P32794|AFG2_YEAST:336
YGLNHVVALIENK	sp|P17076|RL8-	monolink	monolink	147	n/a	-	F1905_sailerc	1752.980	585.334	3.000	1.4	1	16	0.42	35.71	0.000	sp|P17076|RL8A_YEAST:147
MAKEHKDIDASL	sp|P38861|NM-	monolink	monolink	419	n/a	-	F1905_sailerc	2148.016	717.013	3.000	0.2	2	20	0.30	35.66	0.000	sp|P38861|NMD3_YEAST:419
SKEQLASQLVDLK	sp|P0CX84|RL3-	monolink	monolink	26	n/a	-	F1905_sailerc	1740.999	581.341	3.000	0.1	1	16	0.56	35.64	0.000	sp|P0CX84|RL35A_YEAST:26
YGLNHVVSLIENK	sp|P29453|RL8-	monolink	monolink	146	n/a	-	F1905_sailerc	1767.990	590.338	3.000	1.2	2	19	0.54	35.63	0.000	sp|P29453|RL8B_YEAST:146
AVGAHSDLLKVLK	sp|P10664|RL4-	monolink	monolink	180,180	n/a	-	F1905_sailerc	1505.881	502.968	3.000	-0.6	2	17	0.62	35.61	0.000	sp|P10664|RL4A_YEAST:180:|:sp|P49626|RL4B_YEAST:180
IREGDTKDGNLR-	sp|P53742|NO-	monolink	monolink	18	n/a	-	F1905_sailerc	1528.785	510.603	3.000	0.0	2	16	0.36	35.59	0.000	sp|P53742|NOG2_YEAST:18
GVIGVIAGGGRV	sp|P0CX45|RL-	monolink	monolink	181	n/a	-	F1905_sailerc	2188.310	730.445	3.000	2.1	3	22	0.54	35.55	0.000	sp|P0CX45|RL2A_YEAST:181
TKSLIANIHR-K2-1	sp|P40991|NO-	monolink	monolink	384	n/a	-	F1905_sailerc	1307.756	436.927	3.000	0.2	1	13	0.44	35.48	0.000	sp|P40991|NOP2_YEAST:384
FVQGLLQNAAAN	sp|P05740|RL1-	monolink	monolink	105,105	n/a	-	F1905_sailerc	2385.258	796.094	3.000	1.9	3	27	0.49	35.35	0.000	sp|P05740|RL17A_YEAST:105:|:sp|P46990|RL17B_YEAST:105
KGLVTLETVDSYT	sp|Q03862|AR-	monolink	monolink	332	n/a	-	F1905_sailerc	1764.953	589.326	3.000	1.4	2	20	0.50	35.17	0.000	sp|Q03862|ARX1_YEAST:332
DLDLIYNDLSTKES	sp|Q08004|BU-	monolink	monolink	28	n/a	-	F1905_sailerc	2135.105	712.709	3.000	2.2	6	21	0.69	35.08	0.000	sp|Q08004|BUD20_YEAST:28
SHKAGELIARPGA	sp|Q03862|AR-	monolink	monolink	349	n/a	-	F1905_sailerc	1879.044	470.769	4.000	0.6	2	16	0.71	35.06	0.000	sp|Q03862|ARX1_YEAST:349
YMETAIALKTHLK	sp|Q08004|BU-	monolink	monolink	65	n/a	-	F1905_sailerc	1673.907	558.977	3.000	0.5	4	19	0.70	34.78	0.000	sp|Q08004|BUD20_YEAST:65
TAAKNEALLEAKI	sp|P39715|EC-	monolink	monolink	78	n/a	-	F1905_sailerc	1741.985	581.669	3.000	1.1	2	17	0.81	34.77	0.000	sp|P39715|ECM1_YEAST:78
GSTHITFSKETQA	sp|P32794|AF-	monolink	monolink	228	n/a	-	F1905_sailerc	1959.019	654.014	3.000	0.7	8	16	0.71	34.76	0.000	sp|P32794|AFG2_YEAST:228
AKNPLTHSTPK-K2	sp|P29453|RL8-	monolink	monolink	23	n/a	-	F1905_sailerc	1348.735	450.586	3.000	0.1	3	16	0.16	34.75	0.000	sp|P29453|RL8B_YEAST:23
KYEVNFDTDDDT	sp|Q06338|BC-	monolink	monolink	213	n/a	-	F1905_sailerc	2158.970	720.664	3.000	1.9	2	17	0.00	34.61	0.000	sp|Q06338|BCP1_YEAST:213
HIDVTFTKVNNQ	sp|P05738|RL-	monolink	monolink	47,47	n/a	-	F1905_sailerc	1925.063	642.696	3.000	0.6	2	23	0.58	34.58	0.000	sp|P05738|RL9A_YEAST:47:|:sp|P51401|RL9B_YEAST:47
NEKIAVHVTVR-K	sp|P0C0W9|RL-	monolink	monolink	64,64	n/a	-	F1905_sailerc	1420.805	474.609	3.000	0.7	2	14	0.44	34.58	0.000	sp|P0C0W9|RL11A_YEAST:64:|:sp|Q3E757|RL11B_YEAST:64
VYWSDIGGQEEL	sp|P32794|AF-	monolink	monolink	525	n/a	-	F1905_sailerc	2167.087	723.370	3.000	0.1	2	21	0.86	34.57	0.000	sp|P32794|AFG2_YEAST:525
LIQSLPPTLKVNRL	sp|P49167|RL3-	monolink	monolink	74	n/a	-	F1905_sailerc	1747.060	583.361	3.000	-0.4	2	16	0.49	34.41	0.000	sp|P49167|RL38_YEAST:74
WAAAGVCEKWA	sp|P38754|RL1-	monolink	monolink	93	n/a	-	F1905_sailerc	2032.985	678.670	3.000	1.1	1	20	0.61	34.36	0.000	sp|P38754|RL14B_YEAST:93
TVKAQRPITGASL	sp|P04449|RL2-	monolink	monolink	77,77	n/a	-	F1905_sailerc	1966.150	656.391	3.000	2.0	4	19	0.56	34.34	0.000	sp|P04449|RL24A_YEAST:77:|:sp|P24000|RL24B_YEAST:77
QSDALEPEVKDLS	sp|P39715|EC-	monolink	monolink	47	n/a	-	F1905_sailerc	2081.052	694.692	3.000	-0.2	2	19	0.49	34.09	0.000	sp|P39715|ECM1_YEAST:47
TISDKNKAQLLIED	sp|Q12019|MD-	monolink	monolink	1978	n/a	-	F1905_sailerc	2188.203	730.409	3.000	1.9	1	19	0.46	34.03	0.000	sp|Q12019|MDN1_YEAST:1978
KVVKEQTEAAK-K	sp|P87262|RL3-	monolink	monolink	103,103	n/a	-	F1905_sailerc	1385.778	462.934	3.000	0.7	2	17	0.55	33.92	0.000	sp|P40525|RL34B_YEAST:103:|:sp|P87262|RL34A_YEAST:103
KAEKTGTKPAAVF	sp|P10664|RL4-	monolink	monolink	346	n/a	-	F1905_sailerc	2075.153	519.796	4.000	0.7	1	17	0.75	33.85	0.000	sp|P10664|RL4A_YEAST:346
ANMLENMQKSQ	sp|P38344|REI-	monolink	monolink	105	n/a	-	F1905_sailerc	2390.109	797.711	3.000	-0.2	4	24	0.43	33.74	0.000	sp|P38344|REI1_YEAST:105
LFNKYRPETAAEK	sp|P17076|RL8-	monolink	monolink	96,96	n/a	-	F1905_sailerc	1849.009	463.260	4.000	-0.5	5	19	0.76	33.68	0.000	sp|P17076|RL8A_YEAST:96:|:sp|P29453|RL8B_YEAST:96
GVLLYGPPGCSKT	sp|P32794|AF-	monolink	monolink	563	n/a	-	F1905_sailerc	3331.811	833.961	4.000	0.3	8	19	0.64	33.59	0.000	sp|P32794|AFG2_YEAST:563
LLKFGQSLFQCK-K	sp|Q02892|NC-	monolink	monolink	117	n/a	-	F1905_sailerc	1623.872	542.298	3.000	1.4	1	17	0.56	33.58	0.000	sp|Q02892|NOG1_YEAST:117
AAGSYYVEAQHK	sp|P05737|RL7-	monolink	monolink	82,82	n/a	-	F1905_sailerc	2192.166	731.730	3.000	1.5	4	19	0.67	33.54	0.000	sp|P05737|RL7A_YEAST:82:|:sp|Q12213|RL7B_YEAST:82
VAQLPPISFETFDS	sp|P38344|REI-	monolink	monolink	53	n/a	-	F1905_sailerc	2707.403	903.476	3.000	2.8	14	19	0.47	33.48	0.000	sp|P38344|REI1_YEAST:53
VVANTSNAHVLTI	sp|P32794|AF-	monolink	monolink	318	n/a	-	F1905_sailerc	3378.820	845.713	4.000	1.4	6	19	0.55	33.48	0.000	sp|P32794|AFG2_YEAST:318
SVVSTETFEQPSQ	sp|P0C2H6|RL-	monolink	monolink	111,111	n/a	-	F1905_sailerc	2334.168	779.064	3.000	-0.8	1	19	0.35	33.47	0.000	sp|P0C2H6|RL27A_YEAST:111:|:sp|P0C2H7|RL27B_YEAST:111
SKQDASPKPYAVK	sp|P17076|RL8-	monolink	monolink	122,122	n/a	-	F1905_sailerc	1572.853	525.292	3.000	1.0	3	15	0.66	33.36	0.000	sp|P17076|RL8A_YEAST:122:|:sp|P29453|RL8B_YEAST:122
VVYLKHLEDNTLL	sp|P05739|RL6-	monolink	monolink	56	n/a	-	F1905_sailerc	2341.296	781.440	3.000	1.0	13	18	0.86	33.31	0.000	sp|P05739|RL6B_YEAST:56
GVPYTQEVSDAA	sp|Q08004|BU-	monolink	monolink	100	n/a	-	F1905_sailerc	2682.332	895.119	3.000	1.2	9	21	0.42	33.26	0.000	sp|Q08004|BUD20_YEAST:100
KYNLPEPLSYAAV	sp|P32794|AF-	monolink	monolink	252	n/a	-	F1905_sailerc	2788.494	930.506	3.000	1.7	18	22	0.67	33.21	0.000	sp|P32794|AFG2_YEAST:252
LVKNGTIVKK-K3-	sp|P0CX82|RL1-	monolink	monolink	46	n/a	-	F1905_sailerc	1253.808	418.944	3.000	0.4	2	14	0.67	33.12	0.000	sp|P0CX82|RL19A_YEAST:46
KVSVSSTPGKTK-	sp|P53145|LSG-	monolink	monolink	370	n/a	-	F1905_sailerc	1372.793	458.606	3.000	0.4	1	18	0.58	33.11	0.000	sp|P53145|LSG1_YEAST:370
SVGNLILGDRLEL	sp|P32794|AF-	monolink	monolink	117	n/a	-	F1905_sailerc	1809.074	604.033	3.000	1.0	5	19	0.70	33.02	0.000	sp|P32794|AFG2_YEAST:117
MASLEHCTKK-K9	sp|Q03862|AR-	monolink	monolink	331	n/a	-	F1905_sailerc	1359.653	454.225	3.000	-0.1	3	12	0.51	32.95	0.000	sp|Q03862|ARX1_YEAST:331
INMDKYHPGYFGI	sp|P02406|RL2-	monolink	monolink	47	n/a	-	F1905_sailerc	1723.840	575.621	3.000	0.2	2	19	0.68	32.94	0.000	sp|P02406|RL28_YEAST:47
KSEELISQDTHTG	sp|P38861|NM-	monolink	monolink	224	n/a	-	F1905_sailerc	2150.040	717.688	3.000	1.1	3	20	0.33	32.89	0.000	sp|P38861|NMD3_YEAST:224
SLTTVWAEHLSDE	sp|P14126|RL3-	monolink	monolink	115	n/a	-	F1905_sailerc	2026.039	676.354	3.000	0.7	2	14	0.63	32.76	0.000	sp|P14126|RL3_YEAST:115
RLPTESVPEPKLFR	sp|P0CX23|RL-	monolink	monolink	23	n/a	-	F1905_sailerc	1823.030	608.684	3.000	-0.4	4	16	0.63	32.69	0.000	sp|P0CX23|RL20A_YEAST:23
VQKLSRPSLPK-K3	sp|P0CX84|RL3-	monolink	monolink	35	n/a	-	F1905_sailerc	1407.843	470.289	3.000	-1.1	6	14	0.48	32.59	0.000	sp|P0CX84|RL35A_YEAST:35
KNESQNSTAVTLC	sp|P47019|ALE-	monolink	monolink	63	n/a	-	F1905_sailerc	2746.376	916.467	3.000	1.2	5	20	0.34	32.58	0.000	sp|P47019|ALB1_YEAST:63
DAKLDKSQVDEIV	sp|P10591|HS-	monolink	monolink	325	n/a	-	F1905_sailerc	2285.216	762.747	3.000	2.1	1	19	0.56	32.58	0.000	sp|P10591|HSP71_YEAST:325
TGTKPAAVFAETL	sp|P49626|RL4-	monolink	monolink	360	n/a	-	F1905_sailerc	1840.958	614.661	3.000	0.6	1	16	0.39	32.57	0.000	sp|P49626|RL4B_YEAST:360
AREKAEAEAEK-K	sp|Q12690|RL-	monolink	monolink	190,190	n/a	-	F1905_sailerc	1386.700	463.241	3.000	0.5	2	17	0.46	32.55	0.000	sp|P40212|RL13B_YEAST:190:|:sp|Q12690|RL13A_YEAST:190
SLYVGHFDYKYLR-	sp|Q02892|NC-	monolink	monolink	212	n/a	-	F1905_sailerc	1815.923	606.315	3.000	1.7	2	17	0.76	32.55	0.000	sp|Q02892|NOG1_YEAST:212
AVNTGMEPGKTS	sp|P05317|RL-	monolink	monolink	132	n/a	-	F1905_sailerc	2435.245	812.756	3.000	1.9	6	21	0.61	32.53	0.000	sp|P05317|RLA0_YEAST:132
ATNPNGNLSYDA	sp|Q03862|AR-	monolink	monolink	477	n/a	-	F1905_sailerc	3643.960	911.998	4.000	3.9	4	18	0.41	32.53	0.000	sp|Q03862|ARX1_YEAST:477
AKAKVEEMNNIIA	sp|P05745|RL3-	monolink	monolink	84,84	n/a	-	F1905_sailerc	1900.010	634.344	3.000	0.6	2	19	0.59	32.52	0.000	sp|O14455|RL36B_YEAST:84:|:sp|P05745|RL36A_YEAST:84
GVIGVIAGGGRV	sp|P0CX45|RL-	monolink	monolink	177	n/a	-	F1905_sailerc	2187.323	547.838	4.000	0.4	1	20	0.84	32.49	0.000	sp|P0CX45|RL2A_YEAST:177
AAGKELAVDSTLT	sp|Q07915|RL-	monolink	monolink	61	n/a	-	F1905_sailerc	1932.035	645.019	3.000	1.5	1	18	0.91	32.44	0.000	sp|Q07915|RLP24_YEAST:61
AKVEEMNNIIAAS	sp|P05745|RL3-	monolink	monolink	86,86	n/a	-	F1905_sailerc	1699.893	567.639	3.000	0.4	2	16	0.70	32.40	0.000	sp|O14455|RL36B_YEAST:86:|:sp|P05745|RL36A_YEAST:86
GPEIFNKYVGESE	sp|P32794|AF-	monolink	monolink	589	n/a	-	F1905_sailerc	1778.885	593.970	3.000	0.7	2	17	0.62	32.30	0.000	sp|P32794|AFG2_YEAST:589
YVTSLINDSYHSKT	sp|Q03862|AR-	monolink	monolink	53	n/a	-	F1905_sailerc	2168.083	723.702	3.000	3.7	6	19	0.73	32.25	0.000	sp|Q03862|ARX1_YEAST:53
GVLLYGPPGCSKT	sp|P32794|AF-	monolink	monolink	563	n/a	-	F1905_sailerc	1917.029	640.018	3.000	0.7	1	15	0.66	32.14	0.000	sp|P32794|AFG2_YEAST:563
EAVAALKAVGAH	sp|P10664|RL4-	monolink	monolink	170,170	n/a	-	F1905_sailerc	1848.038	617.021	3.000	1.3	6	17	0.67	32.10	0.000	sp|P10664|RL4A_YEAST:170:|:sp|P49626|RL4B_YEAST:170
VAQLPPISFETFDS	sp|P38344|REI-	monolink	monolink	53	n/a	-	F1905_sailerc	2706.417	903.147	3.000	2.1	4	22	0.52	32.04	0.000	sp|P38344|REI1_YEAST:53
GTLFVRGDSKIFR-	sp|P04449|RL2-	monolink	monolink	27,27	n/a	-	F1905_sailerc	1650.910	551.311	3.000	0.7	2	17	0.70	32.01	0.000	sp|P04449|RL24A_YEAST:27:|:sp|P24000|RL24B_YEAST:27

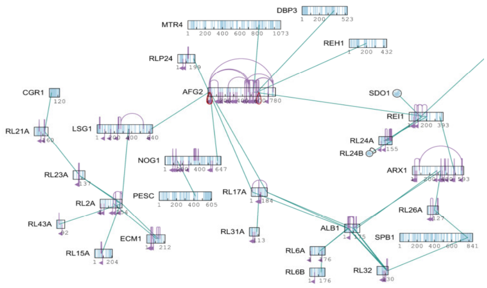

MTR4
DBP3
REH1
RLP24
AFG2
CGR1
SDO1
REI1
RL21A
LSG1
RL24A
RL24B
NOG1
ARX1
RL23A
PESC
RL17A
RL2A
RL26A
RL43A
RL31A
ALB1
SPB1
ECM1
RL15A
RL6A
RL6B
RL32
