# Supplementary figures and images for: Visualizing maturation factor extraction from the nascent ribosome by the AAA-ATPase Drg1
Source: Nat Struct Mol Biol. 2022 Sep 12;29(9):942–53. doi: 10.1038/s41594-022-00832-5 (PMC9507969; doi:10.1038/s41594-022-00832-5)

Figure1a

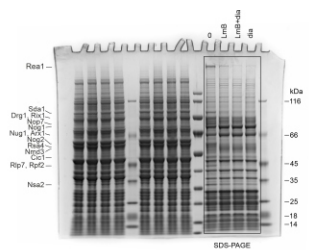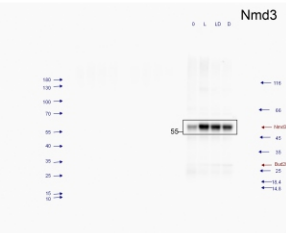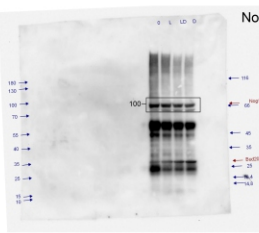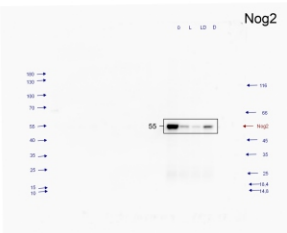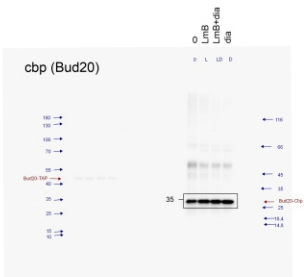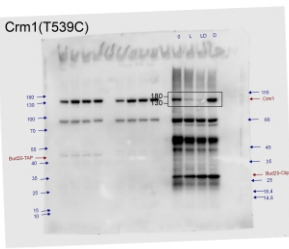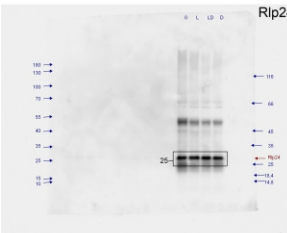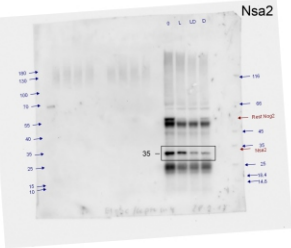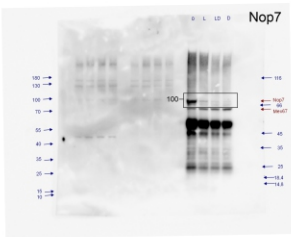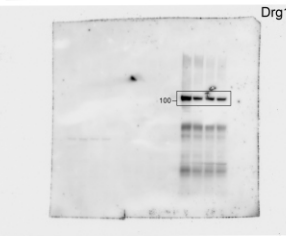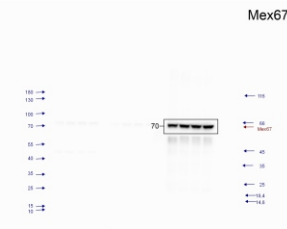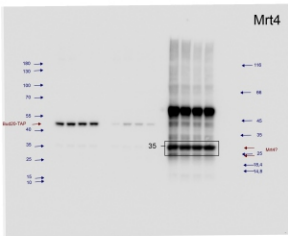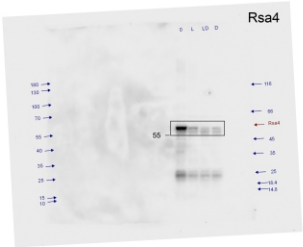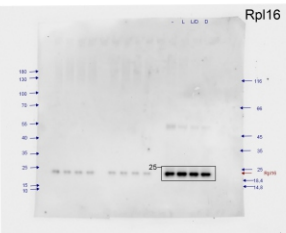

Figure1b

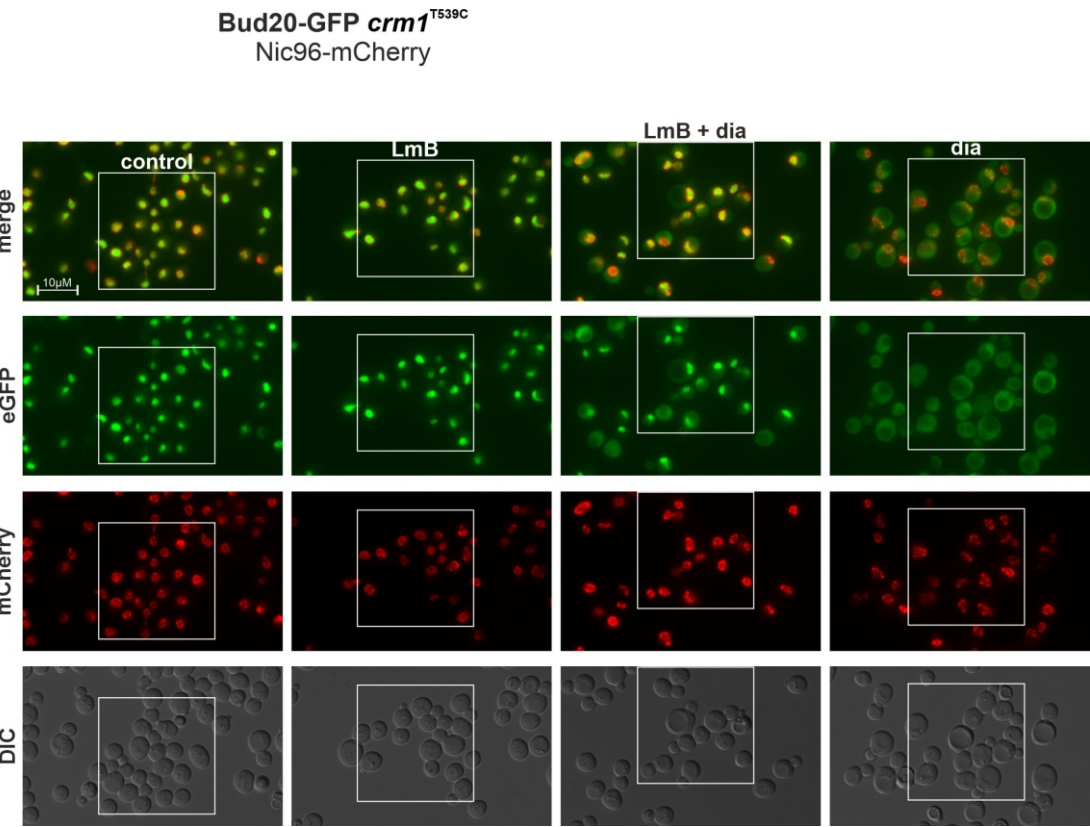

Supplement: Source Data Fig. 1 — Unprocessed Coomassie gel and Western blots and Uncropped microscopy images [file 41594_2022_832_MOESM7_ESM.pdf]

Fig.3c

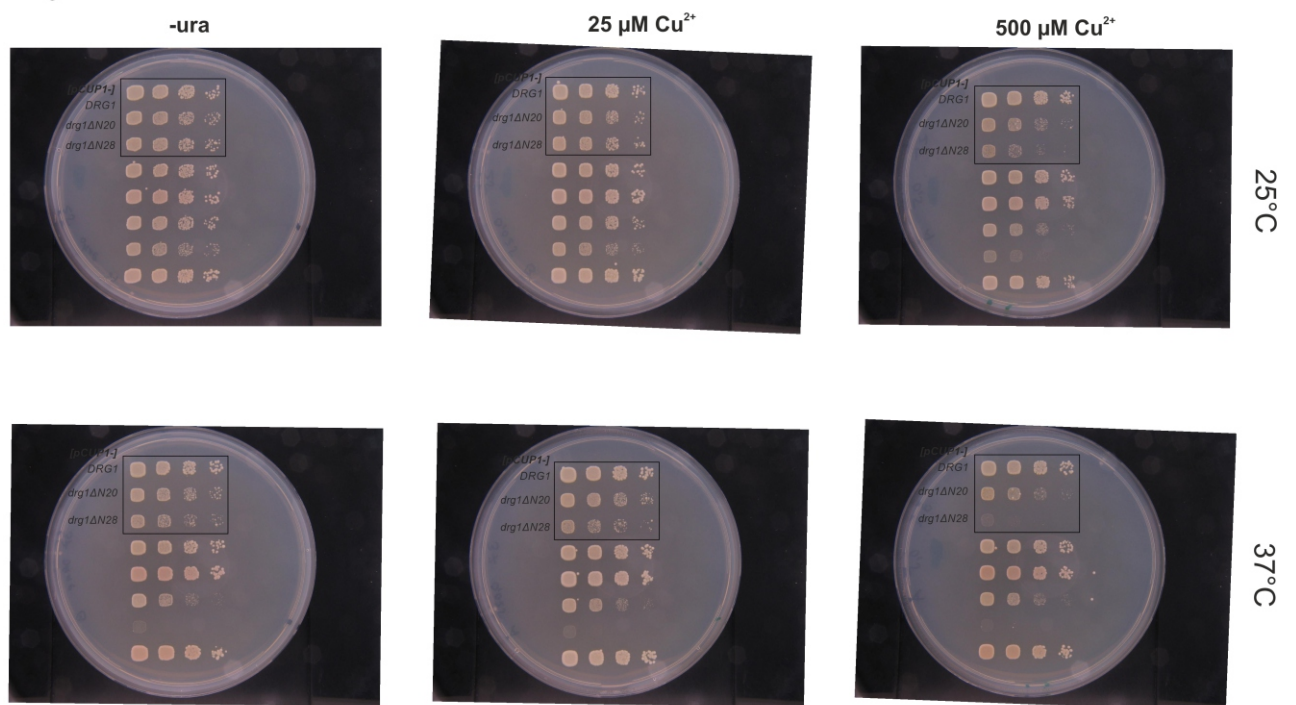

Fig.3d

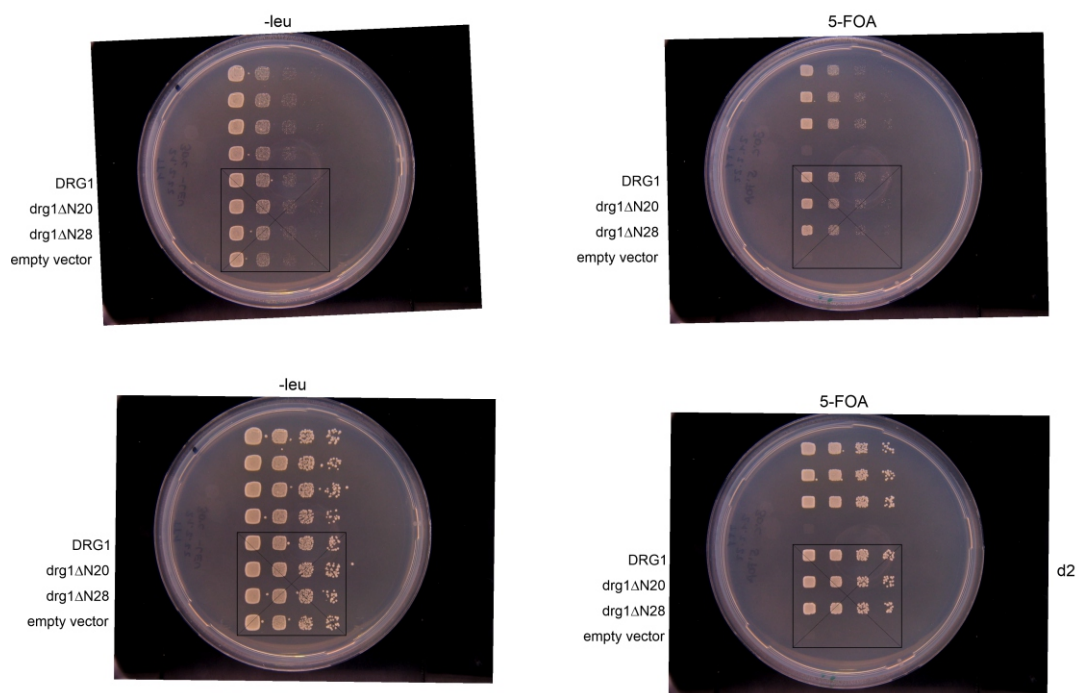

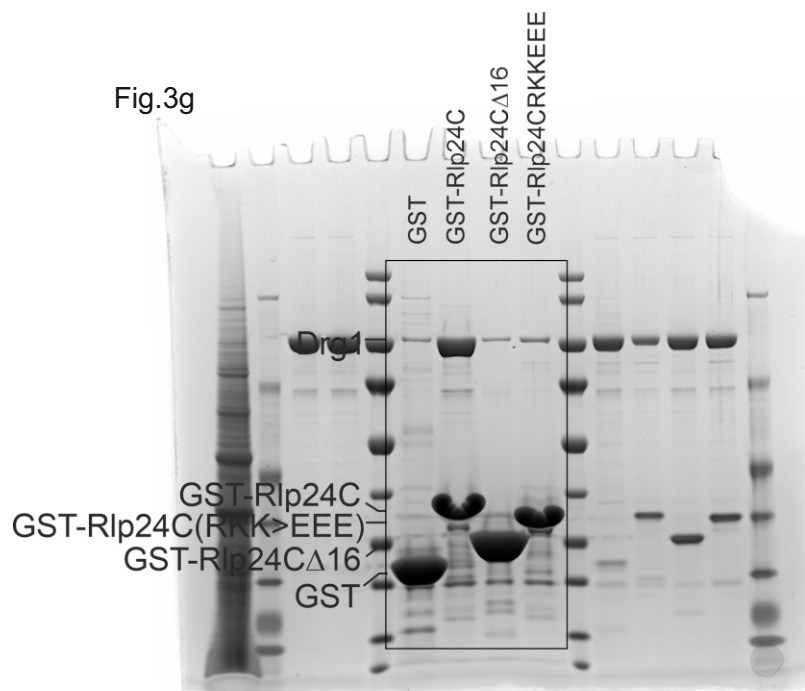

Fig.3h Rlp24ΔC expression  
(*rlp24Δ* background)

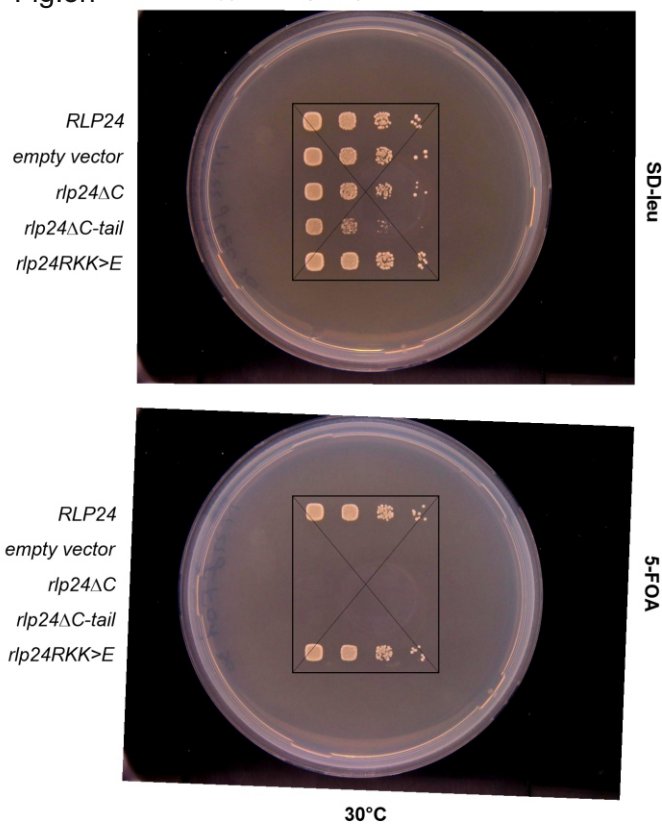

Supplement: Source Data Fig. 3 — Uncropped spot assay and Coomassie gel images [file 41594_2022_832_MOESM8_ESM.pdf]

## GST-Arx1 pulldown

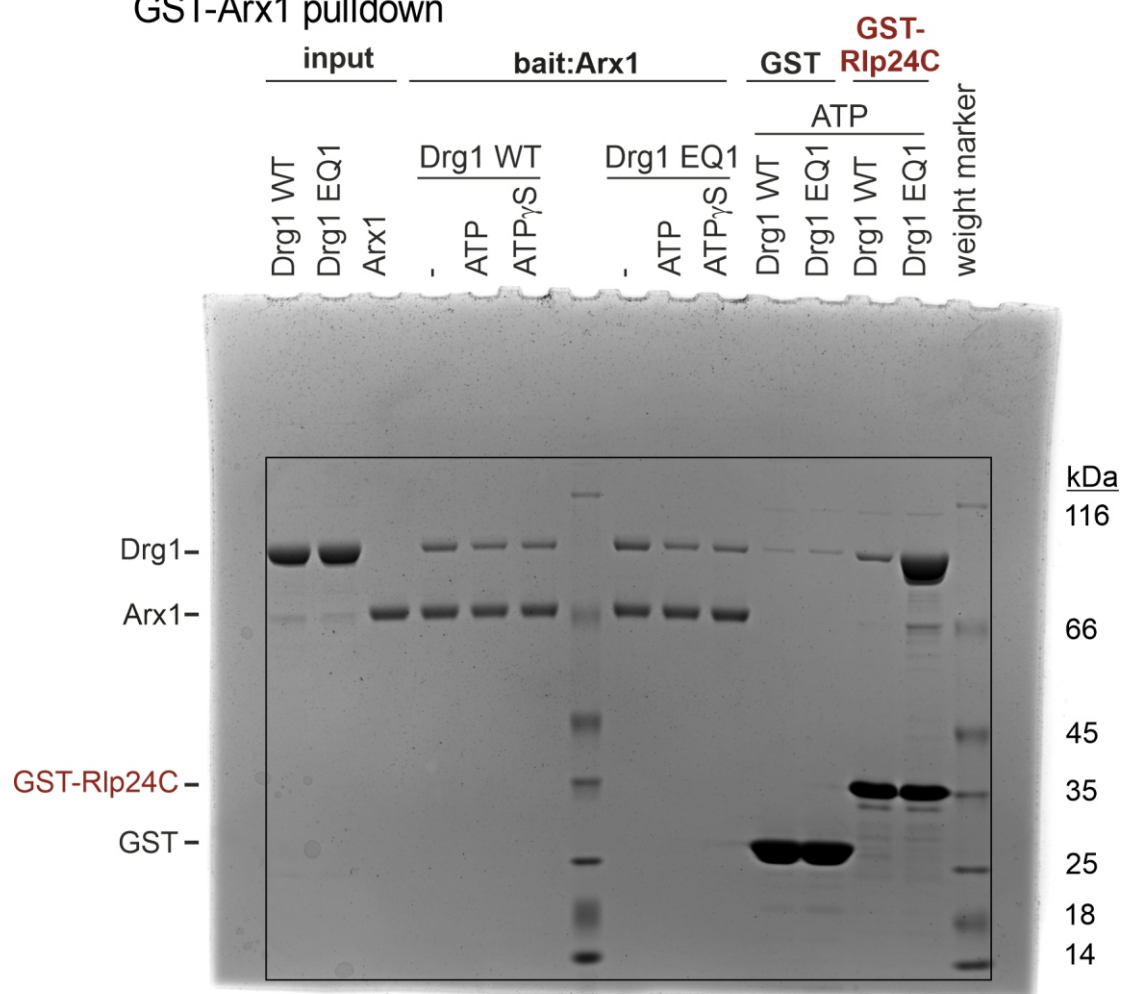

## ED Fig3d

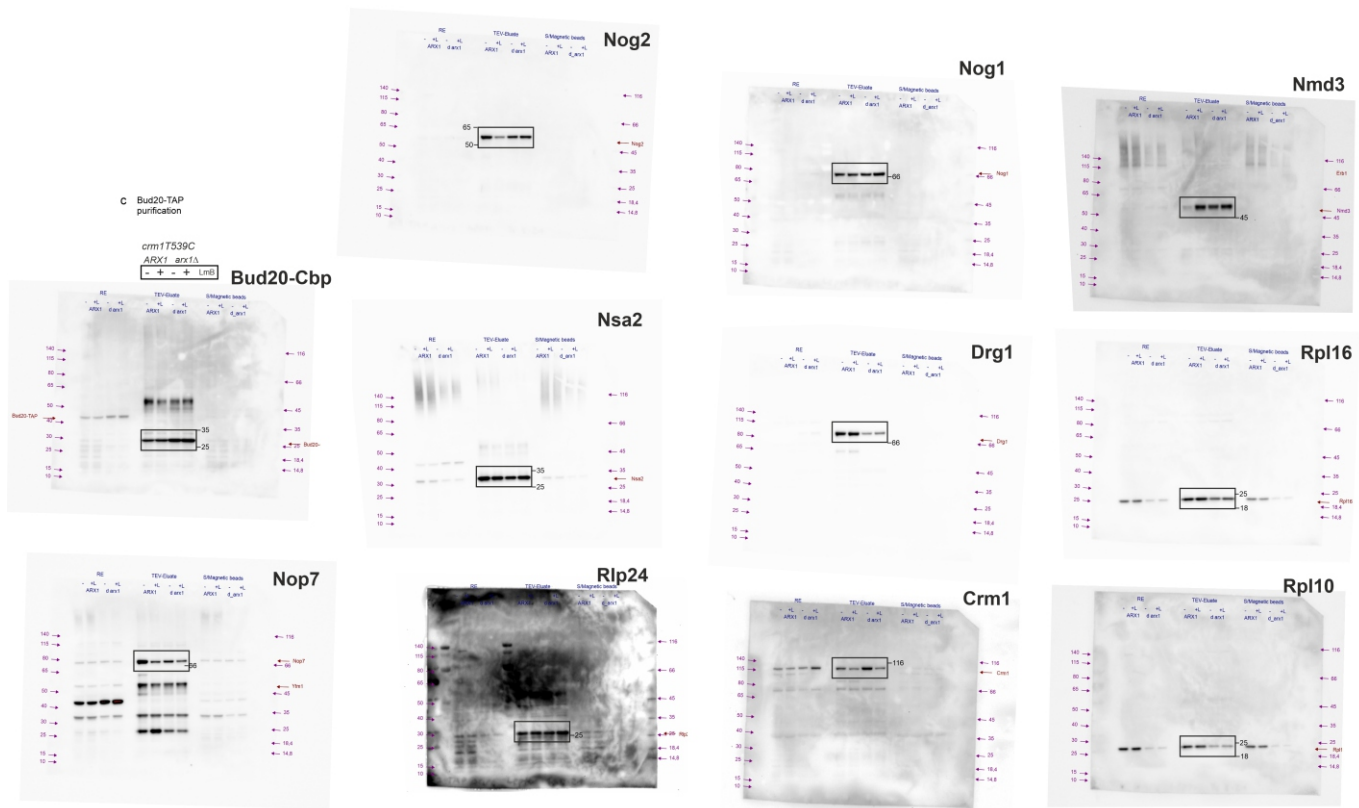

ED Fig3e

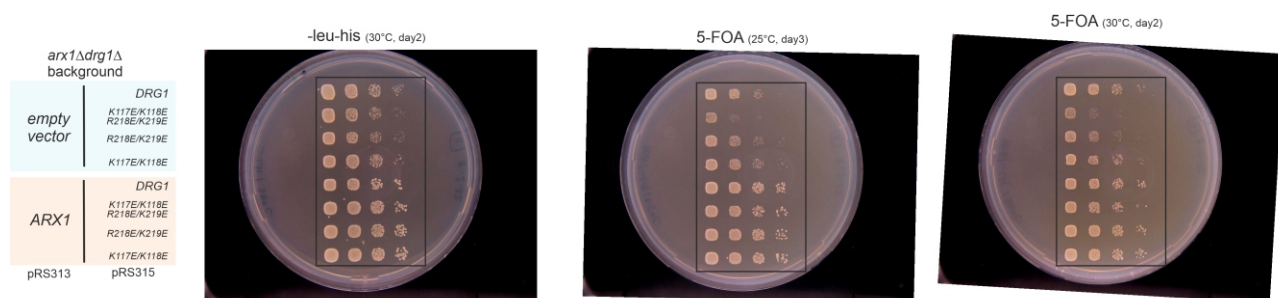

Supplement: Source Data Extended Data Fig. 3b — Uncropped Coomassie gel, Western blots and spot assay images [file 41594_2022_832_MOESM11_ESM.pdf]

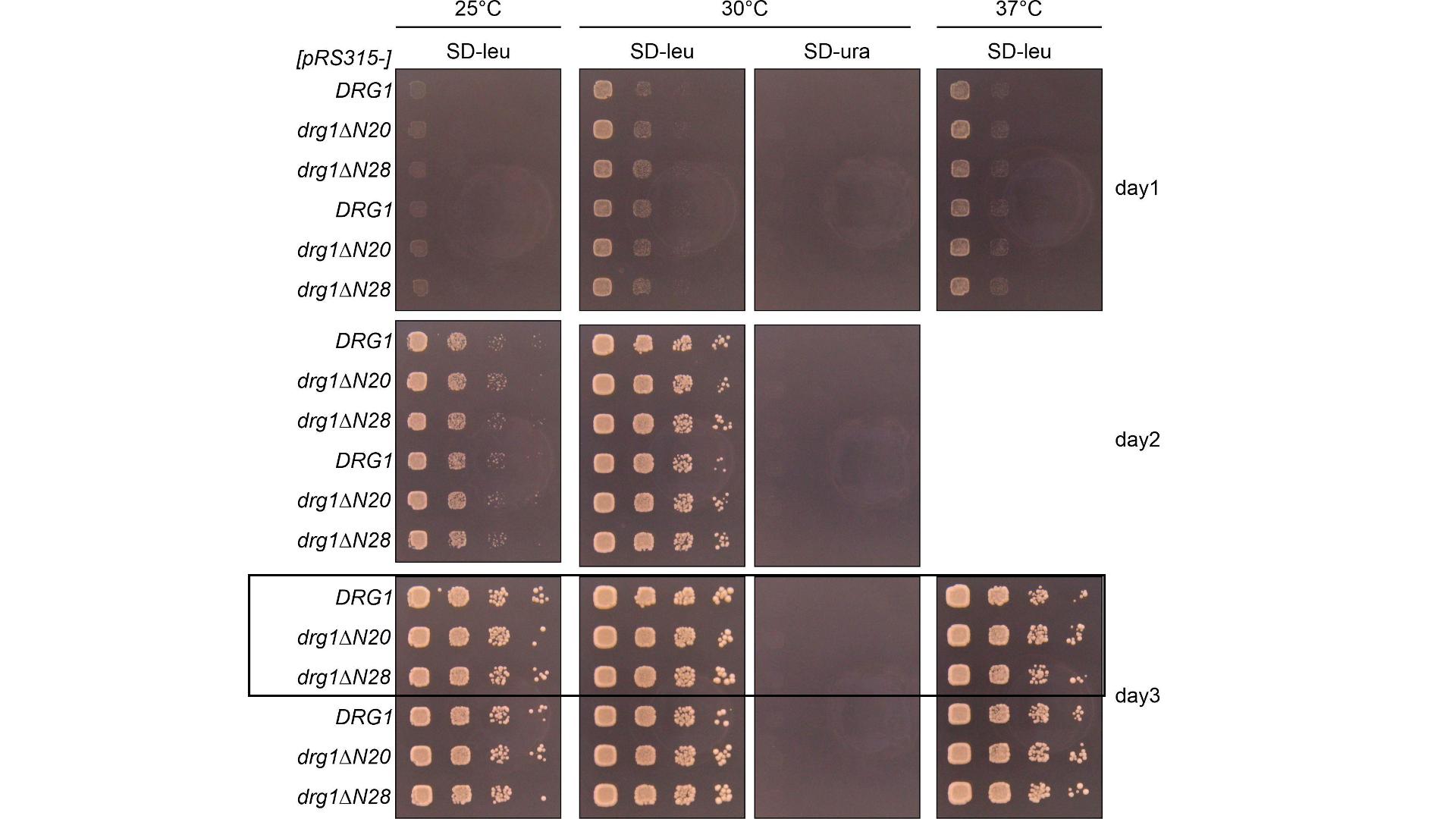

Supplement: Source Data Extended Data Fig. 4 — Uncropped spot assay images [file 41594_2022_832_MOESM13_ESM.jpg]
